# LLM-based multi-agent system for neuro-ophthalmic diagnosis and personalized treatment planning

**DOI:** 10.3389/fnins.2025.1688509

**Published:** 2025-10-06

**Authors:** Wenmiao Wang

**Affiliations:** Department of Electrical and Computer Engineering, National University of Singapore, Singapore, Singapore

**Keywords:** neuro-ophthalmology, multi-agent systems, large language models (LLMs), retinal neurodegeneration, glaucoma, age-related macular degeneration (AMD), multimodal integration, uncertainty-aware diagnosis

## Abstract

**Introduction:**

Ophthalmic findings can non-invasively reflect nervous-system status. We present an LLM-based multi-agent framework that preserves diagnostic uncertainty to support neuro-ophthalmic screening and referral.

**Methods:**

Heterogeneous inputs (clinical text/PDFs and optional fundus/OCT images) are normalized by an Information Collection Agent. A Diagnosis Agent ensembles multiple LLMs and, when available, a CNN image branch; outputs are aggregated with an uncertainty-aware fusion.

**Results:**

Across a curated ophthalmic corpus, the multi-agent framework improves robustness over single-model baselines and produces multi-candidate distributions suitable for downstream triage and monitoring.

**Discussion:**

Uncertainty-aware, multi-candidate predictions align with clinical decision-making under ambiguity and suggest future work on calibration and knowledge-layer fusion.

## 1 Introduction

### 1.1 Background and motivation

The retina is an accessible extension of the central nervous system, making ocular findings informative for brain health and neurodegeneration. Prior research has identified shared mechanisms between age-related macular degeneration (AMD) and neurodegenerative disorders, as well as links between AMD and cognitive decline (Marchesi et al., 2021; Strafella et al., 2021; Baker et al., 2009). Recent multimodal studies further support retinal imaging traits as potential biomarkers for brain abnormalities and neurological disease trajectories (Zhao et al., 2024).

Beyond AMD, neuro-ocular interactions appear in other conditions. Sleep and circadian disruption have been associated with increased glaucoma risk, consistent with the circadian physiology of intraocular pressure and sleep-wake dysregulation (Ciulla et al., 2020; Sun, 2022). Neurocutaneous syndromes such as Sturge-Weber syndrome (encephalotrigeminal angiomatosis) also exemplify the tight coupling of neurological and ocular pathology, often presenting with leptomeningeal angiomas and glaucoma (Singh and Keenaghan, 2025). These converging observations motivate neuro-ophthalmic screening and referral pathways that leverage ophthalmic data to inform nervous-system-relevant decisions—without requiring additional data collection.

Clinically, decision-making frequently integrates heterogeneous inputs (clinical text, PDF reports, fundus or OCT images) under uncertainty. Single-model pipelines that output a single label are often ill-suited to comorbidity and incomplete information. In contrast, retaining multiple differential hypotheses with calibrated probabilities better reflects clinical reasoning and enables downstream personalization.

### 1.2 Problem statement and approach

We address these needs with an LLM-based multi-agent framework focused on ophthalmic data yet designed for neuroscience-oriented screening and referral. The system separates (i) information collection and structuring, (ii) multi-candidate diagnosis, and (iii) planned treatment planning into coordinated agents, preserving uncertainty throughout. A multi-LLM ensemble—optionally combined with a CNN image classifier—produces probability distributions over common retinal conditions; fusion strategies include a rank-based baseline and a reliability-plus-entropy aggregator. While our experiments and datasets remain exclusively ophthalmic, the outputs (ranked hypotheses and calibrated uncertainties) naturally map to neuro-relevant actions, such as risk stratification, monitoring, and referral. Implementation details build on multi-agent principles described in prior research (Li et al., 2024). Evaluation uses standard metrics (Top-*K* accuracy, MRR, ECE/Brier) to quantify diagnostic ranking and calibration on ophthalmology-focused data.

## 2 Related research

Recent progress in large language models (LLMs) and multi-agent collaboration has accelerated the development of medical automation. For a neuroscience audience, ophthalmic data are particularly relevant because the retina is an accessible extension of the central nervous system. Studies report shared genetic/biological mechanisms between age-related macular degeneration (AMD) and neurodegenerative disorders, links between AMD and cognitive decline, eye-brain genetic connections revealed by multimodal imaging genetics, and neuro-ocular interactions such as circadian/sleep disruption associated with glaucoma risk and neurocutaneous syndromes (e.g., Sturge-Weber) with ocular involvement (Strafella et al., 2021; Baker et al., 2009; Zhao et al., 2024; Ciulla et al., 2020; Sun, 2022; Singh and Keenaghan, 2025; Marchesi et al., 2021). These findings motivate neuro-ophthalmic screening and referral built upon ophthalmic inputs—without changing our experiments, which remain solely ophthalmic.

### 2.1 Task decomposition in medical scenarios

A core strategy in multi-agent systems is task decomposition: specialized agents tackle subtasks (evidence retrieval, classification, and decision aggregation) and exchange intermediate outputs. Pandey et al. demonstrate a clinically oriented multi-agent pipeline for medical necessity justification, showing modular scalability and maintainability that map naturally to our *information collection* and *diagnosis* agents (Pandey et al., 2024). Such modularity is germane to neuro-ophthalmic use cases where heterogeneous inputs (clinical text, fundus/OCT images) must be unified while preserving uncertainty.

### 2.2 Retrieval-augmented generation and chain of thought

Retrieval-Augmented Generation (RAG) reduces hallucinations by grounding LLM outputs in retrieved evidence, and Chain-of-Thought (CoT) improves interpretability by externalizing intermediate reasoning. Pandey et al. apply RAG-style decomposition to patient files to enhance complex judgments (Pandey et al., 2024). In our setting, these strategies enable transparent and auditable reasoning logs, suitable for neuro-informed screening/triage even when only ophthalmic data are available.

### 2.3 Bottom-up aggregation and collaborative reasoning

Clinical checklists or hierarchical decision trees benefit from bottom-up aggregation: leaf-level assessments are reconciled at higher-level nodes by an integrating agent. This facilitates traceability and error localization in complex workflows (Pandey et al., 2024). For neuro-ophthalmic contexts—where ocular findings may proxy CNS processes—such aggregation clarifies how each modality contributes to neuro-relevant hypotheses.

### 2.4 Multi-agent reinforcement learning for clinical operations

Beyond diagnosis pipelines, multi-agent reinforcement learning (MARL) addresses resource allocation and concurrent decisions under uncertainty. Plaat summarizes collaborative/adversarial settings and convergence properties, which are relevant to scheduling, triage, and iterative monitoring in neuro-ophthalmic services (Plaat, 2022). While our study does not alter experiments, these principles inform future operational layers around the diagnostic core.

### 2.5 Differential diagnosis and ensemble reasoning

Differential diagnosis aligns with real-world comorbidity and incomplete information. Ensemble reasoning across agents/models can raise accuracy: Barabucci et al. show that combining outputs from multiple LLMs improves diagnostic correctness via collective intelligence (Barabucci et al., 2024). Our multi-LLM ensemble likewise produces ranked hypotheses with calibrated probabilities; these outputs can be mapped to neuroscience-oriented actions such as risk stratification and referral.

### 2.6 Research gaps

Methodologically related research on anomaly detection and multimodel decision strategies under limited labels has appeared in adjacent engineering domains (Zuo et al., 2023, 2024; Zhang et al., 2024). These efforts motivate data-efficient modeling and ensemble-style fusion, consistent with our reliability- and entropy-aware aggregation. Despite progress, gaps remain: (i) limited end-to-end integration that bridges uncertainty-aware differential diagnosis to actionable, neuro-relevant pathways; (ii) underexplored probabilistic fusion across text/imaging/knowledge layers with clinical calibration; and (iii) constrained validation on diverse, real-world datasets. Our framework targets these gaps while keeping experiments ophthalmic, positioning outputs for neuro-ophthalmic screening and follow-up.

## 3 Methods

### 3.1 System overview

#### 3.1.1 Overall design rationale

Ophthalmic findings provide a non-invasive window into the nervous system: genetic and pathophysiologic overlaps have been reported between age-related macular degeneration (AMD) and neurodegenerative disorders, ocular degeneration correlates with cognitive decline, and multimodal imaging genetics reveals eye-brain connections (Strafella et al., 2021; Baker et al., 2009; Zhao et al., 2024; Marchesi et al., 2021). Sleep and circadian disruption are associated with glaucoma risk, and neurocutaneous syndromes such as Sturge-Weber frequently present with ocular involvement (Ciulla et al., 2020; Sun, 2022; Singh and Keenaghan, 2025). Motivated by this neuro-ophthalmic linkage, the system is structured to produce uncertainty-aware, multi-candidate retinal diagnoses that can be mapped to neuroscience-oriented screening, triage, and referral pathways.

#### 3.1.2 High-level architecture

**Figure 1:**
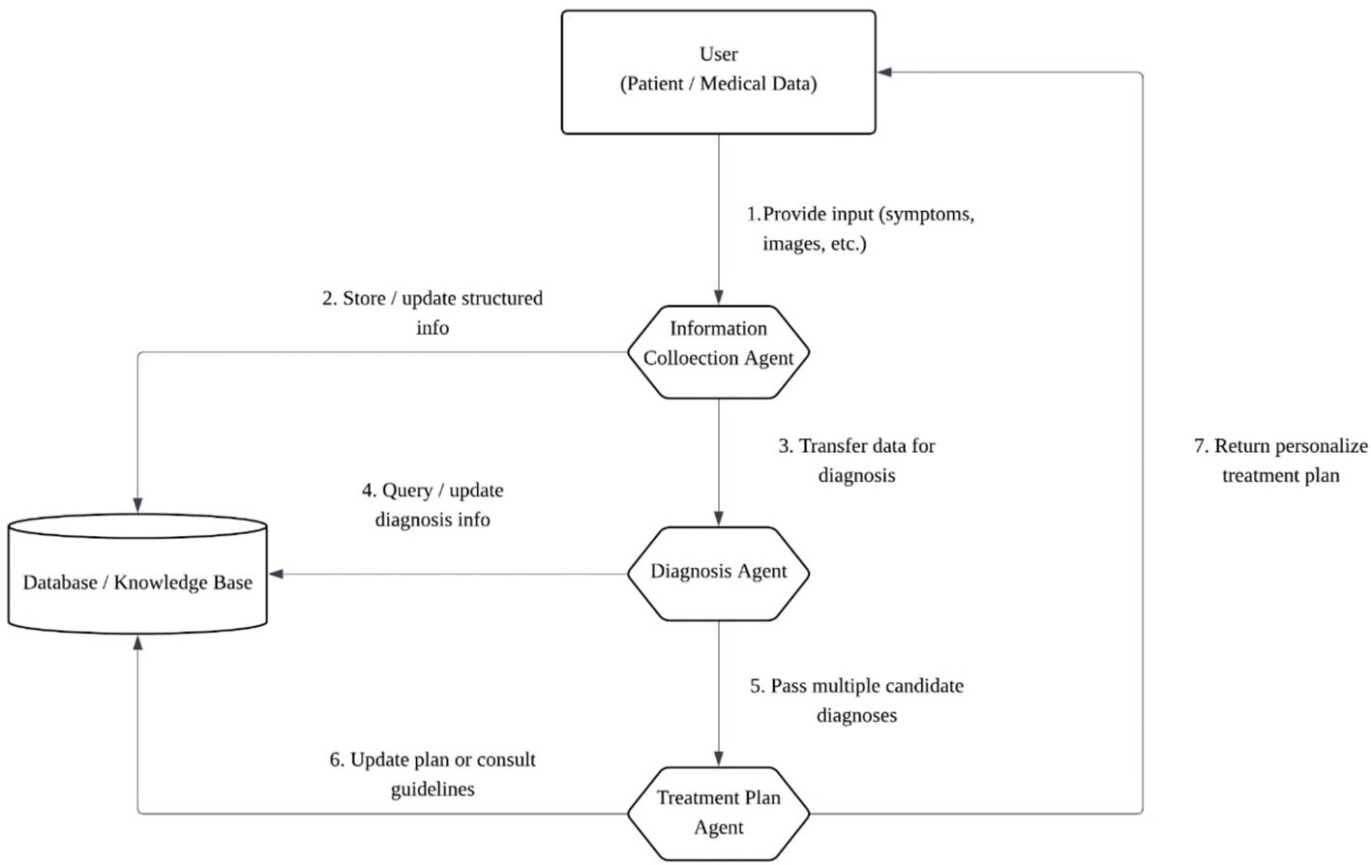
High-level architecture. Heterogeneous inputs are normalized and passed to an ensemble-based diagnosis pipeline; uncertainty-preserving outputs are exposed to a treatment-planning interface for downstream, neuro-relevant actions.

The framework integrates heterogeneous inputs (clinical text/PDFs and fundus/OCT images) and multiple diagnostic submodules within a coordinated multi-agent design. The architecture comprises (i) an *Information Collection Agent* that normalizes inputs, (ii) a *Diagnosis Agent* that generates ranked differential hypotheses with calibrated probabilities via a multi-LLM ensemble, optionally fused with a CNN image classifier, and (iii) a *Treatment Plan Agent* interface prepared for downstream personalization. Prior multi-agent principles for task decomposition, retrieval grounding, and interpretable reasoning inform this design (Pandey et al., 2024; Kumichev et al., 2024). A high-level schematic is shown in Figure 1.

#### 3.1.3 Core objectives

- **Structured data ingestion:** capture and standardize clinical text, PDF documents, and ophthalmic images for reproducible downstream use.
- **Multi-candidate diagnosis:** produce ranked differential hypotheses with associated probabilities through LLM-based textual reasoning and neural image models.
- **Personalization-ready outputs:** present calibrated uncertainties and interfaces that support individualized pathways (screening, monitoring, referral) relevant to neuroscience.

##### 3.1.3.1 Neuro-relevant action mapping

The five-way retinal output {NoDR, MildDR, SevereDR, Glaucoma, AMD} and their calibrated probabilities are aligned with application-oriented pathways: (i) *screening* when the fused probability is low-to-moderate yet non-negligible for conditions with neuro-relevant implications (e.g., glaucoma signals co-occurring with sleep/circadian risk factors); (ii) *monitoring* when intermediate probabilities suggest progression risk (e.g., AMD-compatible patterns with cognitive-risk associations); and (iii) *referral* when high-probability hypotheses implicate optic-nerve or macular involvement requiring specialist evaluation. These mappings are intended as *application-aligned* decision supports rather than validated clinical endpoints, and they explicitly preserve uncertainty to avoid premature single-label commitments.

##### 3.1.3.2 Label-set rationale and neuro-ophthalmic relevance

The five-class set mirrors the distribution of available cases in our corpus and common retinal entities used in screening contexts. Although the experiments remain ophthalmic, these labels can inform neuro-relevant pathways through vascular and neurodegenerative linkages discussed in the background (e.g., microvascular burden, optic neuropathy, and age-related macular pathology). The class set, therefore, balances corpus realism with downstream interpretability without expanding the experimental scope.

#### 3.1.4 Agents and their functionalities

##### 3.1.4.1 Information collection agent

Parses patient descriptions and PDF reports; performs OCR on scans when needed; structures extracted entities (e.g., age, key symptoms, prior treatments), and indexes image data (fundus/OCT). Outputs a normalized representation for downstream modules.

##### 3.1.4.2 Diagnosis agent

Aggregates textual features, imaging-derived scores, and optional knowledge-layer signals. A multi-LLM ensemble is combined with a CNN classifier to produce a probability distribution over common retinal conditions. Fusion includes a rank-based baseline and a reliability-plus-entropy aggregator calibrated on a small subset of training data. The agent returns uncertainty-preserving differential diagnoses suitable for neuro-ophthalmic risk stratification.

##### 3.1.4.3 Treatment plan agent (interface)

Consumes multi-candidate outputs and probabilities to expose a structured interface for potential therapy pathways. While implementation remains conceptual, the interface is designed to incorporate comorbidities, patient preferences, and resource constraints, facilitating translation to neuro-relevant triage and follow-up.

#### 3.1.5 Data flow and agent interaction

**Figure 2:**
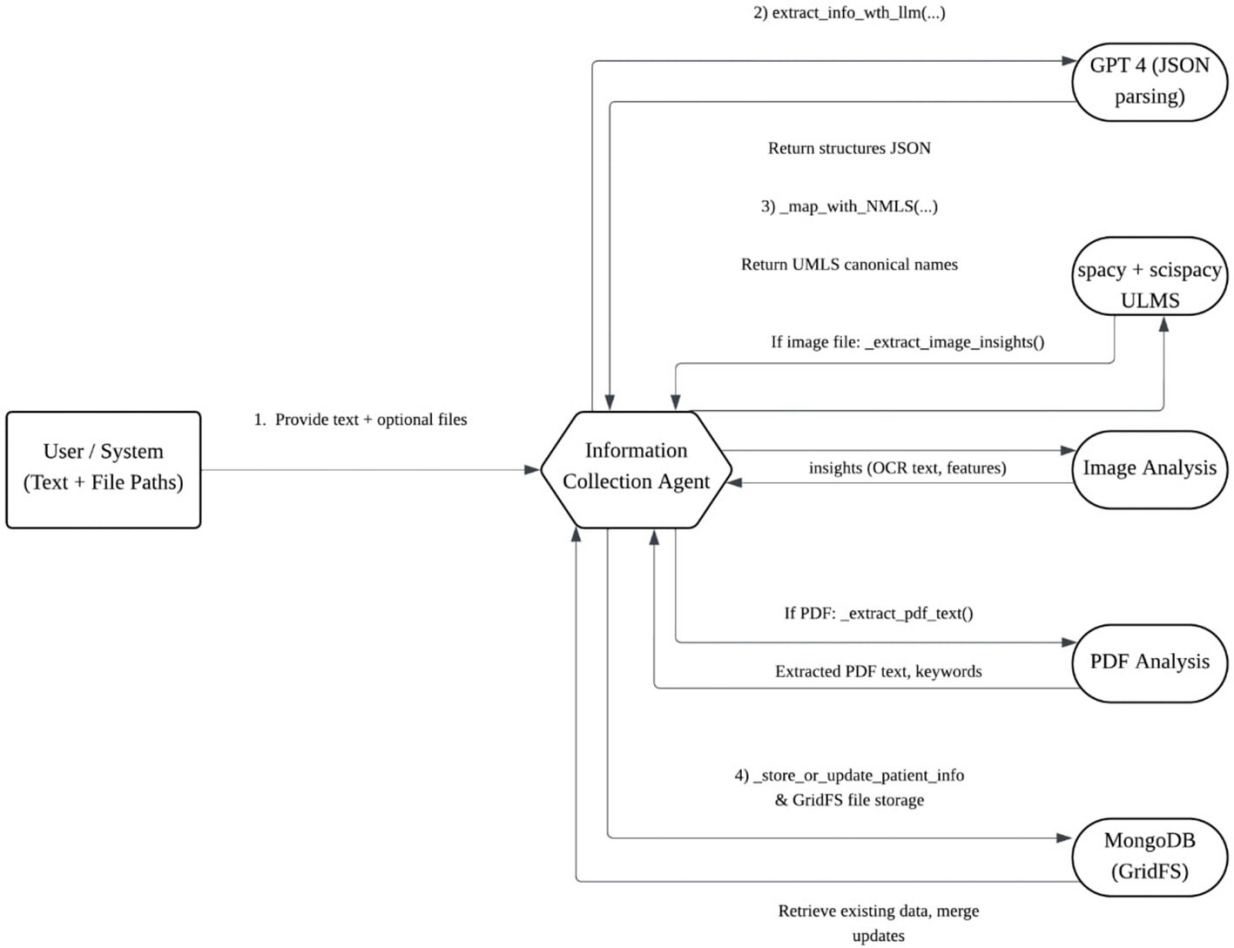
Information flow among agents. Normalized inputs drive ensemble diagnosis; uncertainty-preserving results are exposed to a treatment interface for screening, referral, and monitoring decisions.

Data move through normalized representations and message-passing between agents. A typical sequence is summarized in Figure 2: (1) text/PDFs and fundus/OCT images are ingested; (2) variables are extracted and stored; (3) the diagnosis pipeline performs text reasoning and image classification, returning a ranked set of hypotheses with probabilities; (4) the treatment interface, when enabled, exposes these uncertainty-aware outputs to downstream decision pathways aligned with neuro-ophthalmic use.

#### 3.1.6 Summary

The architecture operationalizes task decomposition and ensemble reasoning (Pandey et al., 2024), with reinforcement-learning principles offering future avenues for operational decision-making around the diagnostic core (Plaat, 2022). By retaining multi-candidate hypotheses and calibrated uncertainties, ophthalmic outputs can be directly leveraged for neuroscience-aligned screening and referral, consistent with eye-brain links documented in the literature.

### 3.2 Information collection agent

#### 3.2.1 Scope and objectives

The agent operationalizes a standardized pathway from heterogeneous clinical inputs to analysis-ready representations. Inputs encompass free-text narratives and PDF reports alongside fundus and OCT images—modalities that, while ophthalmic in focus, bear relevance to nervous-system status given documented eye-brain links and neuro-ocular comorbidities. The objective is to preserve uncertainty while harmonizing entities, measurements, and image-derived descriptors so that downstream modules can compute multi-candidate hypotheses without additional data collection.

#### 3.2.2 Design rationale

Three principles guide the design. *First*, heterogeneous inputs are normalized to a consistent schema, enabling reproducible queries across text and imaging. *Second*, clinical semantics are aligned with canonical concepts to reduce synonymy and spelling variance; this improves fusion with knowledge-based layers. *Third*, artifacts that quantify evidence (e.g., OCR strings, image features, extracted entities) are retained to support auditability and neuro-relevant referral logic.

#### 3.2.3 High-level architecture

**Figure 3:**
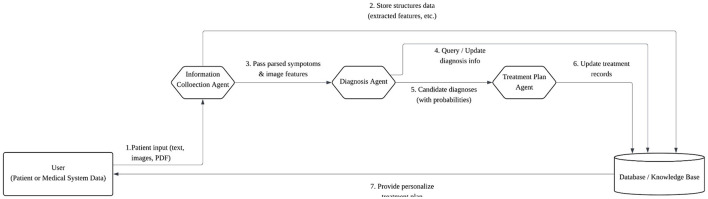
Internal architecture of the Information Collection Agent. Text and PDF streams undergo entity extraction and concept alignment; imaging streams produce OCR text and feature descriptors. All artifacts are stored with stable identifiers for downstream consumption.

The agent comprises coordinated text, imaging, and storage subsystems (Figure 3). Textual pipelines perform entity extraction and concept alignment; imaging pipelines apply OCR to scanned materials and derive visual features from retinal photographs and OCT; storage pipelines archive both raw files and derived artifacts with stable identifiers for subsequent agents.

#### 3.2.4 Text and PDF processing

Structured fields are distilled from narratives and PDF reports using large language models to impose a consistent schema (age, medical history, medications, symptoms, and anatomical descriptors), followed by biomedical entity linking that maps terms to canonical identifiers in the Unified Medical Language System (UMLS) (Bodenreider, 2004). In practice, robust biomedical NLP resources (e.g., scispaCy) provide tokenization, biomedical NER, and linkers that reduce variance across lay and professional phrasing (Neumann et al., 2019). For scanned documents, OCR is applied to extract textual content prior to concept alignment; widely used engines such as Tesseract provide dependable character-level recognition in clinical artifacts (Smith, 2007). The outcome is a normalized text representation that preserves provenance (source file, page, and span) to support auditable reasoning and, when needed, neuro-oriented referral notes grounded in ocular findings.

#### 3.2.5 Imaging streams

Fundus and OCT inputs are transformed into descriptors suitable for fusion with text-derived evidence. OCR is applied when images contain embedded annotations. Visual features are computed using established convolutional backbones (e.g., residual networks) to encode disease-relevant patterns while remaining model-agnostic for subsequent classifiers (He et al., 2016). These features, together with OCR terms (e.g., “drusen,” “hemorrhage, “cup-disc”), are attached to the same patient-level schema as text entities, enabling downstream ranking over common retinal conditions and facilitating interpretations relevant to nervous-system risk where appropriate.

#### 3.2.6 Data model and record management

Each patient record consolidates a stable identifier; basic demographics (when available); standardized clinical history and active medications; symptom descriptors; high-level anatomical tags (e.g., macula, peripapillary region); and a set of attachments (original files plus derived artifacts). Every attachment stores its file identifier and associated insights, including OCR text, image feature vectors, and salient keywords. Record updates follow a non-destructive merge policy: new fields amend the record without overwriting previously verified information. This strategy accommodates iterative clarification and supports the preservation of longitudinal trajectories—important in neuro-ophthalmic monitoring where progressive change carries clinical significance.

#### 3.2.7 Agent interfaces and orchestration

Upstream systems can submit free text, PDFs, and images together with optional metadata. The agent returns: (i) a normalized schema with entity-linked fields; (ii) identifiers for stored files and artifacts; and (iii) a compact summary of text- and image-derived cues suitable for the Diagnosis Agent. Message passing or direct calls are supported, enabling modular integration without exposing implementation details. This interface design ensures that uncertainty in the inputs is preserved and made explicit for subsequent multi-candidate inference and, when enabled, for neuroscience-oriented triage and referral.

#### 3.2.8 Operational quality controls

To maintain reliability across diverse inputs, the agent applies schema validation, minimal completeness checks, and versioned model configurations. Provenance metadata (time, source, and processing versions) persisted with every artifact to facilitate audits and ablation analyses. These practices are particularly relevant when ophthalmic indicators serve as proxies for nervous system processes, where traceability from output back to the original evidence is essential for clinical acceptance.

#### 3.2.9 Information flow

**Figure 4:**
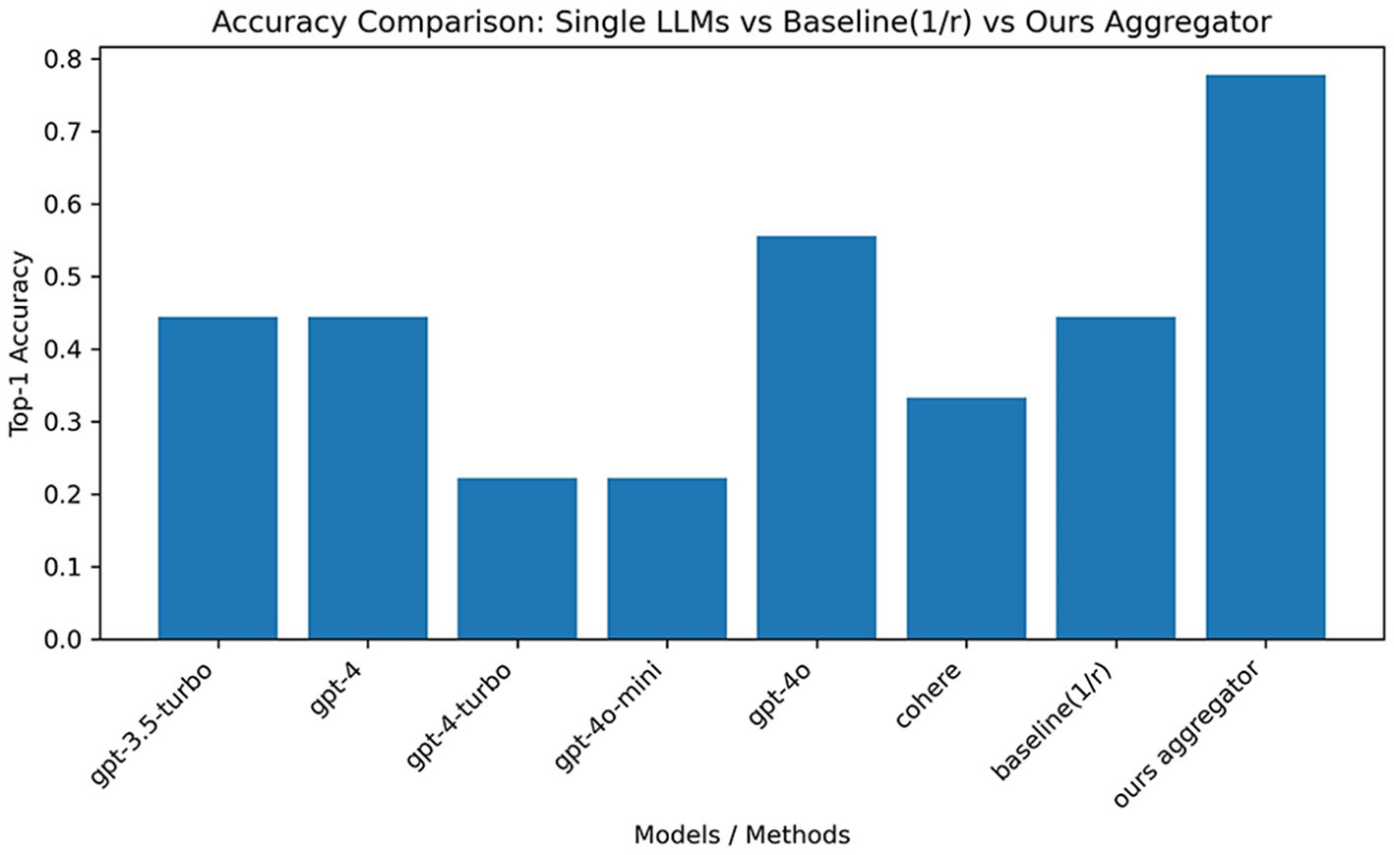
End-to-end data flow. Heterogeneous inputs are standardized and aligned to canonical concepts; images yield OCR strings and visual descriptors; all artifacts are linked under a patient-level record for downstream differential diagnosis and neuro-relevant actions.

A representative flow is visualized in Figure 4: clinical narratives and PDFs are standardized and concept-aligned; fundus/OCT are transformed into OCR strings and visual descriptors; all outputs are stored under patient-level records and surfaced to downstream modules with preserved uncertainty.

#### 3.2.10 Linkage to diagnosis and neuro-oriented use

The Diagnosis Agent consumes the normalized schema, entity-linked fields, OCR terms, and image descriptors to produce ranked, uncertainty-aware hypotheses. These outputs are directly mappable to neuro-ophthalmic actions: prioritizing referral in the presence of optic-nerve-relevant cues, scheduling monitoring when AMD- or glaucoma-associated patterns co-occur with risk factors (e.g., sleep/circadian disruption), and generating auditable summaries that reflect the preserved uncertainty (Ciulla et al., 2020; Sun, 2022; Strafella et al., 2021; Baker et al., 2009).

### 3.3 Diagnosis agent

#### 3.3.1 Motivation and objectives

The Diagnosis Agent transforms normalized clinical inputs—free-text/PDF narratives and fundus/OCT images—into uncertainty-aware, multi-candidate retinal diagnoses. While the dataset and tasks are strictly ophthalmic, the output distributions and calibrated uncertainties naturally support neuroscience-oriented screening and referral, consistent with reported eye-brain links, shared AMD-neurodegeneration mechanisms, cognitive associations, and neuro-ocular comorbidities such as circadian/sleep disruption in glaucoma and ocular involvement in Sturge-Weber syndrome (Strafella et al., 2021; Baker et al., 2009; Zhao et al., 2024; Ciulla et al., 2020; Sun, 2022; Singh and Keenaghan, 2025; Marchesi et al., 2021). The agent pursues two objectives: (i) produce a probability vector over five retinal conditions {NoDR, MildDR, SevereDR, Glaucoma, AMD}; (ii) fuse multiple submodels (LLMs and, optionally, an image classifier) into a single calibrated distribution.

#### 3.3.2 Rationale for LLM-based multi-agent inference over conventional CNN baselines

The present task primarily requires reasoning over *clinical narratives and PDF reports* and only secondarily over ocular images; the majority of records in our corpus are text-only, with retinal/OCT images available for a minority subset. Accordingly, purely image-based pipelines (e.g., stand-alone CNN classifiers) cannot operate on a large fraction of cases and, by design, cannot integrate non-visual evidence such as medications, longitudinal history, or negated symptoms. In contrast, large language models (LLMs) natively consume unstructured clinical text and support retrieval-augmented and stepwise reasoning, which are crucial for uncertainty-aware differential diagnosis across heterogeneous inputs.

Robust CNNs for retinal disease classification typically require *large-scale* labeled image corpora (on the order of tens to hundreds of thousands of images) to generalize reliably across devices and populations; seminal efforts trained and validated on ≥100, 000 fundus photographs for a single screening task. Our dataset is intentionally small and mixed (authentic plus partially synthesized records), with limited image availability, making a text-first, knowledge-transfer approach more suitable than training a data-hungry image model from scratch.

The clinical objective is not a single image label but *uncertainty-preserving, multi-candidate* outputs that can be mapped to actionable pathways (screening, monitoring, referral). LLM ensembles naturally expose calibrated distributions over hypotheses via fusion and can be coupled with retrieval and chain-of-thought methods to surface supporting evidence and rationale. In contrast, a simple CNN baseline—even if available—would neither address text-heavy cases nor provide a unified mechanism for cross-modal evidence integration.

Finally, to accommodate the subset of cases with images, our design includes an *optional* CNN-based image branch whose probabilities participate in the fusion but do not dominate the decision when images are absent. This preserves coverage across the dataset while allowing image cues to contribute where present, without altering the ophthalmic scope of the experiments. With substantially larger image cohorts, we plan to benchmark dedicated CNN/ViT baselines under the same fusion interface.

#### 3.3.3 Overall architecture

**Figure 5:**
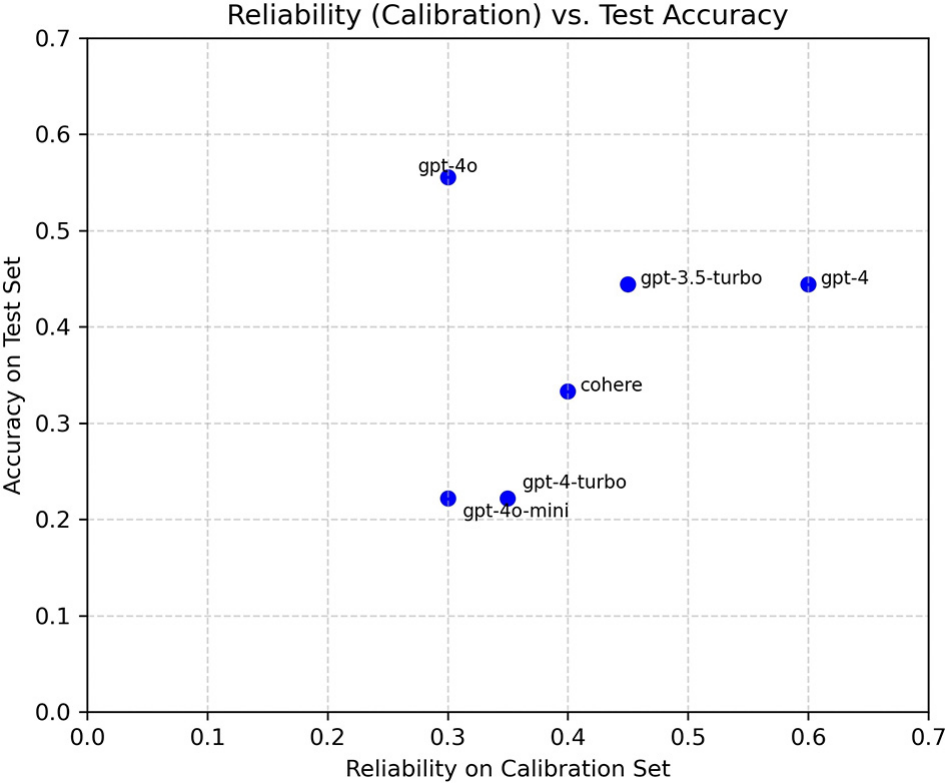
Diagnosis Agent architecture. Multiple textual LLMs (and an optional image classifier) produce per-disease distributions that are fused into a final uncertainty-aware diagnosis.

The agent integrates textual reasoning from several large language models (LLMs) and, when available, an image classifier; all submodel outputs are merged by a downstream fusion module. Prior evidence suggests that ensemble reasoning across complementary models can improve diagnostic robustness (Barabucci et al., 2024), and multi-agent task decomposition informs the orchestration (Pandey et al., 2024). A schematic overview is shown in Figure 5.

#### 3.3.4 Problem statement

Let *x* = {*x*_text_, *x*_pdf_, *x*_img_} denote the available inputs for a case, where any component may be absent. Let the retinal label set be C={NoDR,MildDR,SevereDR,Glaucoma,AMD}. The objective is to estimate a calibrated probability vector $p_{\theta }\left(y\in C|x\right)\in {\left[0,1\right]}^{5}$ with $\underset{c\in C}{\sum }p_{\theta }\left(c|x\right)=1$, preserving diagnostic uncertainty for downstream screening/monitoring/referral. Submodels (LLMs and, when present, an image classifier) each produce *p*_*i*_(·∣*x*); fusion yields $\hat{p}\left(\cdot |x\right)$.

#### 3.3.5 Input specification and data partitioning

Case is represented by a unique identifier, a free-text description (or PDF-derived narrative), an optional path to a fundus/OCT image, and a ground-truth label from {NoDR, MildDR, SevereDR, Glaucoma, AMD}. Records are split into a small calibration subset (used solely to estimate submodel reliability), a training/validation portion (for internal development of prompts and settings), and a held-out test set for final reporting. The split uses a fixed random seed to ensure reproducibility, and the calibration subset size matches the reliability-estimation budget described below.

#### 3.3.6 Probabilistic inference with an LLM ensemble

For each disease candidate *d*, each LLM receives a succinct, controlled prompt that elicits a response conditioned on *d*. A heuristic “pseudo-loss” is derived from the response length:


$$\ell \left(d\right)=\frac{\text{len}\left(\text{respons}{\text{e}}_{d}\right)}{10.0},\text{ conf}\left(d\right)=\frac{1}{\ell \left(d\right)+\epsilon },$$

followed by normalization across the five candidates to yield a per-LLM distribution **p**(*d*). Although unconventional, this surrogate differentiates relative model reactivity without requiring the LLM to declare explicit probabilities; it is used here strictly as an internal scoring device while preserving the experimental design and dataset scope. Multiple LLMs produce multiple such distributions.

#### 3.3.7 Optional image evidence

When an image is available, a convolutional backbone (e.g., a residual network) produces a five-way probability vector aligned with the same disease set. The classifier operates independently of textual pipelines; its output is treated as an additional submodel. If the image is unavailable or invalid, the textual ensemble proceeds alone. This decoupling keeps the ophthalmic experiments intact while allowing future neuro-relevant extensions where ocular biomarkers inform central nervous system risk.

#### 3.3.8 Fusion strategies: rank-based baseline (1/r)

For each submodel, diseases are ranked by descending probability. If disease *d* has rank *r* in a given submodel, a fractional weight 1/(*r* + 1) is added to *d*'s cumulative score; scores are then normalized across diseases. This baseline ignores submodel quality but provides a transparent reference (Barabucci et al., 2024).

#### 3.3.9 Fusion strategies: reliability-plus-entropy aggregator

To account for heterogeneous submodel quality and certainty, the aggregator assigns each submodel *i* a reliability α_*i*_ and applies an entropy penalty to its distribution. Reliability α_*i*_ is estimated on a disjoint calibration subset as the top-1 match rate. Let *H*_*i*_ be the Shannon entropy of submodel *i*'s five-way distribution and *H*_max_ = ln 5. The final unnormalized score for disease *d* is


$$\text{Score}\left(d\right)=\overset{N}{\underset{i=1}{\sum }}\left[\alpha_{i}\times p_{i}\left(d\right)\times \left(1-\frac{H_{i}}{H_{\max}}\right)\right],$$

followed by normalization (e.g., dividing by $\underset{d}{\sum }\text{Score}\left(d\right)$) to obtain the fused probability. Submodels that are both reliable and low-entropy contribute more; high-entropy (uncertain) distributions are down-weighted. This design preserves the original experiments (including the calibration budget and candidate set) while improving robustness.

##### 3.3.9.1 Missing-data handling and fusion neutrality

Let $S_{i}$ be the set of submodels available for case *i* (LLMs and, when present, the image branch). Denote each submodel's calibrated distribution as **p**^(*j*)^ with weight *w*_*j*_ (reliability and entropy-aware). The fused score for class *c* is


$$s_{i}\left(c\right)=\underset{j\in S_{i}}{\sum }w_{j}p^{\left(j\right)}\left(c\right),\;{\hat{p}}_{i}\left(c\right)=\frac{s_{i}\left(c\right)}{\sum_{c^{\prime }}s_{i}\left(c^{\prime }\right)}.$$

If the image branch is missing, it is simply excluded from $S_{i}$ and weights are re-normalized over the remaining submodels. Thus, class probabilities are not biased by the absence of images beyond the removal of that single evidence source.

#### 3.3.10 Calibration and evaluation protocol

Calibration uses a small, fixed subset drawn from the training portion to compute α_*i*_ for each submodel; these values are *not* tuned on the test set. Final evaluation proceeds on the held-out test cases: (i) per-LLM single-model top-1 accuracy; (ii) the rank-based fusion accuracy; and (iii) the reliability-plus-entropy fusion accuracy. Ancillary diagnostics retain the vector of α_*i*_ values and summary descriptors of submodel entropies for auditability. No additional data are collected, and no label set is altered.

#### 3.3.11 Information flow and outputs

Given a case, the agent returns: (i) per-submodel probability vectors; (ii) a fused five-way distribution; and (iii) ranked hypotheses with calibrated uncertainties. These outputs are directly consumable by downstream personalization interfaces and can be mapped to neuroscience-oriented actions, for example, prioritizing referral when optic-nerve-relevant cues dominate the fused distribution or scheduling monitoring when AMD-linked patterns co-occur with risk factors associated with cognitive decline or circadian/sleep disruption (Baker et al., 2009; Ciulla et al., 2020; Sun, 2022).

#### 3.3.12 Contributions

The Diagnosis Agent (i) formalizes multi-candidate ophthalmic diagnosis using an ensemble of textual LLMs and an optional image classifier; (ii) introduces a reliability-plus-entropy fusion that weights submodels by calibration-informed quality and predictive certainty; and (iii) exposes uncertainty-preserving outputs that are readily interpretable for neuro-ophthalmic screening and referral. The approach leaves the experimental setup unchanged while elevating robustness over single-model baselines (Barabucci et al., 2024; Ren et al., 2023; Pandey et al., 2024; Kumichev et al., 2024).

## 4 Experiments and results

### 4.1 Experimental setting

The evaluation quantifies the performance of the Diagnosis Agent on two hundred ophthalmic cases (authentic plus partially augmented). The study examines single-model behavior across several large language models (LLMs) and, when available, an image-based submodel; it then compares two fusion strategies (a rank-based baseline vs. a reliability-plus-entropy aggregator). Although the dataset and labels are strictly ophthalmic, the analysis highlights how uncertainty-preserving, multi-candidate outputs can support neuroscience-oriented screening, triage, and referral in light of established eye-brain links and neuro-ocular comorbidities. No on-premise training is performed. The per-case runtime is dominated by parallel API calls to a small set of LLMs; when present, the image branch performs a single forward pass of a lightweight CNN. Hence, latency is primarily governed by API round-trip time rather than local compute.

### 4.2 Dataset

A total of 200 cases were assembled from publicly accessible ophthalmic sources and educational repositories (Review of Ophthalmology, 2025; Yiu, 2025; Digital Journal of Ophthalmology, 2025; University of Iowa Department of Ophthalmology and Visual Sciences, 2025), with controlled text augmentation guided by clinical priors. Each record contains a free-text narrative, an optional fundus/OCT image, and a ground-truth label from the five-class set {NoDR, MildDR, SevereDR, Glaucoma, AMD}. The corpus intentionally includes variable narrative length and detail to reflect practical heterogeneity. Only a minority subset has valid images; most entries are text-only. Class counts are moderately balanced to reduce trivial majority effects. Because part of the corpus is augmented, the reported metrics characterize the behavior of the proposed agent under controlled but non-exhaustive conditions rather than constituting definitive clinical performance.

#### 4.2.1 Modality coverage

Most entries in the corpus are text-only clinical narratives and PDF-derived notes; only a minority subset includes valid retinal/OCT images. This distribution motivates a text-first, LLM-centric design capable of operating across all cases, with imaging treated as an optional evidence source rather than a universal input.

#### 4.2.2 Augmentation safeguards and leakage controls

To avoid leakage, augmentation prompts never exposed gold labels verbatim and used neutral descriptors rather than label tokens. Real educational cases were de-identified prior to use. We screened augmented texts to remove exact or near-duplicate variants of evaluation items and audited prompt templates to ensure no label names were embedded. These safeguards reduce inadvertent information leakage while preserving the clinical plausibility of narratives.

### 4.3 Models and conditions

Six LLM variants were evaluated, yielding per-case five-way distributions. When an image is present, a convolutional backbone produces an aligned five-way distribution and is treated as an additional submodel. Data were partitioned with a fixed random seed into a small calibration subset, a development (train/validation) portion, and a held-out test set. The calibration subset (20 examples) is used solely to estimate submodel reliability (top-1 match rate) for the fusion aggregator; it is disjoint from the test set.

### 4.4 Evaluation protocol

The protocol reports per-model top-1 accuracy on the test set, along with the two fusion strategies: (i) the rank-based baseline (1/r), which aggregates ranks without model quality information, and (ii) the reliability-plus-entropy aggregator, which weights each submodel by its calibration reliability and down-weights high-entropy (uncertain) distributions. For transparency and auditability, we summarize per-LLM calibration reliability and test accuracies and compare them with the two fusion results (Tables 1, 2).

**Table 1:** Reliability on the calibration subset (20 cases).

**LLM**	**Reliability (Calibration)**	**Remark**
gpt-3.5-turbo	0.45	Midrange
gpt-4	0.60	Highest among LLMs
gpt-4-turbo	0.35	Lower reliability
gpt-4o-mini	0.30	Low reliability
gpt-4o	0.30	Contrasts with higher test success
cohere	0.40	Moderate

**Table 2:** Top-1 accuracy on the held-out test set.

**Method**	**Test accuracy**
gpt-3.5-turbo	0.4444
gpt-4	0.4444
gpt-4-turbo	0.2222
gpt-4o-mini	0.2222
gpt-4o	0.5555
cohere	0.3333
**baseline (1/r)**	0.4444
**reliability-plus-entropy**	0.7777

#### 4.4.0.1 Prompting and reproducibility

All runs pinned specific model identifiers and logged provider/API access dates. A low-variance decoding regime (low temperature with default/top-*p* constraints) was used to ensure consistent outputs across prompts; when a seed parameter was available, it was fixed; otherwise, multiple evaluations were averaged as indicated. Prompt templates remained constant across models and experiments; full templates and model identifiers are provided in the Supplementary material. We used a low-variance decoding regime (low temperature with default/top-*p* constraints), pinned model identifiers, and logged provider/API access dates; seeds were fixed where available. Prompt templates were consistent across all conditions.

### 4.5 Metric definitions and reporting conventions

**Top-*****k*** **accuracy** is the fraction of cases where the ground-truth label appears within the top-*k* ranked hypotheses of the fused distribution. **MRR** (mean reciprocal rank) averages 1/rank(true) over cases. **ECE** (expected calibration error) bins predicted probabilities and aggregates the absolute gap between empirical accuracy and mean confidence per bin. **Brier score** is the mean squared error between the predicted probability vector and the one-hot ground truth. Unless stated otherwise, we report point estimates on the held-out test set with fixed random seeds for splits and calibration subsets. Given the modest sample size, we avoid potentially unstable confidence intervals and instead discuss uncertainty qualitatively in the Limitations.

Given predicted probabilities ${\hat{\text{p}}}_{i}\in {\mathbb{R}}^{C}$ and true label *y*_*i*_, for *i* = 1, …, *N*:


$$\text{Top-}k=\frac{1}{N}\overset{N}{\underset{i=1}{\sum }}⊮\left\{y_{i}\in \text{TopK}\left({\hat{\text{p}}}_{i}\right)\right\}.$$

$$\text{MRR}=\frac{1}{N}\overset{N}{\underset{i=1}{\sum }}\frac{1}{\text{ran}{\text{k}}_{{\hat{\text{p}}}_{i}}\left(y_{i}\right)}.$$

For calibration, we report Expected Calibration Error (ECE) with *M* confidence bins *B*_*m*_:


$$\text{ECE}=\overset{M}{\underset{m=1}{\sum }}\frac{\left|B_{m}\right|}{N}\left|\text{acc}\left(B_{m}\right)-\text{conf}\left(B_{m}\right)\right|,$$

where acc(*B*_*m*_) is the empirical accuracy and conf(*B*_*m*_) is the mean predicted confidence within *B*_*m*_ (Guo et al., 2017). For multiclass probabilistic accuracy, we report the Brier score:


$$\text{Brier}=\frac{1}{N}\overset{N}{\underset{i=1}{\sum }}\overset{C}{\underset{c=1}{\sum }}({\hat{p}}_{i,c}-⊮\left\{y_{i}=c\right\}{)}^{2}\text{ Brier}(1950).$$

Brier (1950)

Unless stated otherwise, Top-1 and Top-3 are reported as proportions; MRR, ECE, and Brier are reported on [0, 1] (lower is better for ECE and Brier).

### 4.6 Results

**Figure 6:**
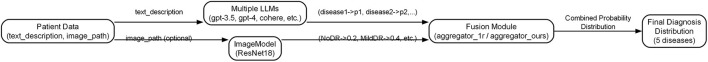
Calibration reliability (20-case subset) vs. test accuracy across LLMs. Differences reflect small-sample effects and sensitivity to stylistic variation in augmented narratives.

**Figure 7:**
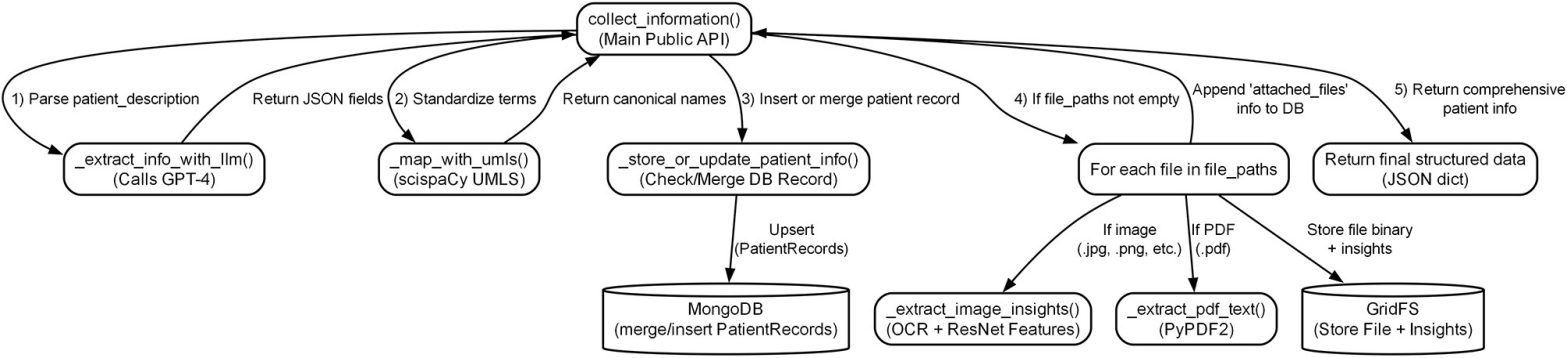
Test-set comparison among single LLMs, the rank-based baseline (1/r), and the reliability-plus-entropy aggregator. The aggregator yields the highest top-1 accuracy.

Single-LLM accuracies vary substantially across the six models, with the best individual accuracy around 0.56 and the weakest near 0.22 on the held-out test set. The rank-based baseline achieves roughly 0.44, tying mid-performing single models and failing to surpass the best LLM. In contrast, the reliability-plus-entropy aggregator attains ~0.78, surpassing all single models and the rank-based baseline. These outcomes are visualized in Figures 6, 7, and detailed in Tables 1, 2.

### 4.7 Analysis and neuro-relevant interpretation

The multi-candidate, uncertainty-preserving outputs are amenable to neuroscience-aligned use without altering the experimental scope. For example, higher fused probabilities on Glaucoma in the presence of specific narrative or imaging cues can prioritize referrals where sleep/circadian comorbidity may modulate risk (Ciulla et al., 2020; Sun, 2022); similarly, AMD-linked patterns can motivate cognitive monitoring in appropriate populations (Baker et al., 2009). Because the aggregator elevates confident and reliable submodels while damping uncertain ones, the resulting distributions are better suited to risk stratification and follow-up scheduling than single-label outputs.

#### 4.7.0.2 Qualitative error modes

Common errors included: (i) brittle handling of negation or hedging (“no prior glaucoma” vs. “rule out glaucoma”); (ii) synonymy and rare term usage; (iii) drift in long narratives where late details outweigh earlier constraints; (iv) OCR artifacts in scanned notes; and (v) variance from small calibration sets when estimating submodel reliability. These patterns contextualize quantitative results and suggest targeted future mitigations (e.g., negation-aware parsing and larger calibration pools).

### 4.8 Summary

The ensemble framework delivers substantial gains on the five-way ophthalmic task under mixed authentic/augmented conditions. The reliability-plus-entropy aggregator improves top-1 accuracy to about 0.78, outperforming the best single model (~0.56) and the rank-based baseline (~0.44), while producing uncertainty-aware outputs that align with neuro-ophthalmic screening and referral pathways already supported by the literature.

## 5 Discussion

### 5.1 Neuro-ophthalmic relevance of uncertainty-aware, multi-candidate outputs

The proposed framework retains ranked hypotheses with associated probabilities rather than enforcing a single label. Although the evaluation is strictly ophthalmic, such uncertainty-aware outputs align with how ocular findings can inform nervous-system assessment. Prior research documents shared mechanisms between age-related macular degeneration (AMD) and neurodegenerative disorders, as well as associations with cognitive decline. Additionally, it highlights eye-brain links from multimodal imaging genetics, sleep/circadian disruption associated with glaucoma risk, and neurocutaneous conditions (e.g., Sturge-Weber) with ocular involvement. Within this context, a probability vector over {NoDR, MildDR, SevereDR, Glaucoma, AMD} can support neuroscience-oriented screening and referral decisions without any change to the underlying ophthalmic experiments.

### 5.2 Interpreting the ensemble gains

On the 200-case corpus, single-model accuracies vary (~0.22–0.56), the rank-based baseline reaches ~0.44, and the reliability-plus-entropy fusion attains ~0.78. These results are consistent with the proposition that ensembling heterogeneous reasoning systems improves robustness (Barabucci et al., 2024), and with multi-agent task decomposition principles that enable modular integration and auditing (Pandey et al., 2024). The reliability term prioritizes submodels that demonstrate higher calibration accuracy on a disjoint subset, while the entropy factor down-weights near-uniform (uncertain) distributions. Together, these components mitigate overreliance on any single LLM's idiosyncrasies or sensitivity to partially augmented text, yielding fused predictions that better reflect aggregate evidence.

### 5.3 From uncertainty to personalization

Multi-candidate diagnosis is not an end in itself; it provides structure for individualized actions. In neuro-ophthalmic settings, higher fused probabilities on Glaucoma could prioritize referral and longitudinal monitoring where sleep/circadian comorbidity may modulate risk (Ciulla et al., 2020; Sun, 2022). Patterns compatible with AMD can motivate cognitive status tracking in appropriate populations (Baker et al., 2009). Because the system preserves a full distribution rather than a single label, downstream tools (or clinicians) may stage investigations, counsel patients, and schedule follow-ups proportional to both likelihood and potential harm, while keeping alternative hypotheses visible.

### 5.4 Positioning relative to existing approaches

Many personalized pipelines presuppose a confirmed single diagnosis; uncertainty is handled implicitly or deferred. By contrast, the present design foregrounds differential diagnosis and explicit uncertainty as first-class outputs, a stance that is closer to routine clinical reasoning. The ensemble formulation also offers a practical resolution to variability among foundation models: instead of adjudicating a priori which model is “best,” the system calibrates contributions and lets the fused distribution reflect reliability and certainty empirically. This is particularly pertinent for ophthalmic narratives of uneven length and style, where single-model behavior can be unstable.

### 5.5 Clinical integration and usability

Translational value hinges on how information is exposed. Probability vectors should be presented with concise rationales and thresholds tuned for screening vs. referral. Interfaces that summarize leading hypotheses, highlight decisive cues (e.g., optic nerve terminology, macular descriptors), and surface provenance (text spans, image descriptors) may facilitate acceptance in time-constrained settings. Because the agent retains artifacts from the ingestion stage, auditability is preserved, enabling focused review when neuro-relevant actions are contemplated.

### 5.6 Limitations

Several constraints bound interpretation and generalizability. First, the dataset is modest (200 cases in total, with ~60 held out for testing), and it includes a portion of model-augmented records; small-sample variance can therefore affect calibration estimates and submodel ranking. Second, image coverage is limited—most records are text-only—so reported conclusions primarily reflect text-based reasoning rather than fully balanced multi-modal fusion. Third, the LLM-derived scoring mechanism used here (a length-based “pseudo-loss”) is a pragmatic heuristic for relative weighting but does not constitute a formally calibrated probability estimator. Fourth, several planned components remain conceptual: the Treatment Plan Agent has not been implemented, and proposed Bayesian/knowledge-graph layers are not yet integrated; these omissions constrain the system's ability to perform deeper causal reasoning or to output ready-to-execute personalized treatment plans. Fifth, evaluation has focused on top-1 accuracy; a comprehensive assessment of differential-diagnosis performance also requires calibrated uncertainty metrics and top-*k* coverage. Finally, no prospective clinical evaluation or outcome study was performed; neuro-ophthalmic interpretations in this study are therefore presented as application-aligned use cases rather than validated clinical endpoints. Collectively, these limitations motivate a prioritized follow-up study on larger, multi-center datasets; richer imaging coverage; formal calibration procedures; and clinician-in-the-loop validation.

### 5.7 Responsible use and claim scope

This system is designed for screening/triage decision support under clinician oversight. Given the small, partially augmented dataset and ophthalmic scope, the outputs are not clinical diagnoses and should not be used to guide treatment without expert review. Prospective, multi-center evaluation and broader datasets will be required before considering translational deployment.

### 5.8 Key insights

The study demonstrates that preserving differential diagnoses and calibrating contributions across heterogeneous submodels yields substantive improvements over single-model pipelines while producing outputs that naturally support neuro-ophthalmic screening and referral. By treating uncertainty as a signal rather than noise, the framework offers a practical route to align ophthalmic AI with neuroscience-informed care pathways within the declared scope and limitations of the current dataset.

## 6 Conclusion

This study presents a modular multi-agent framework that integrates clinical narratives and PDFs, as well as fundus and OCT signals, to produce uncertainty-aware, multi-candidate retinal diagnoses. A reliability-plus-entropy fusion of multiple large language models (optionally augmented by image evidence) improved top-1 accuracy on a 200-case corpus from the best single model (~0.56) and a rank-based baseline (~0.44) to ~0.78, while preserving the ophthalmic scope of the experiments. Because ocular findings can reflect nervous-system status (e.g., shared AMD-neurodegeneration mechanisms, cognitive correlates, circadian/sleep associations with glaucoma, and neurocutaneous syndromes with ocular involvement), the preserved diagnostic distributions are naturally suited for neuroscience-oriented screening, triage, and referral (Strafella et al., 2021; Baker et al., 2009; Zhao et al., 2024; Ciulla et al., 2020; Sun, 2022; Singh and Keenaghan, 2025; Marchesi et al., 2021).

The observed gains from calibrated ensembling and explicit uncertainty point to a compact set of next steps that do not alter the present experimental design: operationalize and validate a Treatment Plan Agent that maps ranked hypotheses to staged actions (screening, monitoring, referral); scale validation to multi-center, real-world corpora with richer imaging to assess generalization and top-*k* coverage; replace or augment the heuristic scoring with formal calibration (e.g., ECE, Brier score) and report per-model reliability and top-*k* metrics; integrate domain-aware reasoning layers (Bayesian networks or knowledge graphs) to encode co-occurrence priors and improve interpretability; conduct clinician-centered studies on probability presentation and provenance to minimize cognitive load; and establish governance, bias assessment, and monitoring protocols for safe deployment.

By treating uncertainty as a signal and calibrating contributions across heterogeneous submodels, the framework goes beyond single-model pipelines to produce outputs that are directly actionable for neuroscience-informed care pathways. The declared limitations (dataset size, limited imaging, partial augmentation, heuristic scoring, and undeveloped treatment/causal modules) highlight the immediate priorities: operational treatment planning, principled reasoning layers, rigorous calibration, and large-scale clinician-linked validation to connect uncertainty-preserving diagnostics with measurable clinical impact.

## Data Availability

The datasets presented in this study can be found in online repositories. The names of the repository/repositories and accession number(s) can be found in the article/Supplementary material.

## References

[B1] Baker M. L. Wang J.-J. Rogers S. Klein R. Kuller L. H. Larsen E. K.. (2009). Early age-related macular degeneration, cognitive function, and dementia: the cardiovascular health study. Arch. Ophthalmol. 127, 667–673. 10.1001/archophthalmol.2009.30 19433718 PMC3001290

[B2] Barabucci G. Shia V. Chu E. Harack B. Fu N. (2024). Combining insights from multiple large language models improves diagnostic accuracy. arXiv preprint arXiv:2402.08806 [cs.AI]. Available online at: https://arxiv.org/abs/2402.08806

[B3] Bodenreider O. (2004). The unified medical language system (UMLS): integrating biomedical terminology. Nucleic Acids Res. 32, D267–D270. 10.1093/nar/gkh061 14681409 PMC308795

[B4] Brier G. W. (1950). Verification of forecasts expressed in terms of probability. Mon. Weather Rev. 78, 1–3.33502177

[B5] Ciulla L. Moorthy M. Mathew S. Siesky B. Verticchio Vercellin A. C. Price D.. (2020). Circadian rhythm and glaucoma: what do we know? J. Glaucoma 29, 127–132. 10.1097/IJG.0000000000001402 31693644

[B6] Digital Journal of Ophthalmology (2025). Journal Homepage. Available online at: https://djo.harvard.edu/ (Accessed September 24, 2025).

[B7] Guo C. Pleiss G. Sun Y. Weinberger K. Q. (2017). “On calibration of modern neural networks,” in Proceedings of the 34th International Conference on Machine Learning (ICML 2017) (Sydney, NSW : Proceedings of Machine Learning Research), 1321–1330.

[B8] He K. Zhang X. Ren S. Sun J. (2016). “Deep residual learning for image recognition,” in Proceedings of the IEEE Conference on Computer Vision and Pattern Recognition (CVPR) (Las Vegas, NV: IEEE Computer Society), 770–778.

[B9] Kumichev G. Blinov P. Kuzkina Y. Goncharov V. Zubkova G. Zenovkin N. (2024) MedSyn: LLM-based synthetic medical text generation framework. arXiv preprint arXiv:2408.02056 [cs.CL]. Available online at: https://arxiv.org/abs/2408.02056.

[B10] Li J. Lai Y. Li W. Ren J. Zhang M. Kang X.. (2024). Agent Hospital: s simulacrum of hospital with evolvable medical agents. arXiv preprint arXiv:2405.02957 [cs.AI]. Available online at: https://arxiv.org/abs/2405.02957

[B11] Marchesi N. Fahmideh F. Boschi F. Pascale A. Barbieri A. (2021). Ocular neurodegenerative diseases: interconnection between retina and cortical areas. Cells 10:2394. 10.3390/cells10092394 34572041 PMC8469605

[B12] Neumann M. King D. Beltagy I. Ammar W. (2019). “ScispaCy: fast and robust models for biomedical natural language processing,” in Proceedings of the 18th BioNLP Workshop and Shared Task (Florence: Association for Computational Linguistics), 319–327. 10.18653/v1/W19-5034

[B13] Pandey H. G. Amod A. Kumar S. (2024). “Advancing healthcare automation: multi-agent system for medical necessity justification,” in Proceedings of the 23rd Workshop on Biomedical Language Processing (BioNLP 2024) (Bangkok: Association for Computational Linguistics), 39–49. 10.18653/v1/2024.bionlp-1.4

[B14] Plaat A. (2022). “Multi-agent reinforcement learning,” in Deep Reinforcement Learning (Springer Singapore: Singapore), 219–262. 10.1007/978-981-19-0638-1_7

[B15] Ren A. Z. Dixit A. Bodrova A. Singh S. Tu S. Brown N.. (2023). Robots that ask for help: uncertainty alignment for large language model planners. arXiv preprint arXiv:2307.01928 [cs.RO]. Available online at: https://arxiv.org/abs/2307.01928

[B16] Review of Ophthalmology (2025). Journal homepage. Newtown Square, PA: Review of Ophthalmology; Jobson Medical Information LLC. Available online at: https://www.reviewofophthalmology.com/ (Accessed September 24, 2025).

[B17] Singh A. K. Keenaghan M. (2025). “Sturge-Weber syndrome,” in StatPearls [Internet] (Treasure Island, FL: StatPearls Publishing). Available online at: https://www.ncbi.nlm.nih.gov/books/NBK459163/29083797

[B18] Smith R. (2007). “An overview of the tesseract ocr engine,” in Proceedings of the Ninth International Conference on Document Analysis and Recognition (ICDAR) (IEEE: Curitiba, Brazil), 629–633. 10.1109/ICDAR.2007.4376991

[B19] Strafella C. Caputo V. Termine A. Fabrizio C. Ruffo P. Potenza S.. (2021). Genetic determinants highlight the existence of shared etiopathogenetic mechanisms characterizing age-related macular degeneration and neurodegenerative disorders. Front. Neurol. 12:626066. 10.3389/fneur.2021.626066 34135841 PMC8200556

[B20] Sun C. (2022). Association of sleep behaviour and pattern with the risk of glaucoma: a prospective cohort study in the uk biobank. BMJ Open. 12:e063676. 10.1136/bmjopen-2022-063676 36319053 PMC9644340

[B21] University of Iowa Department of Ophthalmology and Visual Sciences (2025). EyeRounds: Ophthalmology Cases. Available online at: https://eyerounds.org/cases.htm (Accessed September 24, 2025).

[B22] Yiu G. (2025). Using natural language processing to score age-related macular degeneration severity. Ophthalmology 132, 1088–1090. 10.1016/j.ophtha.2025.06.012 40975596

[B23] Zhang H. Zuo Z. Li Z. Ma L. (2024). Leak detection for natural gas gathering pipelines under corrupted data via assembling twin robust autoencoders. Process Saf. Environ. Prot. 188, 492–513. 10.1016/j.psep.2024.05.112

[B24] Zhao B. Li Y. Fan Z. Wu Z. Shu J. Yang X.. (2024). Eye–brain connections revealed by multimodal retinal and brain imaging genetics. Nat. Commun. 15:6064. 10.1038/s41467-024-50309-w 39025851 PMC11258354

[B25] Zuo Z. Zhang H. Li Z. Ma L. Liang S. Liu T.. (2024). A self-supervised leak detection method for natural gas gathering pipelines considering unlabeled multi-class non-leak data. Comput. Ind. 159–160:104102. 10.1016/j.compind.2024.104102

[B26] Zuo Z. Zhang H. Ma L. Liu T. Liang S. (2023). Leak detection for natural gas gathering pipelines under multiple operating conditions using rp-1dconvlstm-ae and multimodel decision. IEEE Trans. Ind. Electron. 71, 6263–6273. 10.1109/TIE.2023.3294645

