# Supplementary material for: LLM-based multi-agent system for neuro-ophthalmic diagnosis and personalized treatment planning
Source: Front Neurosci. 2025 Oct 6;19:1688509. doi: 10.3389/fnins.2025.1688509 (PMC12536030; doi:10.3389/fnins.2025.1688509)
Supplement: Supplementary file 1 [file Table_1.docx]

**#### Disease Name: ####**

**Diabetic Retinopathy (DR) and Proliferative Diabetic Retinopathy (PDR)**

#### Disease Description: ####

Diabetic Retinopathy (DR) is the most common specific microvascular complication in patients with diabetes mellitus (DM). DR gradually progresses in people with DM from the mild non-proliferative DR (NPDR) stage to more severe vision-threatening stages including proliferative DR (PDR) and diabetic macular edema (DME). The pathogenesis of DR involves multiple pathological pathways associated with hyperglycemia including genetic and epigenetic factors, free radicals, advanced glycation end products, inflammatory factors and vascular endothelial growth factor (VEGF).

#### Patient Information: ####

##### Patient Age: #####

Unknown

##### Medical History: #####

Diabetes Mellitus (DM), Diabetic Retinopathy (DR), possibly with proliferative diabetic retinopathy (PDR) and diabetic macular edema (DME).

##### Current Medications: #####

Unknown

#### Condition of the Disease: ####

##### Lesion Location and Size: #####

The lesions are located in the retina, characterized by various forms such as microaneurysms, intraretinal hemorrhages, venous beading, intraretinal microvascular abnormalities, and hard exudates.

The size involves the central subfield zone in the case of DME, affecting a 1 mm diameter area around the fovea.

#### Indications: ####

All patients with diabetes, especially those with a history of diabetes and poor glycemic control.

Patients with fundus examination showing microvascular changes, including microaneurysms, retinal hemorrhages, hard exudates, cotton wool spots, and neovascularization.

#### Treatment: ####

##### Method 1: Systemic Medical Control #####

Systemic medical control is crucial for all DR patients, including maintaining hemoglobin A1c (HbA1c) levels below 7.0%, treating systemic hypertension, and managing dyslipidemia.

##### Method 2: Laser Photocoagulation (PRP) #####

Laser PRP is the primary treatment for PDR and may be considered for some high-risk severe NPDR patients. These include patients with poor follow-up, those undergoing cataract surgery, pregnant patients, and patients with one blind eye or progressive DR in the other eye. Modern PRP uses short-pulse lasers of 20 to 30 milliseconds, applying 2000 to 4000 treatment spots depending on the severity of PDR.

##### Method 3: Anti-VEGF Injections #####

Clinical trial evidence suggests that anti-VEGF injections are a safe and effective treatment for PDR, with sustained effects lasting up to two years. This therapy has been proven effective for ranibizumab (Lucentis) and aflibercept (Eylea), with bevacizumab (Avastin) also effective for retinal neovascularization. In resource-limited settings, PRP is generally recommended as the primary treatment for severe NPDR and all PDR stages.

#### Precautions: ####

Intraoperative Considerations: For PDR treatment, if PRP is used, choose appropriate laser parameters based on the severity of PDR.

Postoperative Management: Patients need regular follow-up to monitor vision recovery and macular edema progression. Treatment plans should be adjusted based on vision and OCT results.

**#### Disease Name: ####**

**Diabetic Macular Edema (DME)**

#### Disease Description: ####

Diabetic Retinopathy (DR) is the most common specific microvascular complication in patients with diabetes mellitus (DM). DR gradually progresses in people with DM from the mild non-proliferative DR (NPDR) stage to more severe vision-threatening stages including proliferative DR (PDR) and diabetic macular edema (DME). The pathogenesis of DR involves multiple pathological pathways associated with hyperglycemia including genetic and epigenetic factors, free radicals, advanced glycation end products, inflammatory factors and vascular endothelial growth factor (VEGF).

#### Patient Information: ####

##### Patient Age: #####

Unknown

##### Medical History: #####

Diabetes Mellitus (DM), Diabetic Retinopathy (DR), possibly with proliferative diabetic retinopathy (PDR) and diabetic macular edema (DME).

##### Current Medications: #####

Unknown

#### Condition of the Disease: ####

##### Lesion Location and Size: #####

The lesions are located in the retina, characterized by various forms such as microaneurysms, intraretinal hemorrhages, venous beading, intraretinal microvascular abnormalities, and hard exudates.

The size involves the central subfield zone in the case of DME, affecting a 1 mm diameter area around the fovea.

#### Indications: ####

All patients with diabetes, especially those with a history of diabetes and poor glycemic control.

Patients with fundus examination showing microvascular changes, including microaneurysms, retinal hemorrhages, hard exudates, cotton wool spots, and neovascularization.

#### Treatment: ####

##### Method 1: Focal or Grid Laser Photocoagulation #####

Focal or grid laser photocoagulation is considered an effective treatment for clinically significant macular edema. The modified Early Treatment Diabetic Retinopathy Study (ETDRS) focal or grid laser photocoagulation protocol is typically recommended for treating clinically significant macular edema.

##### Method 2: Anti-VEGF Therapy for Central Macular Edema #####

In recent years, intravitreal anti-VEGF injections have become the standard treatment to prevent vision loss in DME patients. In resource-rich countries, anti-VEGF treatment should be considered the first-line treatment for central macular edema in DME. For patients with good vision (6/9 or 20/30 or better) and central macular edema, three treatment options are available: anti-VEGF treatment only when DME worsens, intravitreal anti-VEGF injections, or combining focal or grid laser photocoagulation as needed. For patients with poorer vision (6/9 or 20/30 or worse), consider anti-VEGF treatment with ranibizumab 0.3 or 0.5 mg, bevacizumab 1.25 mg, or aflibercept 2 mg.

##### Method 3: Laser and Steroid Therapy #####

For eyes with persistent retinal thickening after anti-VEGF treatment, consider laser treatment after 24 weeks. Intravitreal triamcinolone (2 mg/0.05 ml or 4 mg/0.1 ml) may also be considered, especially in eyes with a previously implanted intraocular lens. In cases of DME with PDR, consider single anti-VEGF treatment, with reassessment for PRP or continued anti-VEGF treatment as needed.

#### Precautions: ####

Intraoperative Considerations: For PDR treatment, if PRP is used, choose appropriate laser parameters based on the severity of PDR.

Postoperative Management: Patients need regular follow-up to monitor vision recovery and macular edema progression. Treatment plans should be adjusted based on vision and OCT results.

**#### Disease Name: ####**

**Chronic Open Angle Glaucoma (COAG) and Ocular Hypertension (OHT)**

#### Disease Description: ####

Chronic Open Angle Glaucoma (COAG) is the most common type of glaucoma, usually progressing slowly with almost no symptoms in the early stages. Ocular Hypertension (OHT) refers to elevated intraocular pressure without signs of optic nerve damage or visual field defects.

#### Patient Information: ####

##### Patient Age: #####

Unknown

##### Medical History: #####

The patient has a history of glaucoma, possibly chronic open-angle glaucoma (COAG) as well as ocular hypertension (OHT).

The patient may have undergone various diagnostic tests including intraocular pressure measurement, central corneal thickness (CCT) assessment, and optic nerve head imaging.

##### Current Medications: #####

Unknown

#### Condition of the Disease: ####

##### Lesion Location and Size: #####

The lesion is related to the optic nerve head and the anterior chamber of the eye.

#### Indications: ####

Patients diagnosed with COAG or OHT.

Patients with intraocular pressure above 24 mmHg and at risk of vision loss.

#### Treatment: ####

##### Method 1: Initial SLT Treatment #####

For newly diagnosed OHT patients with intraocular pressure of 24 mmHg or above, 360° Selective Laser Trabeculoplasty (SLT) is recommended.

Patients should be informed that 360° SLT can delay the need for eye drops, but they may still require eye drops.

Patients should be informed about the time required for intraocular pressure improvement after the procedure and the specific side effects (e.g., temporary discomfort, blurred vision, photophobia, and conjunctival hyperemia) and their duration.

A second 360° SLT may be required later.

##### Method 2: Medication Management #####

For patients with COAG who are unsuitable or unwilling to undergo SLT, Prostaglandin Analog (PGA) eye drops may be considered as the first choice.

For patients already on PGA eye drops but with poor efficacy, consider switching to another PGA or using β-blockers, carbonic anhydrase inhibitors, sympathomimetic drugs, miotics, or combination therapy.

If the current treatment is not well tolerated, the first alternative should be another PGA.

If intolerable, consider β-blockers or non-generic PGAs.

If none are suitable, consider carbonic anhydrase inhibitors, sympathomimetic drugs, miotics, or combination therapy.

#### Precautions: ####

Preoperative Evaluation: Comprehensive assessment of risk factors, including intraocular pressure levels, Central Corneal Thickness (CCT), and optic nerve damage status, is necessary before choosing surgery or treatment.

Postoperative Management: Regular follow-up after surgery is needed to monitor intraocular pressure changes and optic nerve health, with treatment plans adjusted as necessary.

**#### Disease Name: ####**

**Self-sealing Posterior Scleral Perforation**

#### Disease Description: ####

A 58-year-old male patient sustained an injury from an airgun pellet. CT scans revealed a pellet lodged within the orbit, but no evidence of a penetrating ocular injury was found. The patient's right eye exhibited severe vitreous hemorrhage, accompanied by lens subluxation and traumatic cataract. During vitrectomy, a fibrin clot tightly adhered to the retinal surface, which was considered the self-sealing point of impact from the pellet.

#### Patient Information: ####

##### Patient Age: #####

58 years old

##### Medical History: #####

The patient was a victim of airgun aggression, resulting in multiple wounds to the chest, abdomen, limbs, groin, and face.

The patient presented with significant ocular trauma, including a full-thickness wound in the right upper eyelid, swelling, ecchymosis in the right orbital region, and a massive vitreous hemorrhage in the right eye.

CT scans revealed the presence of a lead pellet within the orbital cavity close to the optic nerve.

##### Current Medications: #####

Unknown

#### Condition of the Disease: ####

##### Lesion Location and Size: #####

The lesion involves a lead pellet located within the orbital cavity, superotemporally close to the optic nerve in the deep retrobulbar space, between the optic nerve and the lateral rectus muscle.

The vitreous cavity had significant blood thickening and clots, particularly in the central superonasal sector, with a fibrin mass adhering to the retinal plane.

#### Indications: ####

Patients who have sustained injuries from airgun or similar pellets, particularly when CT shows an intraorbital foreign body without an evident penetrating wound.

Patients experiencing significant visual deterioration accompanied by vitreous hemorrhage and other intraocular complications.

#### Treatment: ####

##### Method 1: Vitrectomy #####

Utilize a 25G vitrector to perform a 360-degree circumferential hyaloidectomy in the mid-peripheral region, followed by slow trimming of the posterior vitreous to induce posterior vitreous detachment.

During surgery, a fibrin clot tightly adherent to the retinal surface was observed and partially removed to prevent retinal perforation. Argon laser photocoagulation was applied to stabilize and delimit the traumatic area.

Phacoemulsification was simultaneously performed for cataract removal, and a three-piece intraocular lens (IOL) was implanted.

#### Precautions: ####

Special care should be taken when handling the fibrin clot to avoid unnecessary manipulation, which could lead to accidental perforation.

Regular follow-up is required to monitor visual recovery, with particular attention to retinal integrity and intraocular pressure changes.

**#### Disease Name: ####**

**Macular Hole**

#### Disease Description: ####

A 70-year-old female patient with pseudophakia presented with an acute central macular hole in the right eye. Initial examination revealed visual acuity of 20/25 in the right eye and 20/300 in the left eye. OCT showed a small defect in the inner limiting membrane (ILM) at the macular center, with vitreomacular separation.

#### Patient Information: ####

##### Patient Age: #####

60 years old

##### Medical History: #####

The first patient has a history of non-arteritic ischemic optic neuropathy in the left eye, occurring two years prior to the current condition, which followed cataract surgery.

The second patient presented with an acute central xanthic scotoma and decreased vision in the right eye for two days.

The thrid patient presented with decreased vision in the right eye for two weeks. There is no detailed medical history provided beyond this condition. The patient has a history of macular drusen and presented with a decrease in vision in the right eye for three months.

##### Current Medications: #####

Unknown

#### Condition of the Disease: ####

##### Lesion Location and Size: #####

For the first patient, a small defect in the internal limiting membrane (ILM) at the center of the fovea was identified in the right eye, progressing to a full-thickness macular hole.

For the second patient, a focal attachment of the hyaloid to the center of the fovea with a tiny defect involving the outer retinal layer in the right eye was observed, which later progressed to a full-thickness macular hole.

For the third patient, a full-thickness stage 2 macular hole in the right eye with underlying drusen was identified.

#### Indications: ####

Patients with decreased vision in the macular area, especially those with acute onset.

OCT showing ILM defects or macular hole.

#### Treatment: ####

##### Method 1: Vitrectomy #####

Use a vitrector to perform vitrectomy, inducing posterior vitreous detachment (PVD), followed by gas tamponade with C3F8.

#### Precautions: ####

Avoid unnecessary membrane peeling during surgery to minimize retinal damage.

Regular follow-up is necessary to monitor visual recovery and macular hole closure.

Postoperative face-down positioning is recommended.

No additional membrane peeling is performed during surgery.

**#### Disease Name: ####**

**Aqueous Deficient Dry Eye (ADDE)**

#### Disease Description: ####

Dry eye disease is a multifactorial condition affecting the ocular surface, characterized by tear film instability. It commonly presents as ocular discomfort, burning sensation, foreign body sensation, and blurred vision. Dry eye can be classified into evaporative dry eye (EDE) and aqueous deficient dry eye (ADDE). This case series focuses on patients with ADDE, particularly those unresponsive to conventional conservative treatments.

#### Patient Information: ####

##### Patient Age: #####

61 years old

##### Medical History: #####

Sjögren’ s syndrome (SS) diagnosed by rheumatologist with anti-SSA/Ro antibody test and labial gland biopsy.

Hypertension: 30.0% of patients (12 out of 40).

Diabetes: 25.0% of patients (10 out of 40).

Use of hormone therapy, anti-depressants, chemotherapy, prostaglandin analogue, and history of laser refractive surgery in some patients.

##### Current Medications: #####

1% prednisolone eyedrops

Cyclosporine eyedrops (0.05% or 0.1%)

#### Condition of the Disease: ####

##### Lesion Location and Size: #####

The lesions are located in the lacrimal punctum and involve canalicular mucosa.

The procedure affects 1-2 mm of the punctal mucosa, aiming for coagulation without extensive occlusion.

#### Indications: ####

Patients with aqueous deficient dry eye, especially those unresponsive to conventional eyedrop treatments.

Reduced tear secretion, with Schirmer test results less than 5.0mm.

#### Treatment: ####

##### Method 1: High-Frequency Radiowave Punctal Occlusion #####

The procedure is performed under local anesthesia using 0.5% Proparacaine eye drops.

Insert the electrode tip of the radiowave device (OcuRF®, Ilooda, South Korea) into the lower punctum, apply radiowaves (0.6-0.8 watts, 2 MHz) for 1-2 seconds to coagulate the punctal mucosa. Each punctum is treated 2-3 times to expand the coagulation area.

During coagulation, careful handling is required to avoid excessive coagulation of the punctal surface, which could lead to scarring. Apply a cold compress immediately postoperatively to reduce swelling.

#### Precautions: ####

Avoid excessive coagulation of the punctal surface to minimize the risk of scarring and punctal malformation.

Follow up 6 weeks postoperatively to assess improvements in tear secretion and ocular surface indices, including Schirmer test, tear film break-up time (TBUT), and corneal staining score.

If symptoms do not improve significantly, repeat surgery may be necessary. Some patients underwent up to 7 surgeries within 3 years, with an average interval of 8 months.

Due to the variable nature of dry eye disease and tear duct anatomy, regular follow-up is necessary, with repeat procedures as needed.

**#### Disease Name: ####**

**Intermittent Exotropia**

#### Disease Description: ####

Intermittent exotropia is a common ocular misalignment characterized by the outward deviation of the eye, which may be more noticeable during periods of fatigue, illness, or concentration. Patients may complain of blurred vision, diplopia, or eye strain, particularly when focusing on a single object for extended periods.

#### Patient Information: ####

##### Patient Age: #####

32 years old

##### Medical History: #####

Amblyopia: Present in 3 out of 40 patients (7.5%)

Anisometropia: Present in 8 out of 40 patients (20%)

Refractive errors: Mean spherical equivalents of -2.3 D in both eyes

##### Current Medications: #####

Unknown

#### Condition of the Disease: ####

##### Lesion Location and Size: #####

Lesions are related to the extraocular muscles, specifically involving the lateral rectus and medial rectus muscles. The specific lesion sizes are are implied through the angles of deviation measured postoperatively (ranging from 18 to 70 prism diopters).

#### Indications: ####

Patients with intermittent exotropia, particularly those requiring surgical adjustment.

Patients with large-angle exotropia or uncertain surgical outcomes.

#### Treatment: ####

##### Method 1: Surgical Procedure #####

Surgery is performed under general anesthesia using the fornix incision technique. The surgical plan for each group is based on the measured maximum deviation angle. Procedures include bilateral lateral rectus recession, unilateral lateral rectus recession, and medial rectus resection, depending on the degree of exotropia and the presence of a dominant eye.

In adjustable suture lateral rectus recession (LR-Adj group), the lateral rectus muscle is first disinserted from its attachment, and a 6-0 Vicryl suture is used to secure it. The double-armed suture with spatula needles is passed vertically and parallel to the scleral attachment line. Vertical scleral sutures are placed at the superior and inferior edges of the attachment, then passed centrally to meet the other suture. After measuring the recession amount, the lateral rectus is hung back and secured with a single-loop bowtie knot to the scleral attachment. A 4-0 Vicryl support suture is placed below the bowtie knot to facilitate easy untying during adjustment. Additionally, a 6-0 Vicryl traction suture is placed on the scleral side near the attachment to provide sufficient exposure during adjustment. All sutures are placed under the conjunctiva, which is then moved to the fornix incision.

In adjustable suture medial rectus resection (MR-Adj group), a full-thickness locking suture and a half-thickness suture are placed along each muscle edge 2 mm posterior to the planned resection point. The shortened muscle is then hung 2 mm behind the insertion point and secured with a single-loop bowtie suture, providing space for adjustment surgery if needed. The recessed muscle is positioned with the initially planned surgical amount, while the shortened muscle is hung 2 mm.

#### Precautions: ####

Ensure equal muscle length on both sides during adjustment to minimize postoperative deviation risk.

Adjustment surgery is usually performed 24 hours postoperatively. After applying local anesthesia (Proparacaine eye drops), the single-loop bowtie suture is untied, and the muscle position is adjusted to ensure both sides have equal muscle length. The suture is then secured with a permanent square knot, and the traction and support sutures are removed. Conjunctival suturing is performed at the end.

Regular follow-up is conducted at 1 week, 1 month, 4 months, 10 months, and 18 months to assess changes in horizontal deviation and surgical success rates.

Regular follow-up is essential to monitor surgical outcomes and make further adjustments if necessary.

**#### Disease Name: ####**

**Posterior Polar Cataract**

#### Disease Description: ####

Posterior polar cataract is a congenital condition that can be either sporadic or familial. Sporadic cases are typically unilateral and associated with remnants of the tunica vasculosa lentis, an embryologic hyaloid structure that fails to regress. Familial cases are typically bilateral and follow an autosomal dominant pattern of inheritance. These cataracts often present in the first few months of life and can lead to amblyopia if visually significant at an early age. They can progress in severity over time.

#### Patient Information: ####

##### Patient Age: #####

38 years old

##### Medical History: #####

No chronic medical conditions, including no diabetes or history of steroid use.

Past ocular history includes wearing glasses with no history of amblyopia.

##### Current Medications: #####

None

#### Condition of the Disease: ####

##### Lesion Location and Size: #####

Right eye (OD): Central 2.5 mm opacity in the posterior aspect of the lens.

Left eye (OS): Central 3.0 mm opacity in the posterior aspect of the lens with surrounding posterior subcapsular cataract and trace anterior subcapsular cataract.

#### Indications: ####

Abnormal red reflex

Amblyopia

Central posterior discoid plaque on the lens

Decreased vision

Glare

#### Treatment: ####

##### Method 1: Surgical Removal #####

Surgical removal is required when posterior polar cataracts become visually significant, either in infancy or adulthood. Due to the high risk of posterior capsular rupture, the surgery involves specific techniques:

Injection of Viscoelastic: Avoid injecting excessive viscoelastic into the anterior chamber as the increased anterior pressure can cause posterior capsular rupture.

Capsulorhexis: Avoid a large anterior capsulotomy. In the setting of a posterior capsular rupture, a large opening may not provide enough support for a sulcus intraocular lens.

Hydrodissection: Avoid hydrodissection as the fluid wave can cause rupture of the weak posterior capsule. Viscodissection can be safely utilized if the nucleus has been debulked.

Hydrodelineation: Inside-out delineation is a technique where a central bowl or trench is made in the anterior epinuclear material using low phacoemulsification settings. This technique avoids the inadvertent injection of fluid in the subcapsular plane, which can lead to posterior capsular rupture.

Lens Rotation: This step should be avoided.

Nucleofractis: Aspirate the nuclear material if the nucleus is soft, or use slow motion phacoemulsification (example: bottle height 50 cm, vacuum 100 mm Hg, and aspiration flow rate at 20 ml per minute) to gently remove nuclear fragments within the epinuclear shell.

Epinucleus Removal: Utilize the phacoemulsification probe and low settings to gently strip the epinucleus 360 degrees. The central epinuclear material and plaque are aspirated last. The vitrectomy cutter can also be used to remove the remaining epinucleus.

Polishing: Avoid polishing techniques as the posterior polar plaque is very adherent to the capsule, and polishing may lead to rupture of the capsule.

Lens Insertion: Use an AcrySof IOL in the bag if possible, even if there is a small posterior capsular rupture. In the setting of posterior capsular rupture, the anterior vitreous face can be tamponaded with a dispersive viscoelastic, and then the lens can be gently inserted into the bag.

#### Precautions: ####

Avoid excessive injection of viscoelastic into the anterior chamber to prevent posterior capsular rupture.

Avoid large anterior capsulotomy.

Do not perform irrigation and aspiration posterior to the intraocular lens (IOL).

**#### Disease Name: ####**

**Morgagnian Cataract**

#### Disease Description: ####

A Morgagnian cataract arises when a cortical cataract becomes hypermature. A cortical cataract is an opacity of the lens cortex or periphery of the lens. As the cataract matures, the lens fibers degenerate, leaving cytoplasmic protein globules between the fibers of the cortical lamellae. Increasing amounts of cortex degenerate with time, and the globules begin to coalesce, creating large accumulations of liquefied lens protein. When the majority of the cortex is affected, the lens nucleus is left floating in the liquid cortex, forming a Morgagnian cataract. Eyes with hypermature or mature cataracts may develop secondary elevated intraocular pressure referred to as phacolytic glaucoma. This occurs because of the increased concentration of protein molecules under the lens capsule, leading to water being drawn from the aqueous into the lens capsule via osmosis, resulting in a swollen, tense lens capsule. Lens proteins may leak from microscopic rents in the capsule, triggering a non-granulomatous inflammatory response. Macrophages collect in the anterior chamber and phagocytize the free lens proteins, which can clog the trabecular meshwork, hindering aqueous outflow and causing elevated intraocular pressure. With the increased tension on the lens capsule, there is an increased risk of spontaneous capsular rupture or rupture with only mild trauma. Exposure of the lens nucleus and cortical material to the anterior chamber after rupture can cause a severe granulomatous inflammatory reaction, known as phacoantigenic endophthalmitis. On histology, the lens material is surrounded by multinucleated giant cells, lymphocytes, and histiocytes in concentric layers.

#### Patient Information: ####

##### Patient Age: #####

59 years old

##### Medical History: #####

Hypertension

Type II Diabetes

Cerebral Vascular Accident

Past eye history: Six years ago, she had been told that she had a cataract in the right eye. No history of eye surgery or trauma.

##### Current Medications: #####

Aspirin

Carvedilol

Furosemide

Novolog

#### Condition of the Disease: ####

##### Lesion Location and Size: #####

Right eye (OD): White cataract with 4+ brunescent nuclear sclerosis, nucleus appeared sunken inferiorly in surrounding cortex.

Left eye (OS): 3+ nuclear sclerosis and diffuse cortical changes.

#### Indications: ####

Significant decreased vision affecting activities of daily living

Glare from bright lights at night

Difficulty with seeing road signs, watching television, and reading the newspaper

Dense cataracts causing poor vision

#### Treatment: ####

##### Method 1: Surgical Removal #####

Decreased vision caused by a Morgagnian or hypermature cataract can be significantly improved through surgical removal. However, creating a continuous curvilinear capsulorhexis (CCC) is more challenging due to increased intralenticular pressure, which can lead to uncontrolled tearing of the lens capsule. Additionally, the surgical view may be compromised by a lack of red reflex and the leakage of liquefied cortical material into the anterior chamber following the initial capsular opening. These challenges necessitate specialized techniques to ensure successful surgery, as outlined below:

Improving Visualization During Surgery: Operating Room Environment: Dimming the operating room lights, increasing microscope magnification, and enhancing coaxial illumination. Capsule Staining: Staining the anterior lens capsule with Trypan blue is often used to provide uniform capsular staining, enhancing contrast between the anterior capsule and the underlying lens. This improves visualization of the anterior edge of the capsulorhexis. Side Illumination: Using side illumination with an endoilluminator can improve visualization in cases with a compromised red reflex. The endoilluminator's advantage is its ability to be continuously repositioned to identify the advancing edge of the capsular tear.

Techniques to Decrease the Risk of Uncontrolled Capsular Tears: Two-Stage Capsulorhexis: A deliberately small continuous curvilinear capsulorhexis is created first. The liquefied lens contents are aspirated. The capsulorhexis is then secondarily enlarged. This technique has been shown to reduce the incidence of unexpected radial tears compared to the traditional one-stage 5 to 6 mm capsulorhexis technique. Capsular Decompression: An anterior capsular opening is first created with a needle, followed by intermittent aspiration of the liquid material to decompress the lens capsule. This decompression is followed by the completion of the capsulorhexis using endoillumination.

Alternative Techniques for Capsulorhexis Creation: Neodymium Laser: Using a neodymium laser to create an anterior capsulotomy just before surgery can help release liquefied cortical material into the anterior chamber, decreasing intralenticular pressure. Bipolar Diathermic Capsulotome: As an alternative to capsulorhexis, a bipolar diathermic capsulotome can be used to create a round anterior capsulotomy.

Nucleus Removal Techniques: Hydrodissection/Hydrodelineation: These are not necessary as the nucleus is already mobile within the capsule. Visco-Shell Technique: After the capsulorhexis, sodium hyaluronate 2.3% (Healon5) is injected gently into the capsular bag between the nucleus and the posterior capsule, and additional Healon5 is injected between the nucleus and the cornea. This technique stabilizes the nucleus for removal while protecting both the posterior capsule and the cornea. Phaco-Chop Technique: This technique may be successful for the removal of the nucleus. Divide and Conquer Technique: This technique is more difficult because the lens nucleus is floating freely in the capsular bag. Lens Snare: If the lens nucleus is particularly hard, a lens snare can be used to break the nucleus into small pieces prior to phacoemulsification. Alternatively, the entire small nucleus can be removed with a small incision extracapsular cataract extraction (ECCE).

#### Precautions: ####

The increased risk of posterior capsular rupture, endothelial cell damage, or nucleus dislocation into the vitreous must be communicated to the patient.

The use of techniques that reduce radial capsular tears is crucial to a successful surgical outcome.

Use of side illumination, staining, and two-stage capsulorhexis can improve visualization and control during surgery.

**#### Disease Name: ####**

**Diabetic White Cataract**

#### Disease Description: ####

Cataracts are a well-known consequence of untreated diabetes mellitus and are a common cause of visual impairment. Cataracts are 3-4 times more common in young diabetic patients compared to their non-diabetic counterparts. The development of diabetic cataracts is primarily influenced by poor glycemic control and the duration of the disease. In younger patients with early or mild cataracts, good glycemic control may allow for a reversal in cataract severity, though this is not typically the case for older adults or patients with mature cataracts.

The pathophysiology involves the activation of the polyol (sorbitol) pathway of intralenticular glucose metabolism in chronic hyperglycemia. In a non-diabetic environment, glucose in the aqueous humor is metabolized by the lens through anaerobic glycolysis. However, in hyperglycemic states, excess glucose is shunted towards the sorbitol pathway within the lens, where it is converted to sorbitol by aldose reductase and subsequently to fructose by polyol dehydrogenase. The accumulation of sorbitol and fructose increases osmotic pressure within the lens, causing lens fiber swelling and opacification, leading to the development of a white cataract. Longstanding hyperglycemia may result in the formation of a hypermature cataract, characterized by the degeneration of cortical material into a hyperosmotic liquid, which creates a tense capsule with a free-floating nucleus.

#### Patient Information: ####

##### Patient Age: #####

21 years old

##### Medical History: #####

Type 1 diabetes mellitus

Anxiety

Past ocular history: Myopia, corrective lenses since age 13; no prior eye exam; denies history of amblyopia or patching; denies prior ocular surgeries or trauma.

##### Current Medications: #####

Insulin detemir U-100, 35 units twice daily

Insulin regular, sliding scale with meals

Hydroxyzine, 50 mg q6 as needed

Mirtazapine, 30 mg daily

Sertraline, 150 mg daily

#### Condition of the Disease: ####

##### Lesion Location and Size: #####

Dense white cataract in both eyes (OU) related to diabetes.

#### Indications: ####

Dense, white cataract

Decreased visual acuity, particularly during hyperglycemic events

Myopic shift

Absent red reflex

Glare and reduced vision in low light or at night

#### Treatment: ####

##### Method 1: Visualization Enhancements #####

The lack of red reflex in white cataracts complicates the visualization of the anterior lens capsule. Use of trypan blue to stain the anterior capsule is recommended to enhance visualization during capsulorhexis formation.

Staining with trypan blue or indocyanine green has been documented as a safe and effective method to improve intraoperative visualization of the anterior capsule.

##### Method 2: Capsulorhexis Technique #####

A central incision in the capsule with a 27-gauge needle can facilitate controlled entry into the anterior capsule and decompress the capsular bag.

If liquefied material exits the capsule, immediate aspiration and gentle depression of the lens are recommended to decompress the capsular bag and reduce the risk of extension under pressure.

In cases where the capsulorhexis radializes, the “Little technique” can be applied. After filling the anterior chamber with an ophthalmic viscosurgical device (OVD) and lying the capsule flap flat against the cortex, tractional force applied to the free capsule flap should be reversed to redirect centrally, allowing the tear to advance along the circumferential path.

##### Method 3: Phacoemulsification #####

In dense cataracts, using techniques like phaco-chop or miLoop is recommended to limit the use of excessive phacoemulsification energy. These techniques help reduce the risk of zonulopathy, which occurs more frequently with higher density white cataracts.

#### Precautions: ####

Capsular Stability: Capsular tension hooks or capsular tension rings may be warranted to provide adequate stability of the capsular bag during surgery. In cases of parasympathetic neuropathy, which may hinder adequate pupillary dilation, a Malyugin ring or iris hooks might be necessary to maintain visualization and access to the lens capsule.

The swollen lenticular material created by the osmotic shifts of hyperglycemia can cause the capsule to become tense and pressurized, requiring careful management during surgery.

Special attention must be paid to the initiation and progression of the capsulorhexis to avoid uncontrolled radial splitting, which can lead to complications such as the "Argentinian flag sign."

Proper use of capsular tension devices and visualization aids is crucial in managing these complex cases.

**#### Disease Name: ####**

**Aphakic Glaucoma**

#### Disease Description: ####

Aphakic glaucoma is classified as a secondary form of open-angle glaucoma and is the second most common cause of glaucoma in the pediatric population. The development of aphakic glaucoma has been associated with several factors, including surgery within the first year of life, corneal diameter less than 10 mm, retained lens proteins, the presence of other ocular abnormalities, certain cataract types (e.g., complete, nuclear, or persistent hyperplastic vitreous), and a history of secondary surgeries. The most consistent risk factor across the literature is cataract surgery at a young age. Early lensectomy is thought to interfere with the maturation of the trabecular meshwork, which is essential for proper intraocular pressure (IOP) regulation. Other contributing factors include postoperative inflammation, retained lens material, and possibly a genetic predisposition. Aphakic glaucoma typically develops several years after cataract surgery, with a significant percentage of patients affected within a decade following surgery.

#### Patient Information: ####

##### Patient Age: #####

5 years old

##### Medical History: #####

No significant past medical history.

Past ocular history: Congenital cataract OS, status post extracapsular cataract extraction at 7 months of age. Diagnosed with amblyopia OS and underwent patching of the right eye (OD) for 3 hours per day. Sensory exotropia OS.

##### Current Medications: #####

Timolol 0.25%, 1 drop, twice a day, OS

#### Condition of the Disease: ####

##### Lesion Location and Size: #####

Left eye (OS): Significant buphthalmos, upper and lower eyelid retraction, clear cornea with contact lens in place, deep and quiet anterior chamber, superotemporal peripheral iridectomy, aphakic lens status. Thinning of the neuroretinal rim, cup-to-disc ratio of 0.7, generalized constriction of the visual field with only a small paracentral island remaining.

#### Indications: ####

Elevated intraocular pressure (IOP)

Corneal clouding

Excessive loss of hyperopia

Thick corneas and small anterior segments

Open-angle with possible trabecular meshwork obstruction by pigment or crystalline deposits

Optic nerve cupping

Visual field constriction

Thinning of the retinal nerve fiber layer on ocular coherence tomography (OCT) of the optic nerve

#### Treatment: ####

##### Method 1: Medical Management #####

Topical IOP-Lowering Medications: Initial treatment typically involves the use of topical medications to lower IOP. These include beta-blockers, carbonic anhydrase inhibitors, prostaglandin analogs, and alpha agonists.

##### Method 2: Surgical Management #####

Glaucoma Drainage Devices: If medical management is insufficient to control IOP, surgical intervention is necessary. Placement of a glaucoma drainage device is often required, particularly in cases where thick corneas and small anterior segments make other surgical techniques challenging. A pars plana vitrectomy may be performed to facilitate the placement of the drainage tube behind the iris, reducing the risk of complications associated with tube placement in the anterior chamber. Complications of drainage device surgery include malpositioning or migration of the tube, endophthalmitis, and corneal decompensation.

Trabeculectomy: Trabeculectomy is another surgical option, although its use may be complicated in patients who require aphakic contact lenses, as these can interfere with the function of the bleb created during surgery. This procedure involves creating a drainage pathway for aqueous humor to lower IOP, but it may require subsequent interventions due to the challenging anatomy of pediatric aphakic patients.

#### Precautions: ####

Postoperative Management: Close monitoring and long-term follow-up are crucial due to the progressive nature of aphakic glaucoma and the potential need for additional surgical interventions. Postoperative management includes regular assessment of IOP, optic nerve head evaluation, and visual field testing.

The need for early and consistent monitoring of IOP post-cataract surgery in pediatric patients is paramount, as aphakic glaucoma can develop asymptomatically.

Regular screening every 3 months in the first postoperative year, biannually for the next 10 years, and annually thereafter is recommended to ensure early detection and treatment.

**#### Disease Name: ####**

**Neovascular Glaucoma (NVG)**

#### Disease Description: ####

Neovascular glaucoma (NVG) is a severe form of secondary angle closure glaucoma (ACG) that develops as a result of retinal ischemia, leading to increased production of vascular endothelial growth factor (VEGF) and subsequent neovascularization of the iris (NVI) and the iridocorneal angle. NVG is often associated with ischemic retinal conditions, such as central retinal vein occlusion (CRVO), proliferative diabetic retinopathy (PDR), and ocular ischemic syndrome (OIS). The condition is divided into four stages based on clinical and gonioscopic findings: prerubeosis, preglaucoma, open-angle glaucoma, and angle closure glaucoma. NVG is characterized by high intraocular pressure (IOP) and poor visual prognosis.

#### Patient Information: ####

##### Patient Age: #####

80 years old

##### Medical History: #####

Atrial fibrillation

Hypertension

Hyperlipidemia

Past ocular history: Early primary open angle glaucoma (POAG) treated for seventeen years with latanoprost every night at bedtime in both eyes (OU) and timolol every morning OU, with stable visual fields OU.

##### Current Medications: #####

Warfarin

Aspirin

Timolol daily OU

Latanoprost every night at bedtime OU

#### Condition of the Disease: ####

##### Lesion Location and Size: #####

Right eye (OD): Central retinal vein occlusion (CRVO) with severe cystoid macular edema (CME) and numerous dot-blot hemorrhages (DBH) and flame hemorrhages throughout the macula and mid-periphery. Engorged iris vessels, severe cystoid macula edema with a central macula thickness of 981 microns. Severe optic nerve damage with cup-to-disc ratio of 0.85. Delayed arteriolar filling and diffuse late leakage in the macula on fluorescein angiography. Constriction of the I2e isopter and nasal step of the I4e isopter on Goldmann visual fields.

#### Indications: ####

Neovascularization of the iris and iridocorneal angle

Elevated intraocular pressure (IOP)

Elevated cup-to-disc ratio

Relative afferent pupillary defect (RAPD)

Hyphema (blood in the anterior chamber)

Conjunctival congestion

Corneal edema

#### Treatment: ####

##### Method 1: Medical Management #####

Topical Aqueous Suppressants: Medications aimed at decreasing aqueous humor production, such as beta-blockers, carbonic anhydrase inhibitors, and alpha agonists, are typically used to lower IOP in NVG patients.

Topical Corticosteroids: Corticosteroids may be employed to reduce inflammation and improve aqueous humor outflow. However, prostaglandin analogs should be avoided as they may further compromise the blood-aqueous barrier and exacerbate NVG.

Avoidance of Pilocarpine and Anticholinergics: These medications are generally avoided because they can increase inflammation, induce miosis, worsen synechial angle closure, and reduce uveoscleral outflow.

##### Method 2: Surgical Management #####

Panretinal Photocoagulation (PRP): PRP is a laser treatment used to reduce neovascularization by decreasing the overall oxygen demand of the retina. This procedure aims to balance angiogenic and antiangiogenic factors, thereby stabilizing the disease process. PRP is particularly important in cases of retinal ischemia-induced NVG, such as in PDR or ischemic CRVO. PRP alone may take several weeks to reduce neovascularization and is often supplemented with anti-VEGF therapy for faster results.

Anti-VEGF Intravitreal Injections: Intravitreal injections of anti-VEGF agents, such as bevacizumab, target the primary pathway of neovascularization. Anti-VEGF therapy is particularly effective when combined with PRP, improving regression rates of NVI and NVA and reducing the risk of hyphema and other complications.

Glaucoma Drainage Devices: Placement of a glaucoma drainage device, such as an Ahmed valve, is often required in cases where medical management and laser treatments are insufficient to control IOP. These devices help to drain aqueous humor from the eye, lowering IOP and reducing the risk of further glaucomatous damage. In cases of significant inflammation or active hyphema, an aqueous shunt is recommended, with the tube potentially placed behind the iris to protect it from NVG-associated fibrovascular membranes.

Cyclophotocoagulation (CPC): CPC is a procedure that uses laser energy to ablate part of the ciliary body, reducing aqueous humor production. This treatment is considered when other surgical options fail to control IOP.

Trabeculectomy: Trabeculectomy is a surgical procedure aimed at creating a drainage pathway for aqueous humor. While it can achieve lower postoperative IOP, it is less effective in NVG compared to other types of glaucoma and carries a higher risk of complications such as hyphema.

#### Precautions: ####

Postoperative Management: Regular follow-up is essential to monitor IOP, optic nerve health, and visual field status. Additional PRP sessions or anti-VEGF injections may be needed if neovascularization persists or recurs.

Early and regular monitoring of high-risk patients, especially within the first six to eight months after diagnosis of ischemic CRVO or PDR, is crucial to detect and manage NVG promptly.

Visual acuity and the extent of retinal non-perfusion are important predictors of NVG risk and should guide treatment decisions.

Special attention should be given to potential complications, such as synechial angle closure, hyphema, and corneal edema, which can significantly affect prognosis and treatment outcomes.

**#### Disease Name: ####**

**Birdshot Choroiditis (also known as Vitiliginous Chorioretinitis)**

#### Disease Description: ####

Birdshot choroiditis is a rare, chronic, bilateral, inflammatory condition that affects the choroid and retina. It typically presents in healthy Caucasian women in their late 40s to 50s. The disease is characterized by blurred vision, floaters, photopsias, and progressive visual impairment. The hallmark of the condition is the presence of distinctive, multifocal, cream-colored, depigmented spots at the level of the retinal pigment epithelium (RPE) and choroid. These lesions often extend radially from the optic nerve, primarily affecting the posterior pole and nasal retina. The condition is strongly associated with the HLA-A29 antigen, which is found in the majority of affected individuals.

#### Patient Information: ####

##### Patient Age: #####

55 years old

##### Medical History: #####

Hypertension, first-degree AV block with a right bundle branch block.

Recent orthopedic procedure for the left foot.

Past ocular history: High myopia (approximately -10.00 D OU).

##### Current Medications: #####

None

#### Condition of the Disease: ####

##### Lesion Location and Size: #####

Both eyes (OU): Hazy vision with diffuse pigmented cells in the vitreous, posterior vitreous detachment with vitreous opacities. Clumps of large pigmented cells and debris scattered diffusely through the vitreous. B-scan ultrasound confirmed posterior vitreous detachment with diffuse vitreous opacities. Macula OCT showed a small sub-foveal neurosensory detachment and epiretinal membrane (ERM) in the right eye (OD) and a mild ERM in the left eye (OS).

#### Indications: ####

A 55-year-old female presented with persistent vitreous floaters and hazy vision in both eyes, which began six months prior.

Initial symptoms included blurred vision, photopsias, and increased floaters, with a diagnosis of hemorrhagic vitreous detachment.

Progressive worsening of vision led to referral, with visual acuity reduced to 20/40 OD and 20/30 OS.

Physical examination revealed large pigmented cells and debris in the vitreous, posterior vitreous detachment, and diffuse vitreous opacities.

#### Treatment: ####

##### Method 1: Initial Corticosteroid Therapy #####

The patient was started on oral Prednisone 40 mg daily, which resulted in improved visual acuity (20/30+2 OD and 20/25+2 OS) and partial clearing of vitreous debris.

##### Method 2: HLA-A29 Antigen Testing #####

HLA-A29 testing was conducted and returned positive, confirming the diagnosis of Birdshot choroiditis.

##### Method 3: Management of Recurrence #####

When the vitritis recurred after tapering Prednisone to 15 mg daily, the patient was referred to a rheumatologist to initiate treatment with mycophenolate mofetil (CellCept), a steroid-sparing immunosuppressant.

##### Method 4: Immunosuppressant Therapy #####

Mycophenolate Mofetil (CellCept): Prescribed as a steroid-sparing agent to control inflammation.

Other Immunosuppressants: Depending on patient response and tolerance, alternatives may include cyclosporine (2.5-5 mg/kg/day), azathioprine (1.5-2 mg/kg/day), methotrexate, daclizumab, or intravenous immunoglobulin (IV-Ig).

Periocular Steroid Injections: These are particularly effective in managing CME and recurrences. If systemic steroids are required for more than 3-4 months, a second steroid-sparing agent should be added.

#### Precautions: ####

Slow tapering of oral steroids is crucial to prevent a relapse of vitritis and other inflammatory symptoms.

Long-term management often requires extended therapy with steroid-sparing agents to minimize the side effects associated with chronic steroid use.

Regular monitoring of visual acuity and fundus examination, along with OCT and ERG, is essential to assess disease progression and treatment efficacy.

**#### Disease Name: ####**

**Idiopathic Uveal Effusion Syndrome (Type 2)**

#### Disease Description: ####

Idiopathic Uveal Effusion Syndrome (UES) is a rare condition characterized by the accumulation of serous fluid in the choroidal space, leading to choroidal effusions and, in some cases, serous retinal detachments. The disease is often associated with scleral abnormalities that impair the outflow of protein and fluid from the choroidal circulation. It most commonly affects middle-aged, hyperopic men. UES is divided into three types based on clinical features and scleral histopathology, with Type 2 occurring in non-nanophthalmic eyes with clinically abnormal sclera but without excessive refractive error. The condition is idiopathic, meaning it arises spontaneously without a clear underlying cause.

#### Patient Information: ####

##### Patient Age: #####

74 years old

##### Medical History: #####

Type-II diabetes mellitus managed with oral medications

Essential hypertension

Peripheral vascular disease

Hearing loss

Coronary artery disease

Past ocular history: Pseudophakia OU

##### Current Medications: #####

Aspirin

Atenolol

Atorvastatin

Furosemide

Gabapentin

Glimepiride

Hydrochlorothiazide

Losartan

Metformin

Naproxen

Nitroglycerin

Repaglinide

Silver sulfadiazine

Triamcinolone

#### Condition of the Disease: ####

##### Lesion Location and Size: #####

Left eye (OS): Choroidal folds, subretinal fluid beneath the fovea and inferiorly. Large nasal serous choroidal effusion, superior choroidal effusion, temporal choroidal effusions to the equator, inferior cobblestone degeneration. Residual subfoveal subretinal fluid and shallow choroidal effusions after surgery, with diffuse “leopard-spot” pigmentation observed throughout the posterior pole OS.

#### Indications: ####

A 74-year-old male presented with new photopsias and temporal visual field loss in the left eye (OS).

Recent episode of episcleritis treated with topical prednisolone drops, followed by the onset of symptoms.

Visual acuity was 20/70+1 in the left eye, with a temporal visual field defect.

#### Treatment: ####

##### Method 1: Medical Management #####

The patient was initially treated with oral prednisone (40 mg daily), but due to a lack of significant improvement, surgical intervention was pursued.

Medical management with steroids (oral, topical, or periocular) is sometimes attempted before surgery, especially in cases without scleral abnormalities. However, the effectiveness can be variable.

##### Method 2: Surgical Treatment #####

Scleral Window Surgery: The patient underwent scleral window surgery in the left eye. This procedure involves creating full-thickness sclerotomies in each quadrant to allow drainage of the subretinal fluid and reduce scleral resistance. The sclera was noted to be thick during surgery, and pathology confirmed the presence of normal scleral tissue.

Postoperative Course: After surgery, the choroidal effusions gradually decreased, and the exudative retinal detachment resolved. The patient’s visual acuity improved to 20/40-2 five months post-surgery and eventually reached 20/20 eight months after surgery. However, residual subfoveal subretinal fluid and shallow choroidal effusions persisted, along with diffuse "leopard-spot" pigmentation throughout the posterior pole OS, indicative of chronic subretinal fluid.

#### Precautions: ####

Surgical intervention is indicated when there is subretinal fluid involving or threatening the fovea, or when medical management is ineffective.

Postoperative follow-up is essential to monitor for the resolution of effusions and the potential recurrence of subretinal fluid.

Patients should be educated about the relapsing-remitting nature of the disease and the possibility of requiring further surgical intervention if recurrences occur.

**#### Disease Name: ####**

**Uveal Lymphoma (Extranodal Marginal Zone B-cell Lymphoma)**

#### Disease Description: ####

Uveal Lymphoma is a rare form of intraocular lymphoma, typically of B-cell origin, most commonly presenting as extranodal marginal zone lymphoma (MALT type). It primarily affects the choroid but can also involve the ciliary body and iris. The disease is often indolent, with slow progression, but can masquerade as other ocular diseases, leading to delays in diagnosis. The exact etiology remains unclear, though associations with autoimmune diseases and chronic infections have been suggested. Uveal lymphomas can be primary or secondary manifestations of systemic lymphoma.

#### Patient Information: ####

##### Patient Age: #####

71 years old

##### Medical History: #####

Hepatocellular carcinoma

Type 2 diabetes mellitus with mild non-proliferative retinopathy OS

Chronic kidney disease

Hyperlipidemia

Essential hypertension

Open-angle glaucoma OU treated with bimatoprost

Bilateral asymmetric sensorineural hearing loss

Vitamin B12 deficiency anemia

Depressive episodes

##### Current Medications: #####

Allopurinol

Amlodipine

Citalopram

Cyanocobalamin

Empagliflozin (Jardiance)

Lamotrigine

Bimatoprost (Lumigan®)

Metformin

Metoprolol succinate

Pravastatin

Insulin degludec and liraglutide (Xultophy®)

#### Condition of the Disease: ####

##### Lesion Location and Size: #####

Left eye (OS): Subconjunctival salmon-colored, elevated infiltrate from 6:00 to 12:00 extending to the limbus with associated 1+ injection. B-scan ocular ultrasound showed extensive choroidal infiltrate throughout the posterior pole most elevated in the macula (2.1 mm). OCT macula showed diffuse “lumpy-bumpy” replacement and thickening of the choroid with thin overlying subretinal fluid extending beneath the fovea.

MRI images demonstrated contrast enhancement, diffusion restriction, and near circumferential, asymmetric thickening of the wall of the left globe most prominent along the posterior lateral aspect with involvement of the adjacent optic nerve sheath.

#### Indications: ####

A 71-year-old male presented with chronic blurred vision in the left eye (OS) without acute changes.

Past medical history includes open-angle glaucoma, type 2 diabetes mellitus, and hepatocellular carcinoma.

Examination revealed a salmon-colored subconjunctival infiltrate in the left eye and subretinal fluid in the macula.

Imaging and biopsy confirmed the diagnosis of extranodal marginal zone B-cell lymphoma of the left eye with choroidal, episcleral, and orbital involvement.

#### Treatment: ####

##### Method 1: Radiation Therapy #####

The patient was treated with external beam radiation therapy (EBRT) to the left eye. A total of 12 fractions (24 Gy) of radiation were administered, which resulted in the resolution of the subconjunctival infiltrate.

EBRT is the most commonly reported treatment for localized uveal lymphoma, typically leading to full remission. The dosage usually ranges from 28 to 40 Gy, depending on the grade of lymphoma.

##### Method 2: Chemotherapy #####

In cases with systemic involvement, chemotherapy, often combined with rituximab (a monoclonal anti-CD20 antibody), is utilized. However, in this patient, chemotherapy was used for the management of a concurrent bladder cancer, not for the uveal lymphoma.

Rituximab is generally well-tolerated, with side effects that are less severe compared to those of radiation therapy.

#### Precautions: ####

Regular follow-up is essential to monitor for recurrence or progression of the disease.

Side effects of radiation therapy, such as cataract formation, dry eye syndrome, neovascular glaucoma, and radiation retinopathy, should be monitored.

In cases involving systemic lymphoma, coordination with an oncologist is necessary for comprehensive care and staging.

**#### Disease Name: ####**

**ANCA-associated Granulomatous Vasculitis (Wegener's Granulomatosis)**

#### Disease Description: ####

ANCA-associated granulomatous vasculitis, also known as Wegener's Granulomatosis, is a rare granulomatous necrotizing vasculitis that affects small to medium-sized vessels, primarily in the lungs and kidneys but can also involve other organs. The disease is characterized by a combination of granulomatous inflammation and vasculitis. Ocular involvement is common, with up to 50% of systemic cases showing ocular or orbital manifestations, although isolated orbital involvement is rare. The diagnosis is often delayed due to the non-specific nature of the initial symptoms.

#### Patient Information: ####

##### Patient Age: #####

54 years old

##### Medical History: #####

Deviated septum

Nasal polyps

Hernia repair

Tonsillectomy

Presbyopia

##### Current Medications: #####

Ciprofloxacin and dexamethasone otic (Ciprodex®) drops to ears

Refresh PM® (mineral oil/white petrolatum lubricant eye ointment)

#### Condition of the Disease: ####

##### Lesion Location and Size: #####

Right Eye (OD): Diplopia, "darkening of vision," visual acuity worsened to hand motion, mild temporal pallor, dense relative cecocentral scotoma, RAPD (1.2 log units).

Left Eye (OS): Post-operative proptosis, facial droop, optic disc edema, dilated episcleral veins, dot hemorrhages, chorioretinal scar inferotemporally, worsening visual acuity to 20/150, 3mm inferior scleral show, visual field deficit.

MRI brain with gadolinium showed a hyperintense lesion between the medial rectus and the lamina papyracea in the left orbit. Biopsy revealed sclerotic tissue with a scattered chronic granulomatous inflammatory infiltrate and focal areas of necrosis, leading to a diagnosis of ANCA-associated granulomatous vasculitis (Wegener's Granulomatosis).

#### Indications: ####

The patient, a 54-year-old male, presented with binocular vertical diplopia that worsened over four months.

He noticed "darkening" vision in the right eye (OD) while driving.

Subsequently developed proptosis and facial droop on the left side (OS), which was initially treated as Bell's palsy.

Despite treatment with steroids, his symptoms persisted and worsened, leading to further investigation.

#### Treatment: ####

##### Method 1: Initial Steroid Therapy #####

The patient was initially treated with high-dose steroids (methylprednisolone 1000 mg intravenously for 3 days, followed by an oral prednisone taper starting at 80 mg daily) under the presumption of idiopathic orbital inflammatory syndrome (IOIS). Although there was initial improvement, the lack of sustained response prompted further evaluation.

##### Method 2: Reevaluation and Biopsy #####

A transcaruncular anterior orbitotomy was performed, and a biopsy revealed chronic granulomatous inflammation with patchy necrosis, consistent with ANCA-associated granulomatous vasculitis.

A laboratory workup, including cANCA titers, supported the diagnosis.

##### Method 3: Cyclophosphamide and Steroids #####

The patient was referred to rheumatology and started on cyclophosphamide, with plans to transition to methotrexate once remission was achieved.

Corticosteroids were continued, with the dosage adjusted as needed.

##### Method 4: Plasmapheresis #####

In severe cases with end-organ damage, including pulmonary involvement, plasmapheresis may be considered.

#### Precautions: ####

Continuous monitoring is required to assess the response to treatment and adjust medications as necessary.

Side effects of cyclophosphamide and corticosteroids, including potential immunosuppression, should be managed carefully.

Multidisciplinary collaboration is crucial for accurate diagnosis and effective management.

**#### Disease Name: ####**

**Retinal Artery Macroaneurysm (RAMA)**

#### Disease Description: ####

Retinal Artery Macroaneurysm (RAMA) is an idiopathic, acquired dilation of a major retinal arteriole, typically developing within the first three bifurcations, at branch points, or areas of arteriovenous crossing. The superotemporal arteriole is the most common site for RAMAs. The condition is associated with systemic hypertension, arteriosclerosis, and sometimes other systemic conditions. RAMAs are often unilateral and can lead to vision loss due to hemorrhage or macular edema. The underlying pathophysiology may involve arteriosclerosis-induced vessel wall fibrosis, leading to decreased elasticity and aneurysmal dilation.

#### Patient Information: ####

##### Patient Age: #####

85 years old

##### Medical History: #####

Hypertension

Hypothyroidism

Osteopenia

Hypercholesteremia

Ocular hypertension

Pseudoexfoliation

Cataract surgery OD

Nuclear sclerotic cataract OS

##### Current Medications: #####

Timolol drops OU

Systemic medications include aspirin, levothyroxine, losartan, simvastatin, risedronate, tolterodine tartrate

#### Condition of the Disease: ####

##### Lesion Location and Size: #####

Right Eye (OD): Large area of subretinal hemorrhage extending into the macula, superotemporal retinal arteriole aneurysm, subretinal fluid under the fovea.

Left Eye (OS): Healthy optic nerve with cup-to-disc ratio of 0.3, normal vessels, macula, and periphery.

Fundus fluorescein angiography (FFA) showed an aneurysm along the superotemporal retinal arteriole with surrounding area of blockage from hemorrhage. Optical coherence tomography (OCT) revealed subretinal fluid under the fovea OD and extensive intraretinal edema with no choroidal abnormality.

#### Indications: ####

An 85-year-old female presented with sudden-onset darkening of her central vision in the right eye, surrounded by a gray ring-like pattern.

The patient had a history of ocular hypertension, pseudoexfoliation, and cataract surgery.

Examination revealed a large area of subretinal hemorrhage extending into the macula, with an aneurysm along the superotemporal retinal arteriole.

#### Treatment: ####

##### Method 1: Observation #####

The majority of RAMAs involute spontaneously without intervention, and patients often recover a significant amount of vision. Therefore, observation is the primary approach, especially if the aneurysm is not causing significant visual impairment.

Systemic risk factors such as hypertension should be managed appropriately.

##### Method 2: Laser Treatment #####

Indications for Laser Treatment: Laser photocoagulation may be considered in cases of vision loss due to chronic macular exudates, edema, or neurosensory detachments.

Types of Laser Treatment: Direct Photocoagulation: Directly targeting the macroaneurysm using xenon arc, argon, or dye yellow lasers has been documented to improve vision in some cases. However, it carries the risk of branch retinal artery occlusion and does not always lead to improved outcomes.

Indirect Laser Treatment: Laser treatment to the area surrounding the macroaneurysm may help in cases with macular edema.

Laser Hyaloidotomy: This technique using an Nd:YAG laser for subhyaloid hemorrhage is controversial due to potential risks such as vitreous hemorrhage or macular damage.

##### Method 3: Surgical Intervention #####

In cases of vitreous hemorrhage with unclear etiology or when conservative treatments fail, surgical options such as pars plana vitrectomy or surgical evacuation of subretinal hemorrhage may be considered.

#### Precautions: ####

Careful monitoring is essential to assess for spontaneous resolution or progression of the aneurysm.

The decision to intervene with laser treatment should be carefully weighed against the potential risks, such as inducing retinal artery occlusion or macular damage.

Management of underlying systemic conditions, particularly hypertension, is crucial in preventing further vascular complications.

**#### Disease Name: ####**

**Retinal Artery Occlusion, RAO**

#### Disease Description: ####

Retinal artery occlusion (RAO) includes central retinal artery occlusion (CRAO) and branch retinal artery occlusion (BRAO). RAO is an ophthalmic emergency primarily caused by embolism or thrombosis leading to retinal artery blockage. Embolism often occurs at the narrowest point where the central retinal artery passes through the optic nerve sheath, while CRAO may result from thrombus formation behind the lamina cribrosa. The prognosis for CRAO is generally poor. Reports indicate that in a series of CRAO patients, only 20% achieved a final visual acuity of 20/60 or better. BRAO has a more favorable natural course; even without treatment, 80% of patients achieve some visual improvement if their vision was worse than 20/40.

#### Patient Information: ####

##### Patient Age: #####

67 years old

##### Medical History: #####

The patient had a history of retinal artery occlusion (RAO), specifically either central retinal artery occlusion (CRAO) or branch retinal artery occlusion (BRAO).

##### Current Medications: #####

Patients with RAO are on medications for intraocular pressure reduction.

#### Condition of the Disease: ####

##### Lesion Location and Size: #####

The embolus occurred at the narrowest part of the central retinal artery where it pierces the optic nerve's dural sheath.

The size of the lesion is related to the area affected by the occlusion and the resulting ischemia.

#### Indications: ####

Patients with Central Retinal Artery Occlusion (CRAO), especially those with severe vision loss.

Patients with Branch Retinal Artery Occlusion (BRAO), often presenting with visual field defects; early detection of symptoms is particularly suitable for this treatment.

#### Treatment: ####

##### Method 1: Transluminal Nd:YAG Laser Embolectomy/Embolysis (TYE) #####

This technique involves using an Nd:YAG laser to dissolve or remove the embolus causing the occlusion, aiming to restore blood flow to the retina. The treatment begins with low energy (0.5-1 mJ) and gradually increases until embolus rupture, movement, or the appearance of small bubbles is observed. Energy increments are typically 0.2-0.8 mJ per step.

Embolus Management: The laser is focused on the embolus, aiming to fragment or displace it into the vitreous with controlled energy increases. Mild vitreous hemorrhage may occur, managed by applying pressure with a contact lens for a few minutes.

Energy Management: Higher pulse energy (≥ 2.4 mJ) does not significantly improve vision but is associated with an increased need for vitrectomy due to potential vitreous hemorrhage obstructing further laser treatment.

Procedure: The laser is focused on the embolus, gradually increasing energy to achieve embolus dissolution or removal. Direct laser application may be used to dislodge the embolus.

#### Precautions: ####

Risk of Hemorrhage: The procedure is associated with risks, particularly vitreous or subretinal hemorrhage, which typically resolves within 1-4 weeks.

Immediate Management: Hemorrhage should be addressed immediately, with vitrectomy performed if necessary.

High-Energy Pulses: Higher energy pulses should be reserved for patients with severe vision loss due to the increased risk of complications.

**#### Disease Name: ####**

**Glaucoma**

#### Disease Description: ####

Glaucoma is an ophthalmic condition that leads to optic nerve damage, often associated with elevated intraocular pressure. The damage to the optic nerve results in gradual vision loss and can ultimately lead to blindness. There are various types of glaucoma, including open-angle glaucoma, angle-closure glaucoma, and normal-tension glaucoma. Acute angle-closure glaucoma is an emergency, typically causing sudden severe eye pain, headache, nausea, blurred vision, and vision loss, requiring immediate treatment to prevent permanent vision loss.

#### Patient Information: ####

##### Patient Age: #####

60 years old

##### Medical History: #####

The patient had a history of elevated intraocular pressure (IOP).

Family history of glaucoma

Diabetes

Hypertension

Migraines

##### Current Medications: #####

Prostaglandin analogs

Beta-blockers

Alpha-adrenergic agonists

Carbonic anhydrase inhibitors

Rho kinase inhibitors

Miotic or cholinergic agents

#### Condition of the Disease: ####

##### Lesion Location and Size: #####

Unknown

#### Indications: ####

Early stages may show no obvious symptoms. As the condition progresses, peripheral visual field defects appear as patchy blind spots. In advanced stages, central vision is affected, making it difficult to see objects in the center.

Severe headache and eye pain.

Nausea or vomiting.

Blurred vision with halos or colored rings around lights.

Redness of the eye.

Early stages may show no obvious symptoms. Gradual blurring of vision occurs as the disease progresses, leading to loss of peripheral vision.

Infant shows signs of light sensitivity or cloudy corneas. Frequent blinking or tearing when not crying (infant characteristics). Blurred vision, worsening myopia, and headaches.

Halos around lights. Blurred vision during physical activities. Gradual loss of side vision.

#### Treatment: ####

##### Method 1: Medications #####

Prostaglandins: Promote the outflow of aqueous humor, lowering intraocular pressure. Medications include latanoprost (Xalatan), travoprost (Travatan Z), tafluprost (Zioptan), bimatoprost (Lumigan), and latanoprostene bunod (Vyzulta). Side effects may include mild redness and stinging, darkening of the iris, darkening of the eyelid skin, and blurred vision. These medications are typically used once daily.

Beta Blockers: Reduce the production of aqueous humor, helping to lower intraocular pressure, e.g., timolol (Betimol, Istalol, Timoptic) and betaxolol (Betoptic S). Side effects may include breathing difficulties, slowed heart rate, low blood pressure, impotence, and fatigue. Depending on the condition, these medications are used once or twice daily.

Alpha-Adrenergic Agonists: Reduce the production of aqueous humor and promote its outflow. Examples include apraclonidine (Iopidine) and brimonidine (Alphagan P, Qoliana). Side effects may include irregular heartbeats, high blood pressure, fatigue, red, itchy, or swollen eyes, and dry mouth. These medications are usually used twice daily, but sometimes three times a day.

Carbonic Anhydrase Inhibitors: Reduce the production of aqueous humor, e.g., dorzolamide and brinzolamide (Azopt). Side effects may include a metallic taste, frequent urination, tingling in the fingers and toes, and others. These medications are usually used twice daily, but sometimes three times daily.

Rho Kinase Inhibitors: Lower intraocular pressure by inhibiting rho kinase. These medications include netarsudil (Rhopressa), used once daily. Side effects may include eye redness and discomfort.

Miotic or Cholinergic Agents: Increase the outflow of aqueous humor, e.g., pilocarpine (Isopto Carpine). Side effects may include headaches, eye pain, small pupils, blurred vision, and nearsightedness. These medications are typically used up to four times a day. Due to potential side effects and the need for frequent use, these medications are not commonly used.

##### Method 2: Surgical and Other Treatments #####

Laser Therapy: If eye drops are not tolerated, laser trabeculoplasty may be considered. It can also be performed if medication fails to slow disease progression. The procedure is typically performed in the ophthalmologist's office using a small laser to help drain fluid from the angle between the iris and cornea. Full effects may take several weeks to appear.

Filtering Surgery: This surgery, known as trabeculectomy, involves making an opening in the sclera (the white part of the eye) to allow fluid to drain out of the eye.

Drainage Tubes: In this procedure, the ophthalmologist inserts a small drainage tube into the eye to help drain excess fluid, lowering intraocular pressure.

Minimally Invasive Glaucoma Surgery (MIGS): The ophthalmologist may recommend MIGS to lower intraocular pressure. These procedures generally require less postoperative care and have a lower risk compared to trabeculectomy or drainage devices. MIGS is often performed in conjunction with cataract surgery. Various MIGS techniques are available, and the ophthalmologist will discuss which procedure is suitable for you.

Acute Angle-Closure Glaucoma Treatment: Acute angle-closure glaucoma is an emergency. If diagnosed with this condition, immediate treatment is required to relieve eye pressure. This typically involves medication and laser or other surgical procedures. You may undergo a laser peripheral iridotomy, where a small hole is made in your iris using a laser to allow fluid to flow through, widening the drainage angle and relieving eye pressure.

#### Precautions: ####

Medication Use: Some medications may cause systemic side effects, such as heart problems or high blood pressure, so care should be taken to avoid the medication entering the systemic bloodstream when using eye drops.

Postoperative Management: Patients who undergo surgery need regular follow-up visits to monitor intraocular pressure and other potential changes within the eye. If eye pressure rises again, further surgery may be necessary.

**#### Disease Name: ####**

**Branch Retinal Artery Occlusion, BRAO**

#### Disease Description: ####

Branch Retinal Artery Occlusion (BRAO) is an obstruction of a retinal artery branch due to thrombosis or embolism. This leads to localized retinal ischemia, which affects vision, particularly if the macular region is involved. Patients typically present with sudden, painless vision loss. The condition is commonly caused by atherosclerosis, but may also result from cardiac emboli, blood disorders, or other systemic diseases.

#### Patient Information: ####

##### Patient Age: #####

49 years old

##### Medical History: #####

The patient had a history of branch retinal artery occlusion (BRAO).

##### Current Medications: #####

Unknown

#### Condition of the Disease: ####

##### Lesion Location and Size: #####

The embolus was located at the proximal inferior arterial arcade.

#### Indications: ####

Patients with Branch Retinal Artery Occlusion, particularly those presenting with sudden unilateral vision loss.

Patients with severe cases, especially those involving the macular region, as these cases pose a greater visual threat and require prompt intervention.

#### Treatment: ####

##### Method 1: Retinal Embolectomy #####

This procedure is typically performed in conjunction with a standard three-port pars plana vitrectomy (PPV). The use of a contact lens with high magnification and stereoscopic enhancement aids in the procedure. A complete posterior vitreous detachment is induced using diluted 50% intravitreal triamcinolone acetonide, and any uncolored vitreous covering the embolus is removed.

Vessel Sheath Incision: A 25-gauge microvitreoretinal (MVR) blade is used to carefully incise the vessel sheath along the blood flow direction until the small arterial wall is completely incised. To prevent intraoperative bleeding, intraocular pressure is temporarily raised to 50-60 mmHg during the incision.

Embolus Removal: The embolus is grasped and removed from the arterial lumen using intravitreal forceps. The intraocular pressure is then gradually reduced, and fluid-air exchange is performed to stabilize the retina.

Intraoperative Caution: Careful handling is crucial to avoid vitreous hemorrhage caused by vessel rupture, which could severely affect postoperative visual recovery.

#### Precautions: ####

Preoperative Preparation: A detailed preoperative assessment is necessary, including medical history, physical examination, and imaging studies, to determine the suitability of the patient for surgery.

Intraoperative Care: Maintaining stable intraocular pressure during the procedure is essential to avoid retinal hemorrhage, which could compromise surgical outcomes.

Postoperative Care: Regular follow-up is required post-surgery to monitor visual recovery and provide necessary supportive treatment to enhance vision improvement.

**#### Disease Name: ####**

**Diabetic Macular Edema, DME**

#### Disease Description: ####

Diabetic Macular Edema is a common retinal complication in patients with diabetes, typically caused by capillary leakage leading to swelling in the macular region. It results in blurred vision and, if untreated, can lead to permanent vision loss. DME management often requires a combination of laser therapy and anti-VEGF (vascular endothelial growth factor) injections.

#### Patient Information: ####

##### Patient Age: #####

50 years old

##### Medical History: #####

The patient was a diabetic.

Examination revealed circinates and hemorrhages temporal to the fovea, along with significant cystoid macular edema.

##### Current Medications: #####

The patient was on general diabetic management medications, including insulin or oral hypoglycemic agents.

#### Condition of the Disease: ####

##### Lesion Location and Size: #####

The lesion was located in the macular area with cystoid macular edema and hemorrhages, particularly temporal to the fovea. The edema was significant.

#### Indications: ####

A 50-year-old male diabetic patient with vision reduced to 20/80, accompanied by cystoid macular edema and hemorrhage.

#### Treatment: ####

##### Method 1: Combination Anti-VEGF Injection and Grid Laser Therapy #####

Use of 100 μm spot size, titrated power of 200 mW, with a 40% setting.

A semicircular grid pattern was applied, followed by the injection of ranibizumab (Lucentis, Genentech).

#### Precautions: ####

Laser Treatment Precision: Precise control of laser treatment is critical to avoid excessive retinal damage.

Combination Therapy: Combining anti-VEGF treatment reduces the frequency of treatments and provides better efficacy.

**#### Disease Name: ####**

**Diabetic Macular Edema, DME**

#### Disease Description: ####

Diabetic Macular Edema is a common retinal complication in patients with diabetes, typically caused by capillary leakage leading to swelling in the macular region. It results in blurred vision and, if untreated, can lead to permanent vision loss. DME management often requires a combination of laser therapy and anti-VEGF (vascular endothelial growth factor) injections.

#### Patient Information: ####

##### Patient Age: #####

52 years old

##### Medical History: #####

The patient was a diabetic.

Fundus evaluation revealed diffuse macular edema and hemorrhages around the macular area.

##### Current Medications: #####

The patient was on general diabetic management medications, including insulin or oral hypoglycemic agents.

#### Condition of the Disease: ####

##### Lesion Location and Size: #####

The lesion involved diffuse macular edema with hemorrhages around the macular area.

#### Indications: ####

A 52-year-old female diabetic patient with vision reduced to 20/200, accompanied by diffuse macular edema and hemorrhage.

#### Treatment: ####

##### Method 1: Grid Laser Therapy with Anti-VEGF Injection #####

Use of 100 μm spot size, with a 30% setting and 200 mW power.

Ranibizumab injection administered post-laser treatment.

#### Precautions: ####

Diffuse Macular Edema: For patients with diffuse macular edema, combined laser and anti-VEGF injection therapy provides optimal results.

**#### Disease Name: ####**

**Diabetic Macular Edema, DME**

#### Disease Description: ####

Diabetic Macular Edema is a common retinal complication in patients with diabetes, typically caused by capillary leakage leading to swelling in the macular region. It results in blurred vision and, if untreated, can lead to permanent vision loss. DME management often requires a combination of laser therapy and anti-VEGF (vascular endothelial growth factor) injections.

#### Patient Information: ####

##### Patient Age: #####

60 years old

##### Medical History: #####

The patient was a diabetic.

Examination revealed circinates and hemorrhages temporal to the fovea, but OCT showed a normal foveal contour with no cystoid edema.

##### Current Medications: #####

The patient was on general diabetic management medications, including insulin or oral hypoglycemic agents.

#### Condition of the Disease: ####

##### Lesion Location and Size: #####

The lesion was located temporal to the fovea, characterized by circinates and hemorrhages.

#### Indications: ####

1. A 60-year-old male diabetic patient with vision reduced to 20/80, accompanied by hemorrhage in the macular region, but without cystoid edema.

#### Treatment: ####

##### Method 1: Grid Laser Therapy with Anti-VEGF Injection #####

Use of 150 mW, 20 ms duration, and 30%-40% coverage grid laser therapy.

No anti-VEGF injection; laser therapy only.

#### Precautions: ####

Laser Monotherapy: Laser treatment alone can effectively control localized macular edema without the need for anti-VEGF injections.

**#### Disease Name: ####**

**Acute Angle Closure Glaucoma**

#### Disease Description: ####

Acute Angle Closure Glaucoma is an ophthalmic emergency caused by the sudden blockage of the anterior chamber angle, leading to a rapid increase in intraocular pressure (IOP). This condition is often due to the forward displacement of the peripheral iris, which blocks the trabecular meshwork, thereby preventing the outflow of aqueous humor. A common mechanism includes pupillary block, where the lens presses against the iris, causing aqueous humor to accumulate in the posterior chamber and pushing the iris forward, further obstructing the angle. Normal IOP ranges from 8 to 21 mmHg, but during an acute angle closure glaucoma attack, IOP can rise significantly, resulting in severe eye pain, blurred vision, halos around lights, nausea, and vomiting.

#### Patient Information: ####

##### Patient Age: #####

51 years old

##### Medical History: #####

Degenerative disc disease in the lower back

Started wearing reading glasses at age 42, currently uses progressive bifocals with hyperopic correction for distance

No prior eye surgeries, trauma, amblyopia, or strabismus

##### Current Medications: #####

Multivitamins

Vicodin prn (used for back pain a few days per month)

#### Condition of the Disease: ####

##### Lesion Location and Size: #####

Shallow anterior chamber angle 360 degrees OD, no view of the angle structures, hazy cornea OD, mid-dilated iris OD

Shallow angle with view of trabecular meshwork OS

Elevated intraocular pressure (OD: 62 mmHg, OS: 11 mmHg)

CDR (cup-to-disc ratio) of 0.5 OD, 0.4 OS, with sharp optic disc margins

#### Indications: ####

Acute eye pain and vision loss: The patient presents with severe right eye pain and blurred vision, accompanied by rainbow-colored halos and nausea.

Significantly elevated intraocular pressure: Examination reveals that the patient's right eye has an IOP of 62 mmHg, far exceeding the normal range of 8-21 mmHg.

Narrowed anterior chamber angle and shallow anterior chamber: Gonioscopy shows that the right eye has a narrowed and unclear angle structure, while the left eye, although having a narrow angle, shows visible trabecular meshwork.

Moderately dilated and sluggishly reactive pupil: The right pupil is moderately dilated and has a sluggish response to light, indicating impaired aqueous humor outflow.

#### Treatment: ####

##### Method 1: Peripheral Iridectomy #####

The treatment for acute angle closure glaucoma can be managed through laser or surgical peripheral iridectomy. This procedure creates an additional opening in the peripheral iris to restore the flow of aqueous humor from the posterior chamber to the anterior chamber, thereby reducing the pathological pressure gradient.

The goal of laser peripheral iridectomy is to retract the iris away from the trabecular meshwork and restore normal aqueous drainage. This procedure is generally curative for the affected eye. Prophylactic peripheral iridectomy may be performed on the unaffected eye to prevent future acute attacks.

#### Precautions: ####

High Risk for Contralateral Eye: Patients who experience one episode of acute angle closure glaucoma have a high risk of developing the condition in the opposite eye within the next 5-10 years. Even after IOP reduction, follow-up is essential to ensure that the angle remains open.

Pressure Drop Considerations: IOP may decrease shortly after an attack due to ciliary body ischemia and reduced aqueous production rather than angle reopening.

**#### Disease Name: ####**

**Acute Conjunctivitis of Both Eyes**

#### Disease Description: ####

Acute conjunctivitis is an inflammatory response of the conjunctival tissue, usually caused by viral infections, occurring around the eyeball and eyelid conjunctiva. The most common cause is adenovirus, while other potential pathogens include coxsackie virus, enterovirus, molluscum contagiosum virus, and systemic viral syndromes such as measles, mumps, influenza, and rhinovirus. Acute conjunctivitis is often associated with upper respiratory infection symptoms or a history of exposure to infected individuals, typically starting in one eye and spreading to the other within a few days.

#### Patient Information: ####

##### Patient Age: #####

11 years old

##### Medical History: #####

Born at term without complications

No history of eye trauma, surgery, amblyopia, strabismus, or contact lens use

##### Current Medications: #####

None

#### Condition of the Disease: ####

##### Lesion Location and Size: #####

Mild conjunctival injection OU with no chemosis

Crusted dry flaky material on eyelashes OU, clear corneas, and deep, quiet anterior chambers OU

Visual acuity OD: 20/20, OS: 20/25

CDR 0.2 in both eyes, with sharp optic disc margins, flat macula, normal vessels and peripheral retina OU

#### Indications: ####

Ocular irritation and itching: The patient reports four days of ocular irritation and itching, starting with the left eye and spreading to the right eye one day later.

Mild yellow discharge: Both eyes exhibit mild yellow discharge, leading to difficulty opening the eyelids in the morning.

History of exposure: The patient was exposed to a common cold virus (contact with a neighbor with a cold).

Mild visual impairment: Slightly reduced visual acuity in the left eye (20/25), while the right eye has normal visual acuity (20/20).

Mild conjunctival hyperemia: Slit lamp examination shows mild conjunctival hyperemia in both eyes.

Ocular findings include conjunctival hyperemia, chemosis, hemorrhage, follicular conjunctival reaction, tearing, preauricular lymphadenopathy, subepithelial corneal infiltrates, eyelid edema, conjunctival membranes or pseudomembranes, and/or corneal epithelial defects.

#### Treatment: ####

##### Method 1: Fluorescein Staining #####

Fluorescein can help detect corneal epithelial defects. If severe purulent discharge, chronic signs and symptoms, or significant corneal findings are present, cultures should be performed to detect bacterial conjunctivitis.

##### Method 2: Treatment of Viral Conjunctivitis #####

Primarily involves artificial tears and cold compresses.

#### Precautions: ####

Antibiotic Use: Topical antibiotics are not necessary unless bacterial etiology is suspected.

Steroid Drops: Generally avoided but may be helpful in the most severe cases (evidence of membranes/pseudomembranes) during recovery.

Avoid Topical Anesthetics: These can hinder healing and should not be used.

Contact Lens Wearers: Should avoid wearing lenses until symptoms and signs resolve.

Prognosis: Viral conjunctivitis typically resolves on its own within two weeks.

Complications: Membranes/pseudomembranes may cause permanent conjunctival scarring and chronic epithelial subepithelial corneal infiltrates, leading to visual impairment. Reassessment by an ophthalmologist is crucial in such cases.

Hygiene: Handwashing and other disinfection techniques (changing pillowcases and towels) are important to prevent the spread of the infection.

**#### Disease Name: ####**

**Traumatic Corneal Abrasion**

#### Disease Description: ####

Corneal abrasion refers to the disruption of the corneal epithelium, typically caused by trauma, contact lens use, or foreign objects. This patient's corneal abrasion was caused by trauma (kicked in the eye by a grandchild), resulting in severe eye pain, tearing, photophobia, and blurred vision. Corneal abrasions cause significant discomfort due to the rich nerve supply in the cornea. Typical symptoms include intense foreign body sensation, eye pain, excessive tearing, and an inability to open the eye due to pain and light sensitivity.

#### Patient Information: ####

##### Patient Age: #####

70 years old

##### Medical History: #####

Hypertension (treated with hydrochlorothiazide)

Cataract surgery OU 3 years ago

No history of contact lens use, amblyopia, or other eye diseases

##### Current Medications: #####

Hydrochlorothiazide

#### Condition of the Disease: ####

##### Lesion Location and Size: #####

Left eye (OS): 2 mm by 2 mm corneal epithelial defect with positive fluorescein staining, mild lower lid swelling, 1+ conjunctival injection, no corneal stromal infiltrate or thinning

Visual acuity: OD 20/20, OS 20/50 PH NI

CDR 0.2 in both eyes, with sharp optic disc margins, flat macula, normal vessels and peripheral retina OU

#### Indications: ####

Corneal epithelial disruption due to trauma or foreign objects, manifesting as eye pain, foreign body sensation, tearing, and photophobia.

Decreased vision, especially if the corneal abrasion is located on the visual axis.

No history of contact lens use or other eye diseases but with a clear history of ocular trauma.

#### Treatment: ####

##### Method 1: Topical Anesthesia and Fluorescein Staining #####

Use fluorescein-stained eye drops containing an anesthetic or fluorescein strips for corneal abrasion examination. During the examination, apply topical anesthetic eye drops for patient comfort but do not allow the patient to take the anesthetic drops home to prevent ongoing corneal epithelial defects and corneal melting, leading to permanent vision loss.

##### Method 2: Antibiotic Treatment and Lubrication #####

For simple corneal abrasions, use lubricants and antibiotics to prevent infection. Antibiotic eye drops or ointment formulations can be used in conjunction with artificial tears or ointments (e.g., erythromycin ointment, four times daily for five days or until the abrasion heals). Symptoms typically begin to improve within 24-48 hours. The patient should avoid wearing contact lenses until the cornea has completely healed.

#### Precautions: ####

Do Not Provide Anesthetic Eye Drops for Home Use: To prevent ongoing corneal damage and potential permanent vision loss.

Avoid Contact Lenses: Ensure the patient does not use contact lenses until the cornea has fully healed to prevent worsening of the condition or infection.

Close Monitoring: Severe corneal abrasions may lead to corneal scarring, irregular epithelium, or recurrent erosion syndrome, requiring close observation.

Follow-Up Examination: The patient should have a follow-up examination with an ophthalmologist within one to two days to ensure the abrasion is healing and no complications arise.

**#### Disease Name: ####**

**Postoperative Endophthalmitis**

#### Disease Description: ####

Endophthalmitis is an inflammation of the internal structures of the eye, usually in response to an infection. Postoperative endophthalmitis can be categorized as acute (occurring within 6 weeks post-surgery) or delayed (occurring more than 6 weeks post-surgery). Common pathogens in acute postoperative endophthalmitis include Staphylococcus aureus, Staphylococcus epidermidis, and Streptococcus species. Delayed postoperative endophthalmitis is more likely caused by organisms such as Staphylococcus epidermidis, Corynebacterium, Candida, or Propionibacterium acnes. This patient exhibited typical symptoms of postoperative endophthalmitis, including severe eye pain and decreased vision, two days after cataract surgery in the left eye.

#### Patient Information: ####

##### Patient Age: #####

69 years old

##### Medical History: #####

Type 2 diabetes mellitus (controlled with metformin, last HgA1c of 7, no neuropathy or nephropathy)

Hypertension (mild, controlled with lisinopril)

History of bilateral cataracts, post uncomplicated cataract surgeries (R eye 2 months ago, L eye 2 days ago)

##### Current Medications: #####

Prednisolone acetate 1% 4x/day L eye

Polytrim 4x/day L eye

Metformin

Lisinopril

#### Condition of the Disease: ####

##### Lesion Location and Size: #####

Left eye (OS): Microcystic edema throughout cornea, small keratic precipitates noted in inferior cornea, fibrin strands to the incision temporally, 1 mm hypopyon in the anterior chamber, 3+ cells and flare, very hazy view of the anterior vitreous, poor view of macula

Visual acuity: OD 20/30, OS 20/80 (post-op day #1 OS: 20/25)

CDR 0.4 in both eyes with sharp optic disc margins, retina attached 360 degrees OS

#### Indications: ####

Patients with a recent history of ocular surgery, particularly those experiencing severe eye pain, vision loss, photophobia, and floaters within six weeks postoperatively.

Patients with a history of ocular trauma, which may lead to infectious endophthalmitis.

Patients with diabetes or other immune system disorders are more susceptible to endogenous endophthalmitis.

#### Treatment: ####

##### Method 1: Workup and Treatment #####

The diagnosis and treatment of endophthalmitis include obtaining vitreous and/or aqueous humor cultures and treating with broad-spectrum antibiotics. Ultrasound examination may be performed if the retinal view is poor. If the visual acuity is better than light perception, a "tap and inject" procedure is typically performed—obtaining vitreous and/or aqueous humor samples for culture, followed by the injection of broad-spectrum antibiotics. If the visual acuity is light perception or worse, immediate vitrectomy is usually performed to obtain a vitreous sample for culture and inject antibiotics. Further treatment is based on the response, culture results, and antibiotic sensitivity studies.

##### Method 2: Emergency Management #####

Endophthalmitis is an ophthalmic emergency that can cause devastating damage to the eye, and treatment must begin as soon as possible. Any delay may result in worse visual outcomes, making time a critical factor in treatment.

##### Method 3: Antibiotic Therapy #####

Based on culture results and antibiotic sensitivity, the antibiotic treatment regimen should be adjusted to target the specific pathogen causing the infection. Broad-spectrum antibiotics, such as cephalosporins or aminoglycosides, are generally used to cover both Gram-positive and Gram-negative bacteria.

#### Precautions: ####

Prompt Diagnosis and Treatment: Immediate vitreous tap and injection should be performed once endophthalmitis is suspected to reduce the risk of infection spread and improve treatment outcomes.

Follow-Up: Patients should be closely monitored after initial treatment to assess the reduction of ocular inflammatory response and adjust the treatment plan as needed.

Preventing Complications: Complications of endophthalmitis may include severe visual impairment, corneal edema, and retinal detachment. Early intervention and appropriate treatment are crucial.

**#### Disease Name: ####**

**Globe Rupture**

#### Disease Description: ####

Globe rupture refers to a full-thickness laceration of the ocular wall (cornea or sclera), resulting in the exposure of the eye's interior to the external environment. Common causes include blunt trauma that ruptures a previous surgical wound and penetrating trauma with both entry and exit wounds in the globe. This patient exhibited typical symptoms of globe rupture, including eye pain, redness, tearing, and vision loss, after striking her right eye on a glass headboard during a fall from bed.

#### Patient Information: ####

##### Patient Age: #####

2 years old

##### Medical History: #####

Healthy child, born at term

No history of ocular surgery, trauma, amblyopia, or strabismus

##### Current Medications: #####

None

#### Condition of the Disease: ####

##### Lesion Location and Size: #####

Right eye (OD): 3 mm corneal laceration at 8 o'clock in horizontal configuration, extending from the limbus towards the visual axis

Flat anterior chamber OD, peaked iris to the wound

Trace diffuse hyperemia OD

No good view of the fundus in OD due to cooperation

Clear cornea OS, deep and quiet anterior chamber OS, CDR 0.1 with sharp optic disc margins, flat macula OS

#### Indications: ####

Patients with a clear history of ocular trauma, particularly those presenting with severe eye pain, sudden vision loss, ocular redness, and tearing.

Physical examination revealing full-thickness corneal or scleral lacerations, a shallow anterior chamber, irregular pupil shape (e.g., peaked pupil), intraocular foreign bodies, vitreous hemorrhage, or retinal detachment.

#### Treatment: ####

##### Method 1: Emergency Surgical Repair #####

Immediate surgical repair of the laceration is necessary to restore the integrity of the globe. Preoperatively, the patient should receive analgesics, antibiotics, and antiemetics, and the injured eye should be covered to prevent further damage. Tetanus prophylaxis should be administered while waiting for surgery.

##### Method 2: Postoperative Management #####

The outcome of globe rupture repair is unpredictable, with visual prognosis primarily depending on the initial visual acuity at the time of injury. Lifelong monitoring of the injured eye is required to prevent complications such as sympathetic ophthalmia (an inflammatory response in the uninjured eye). For eyes with no potential for vision recovery, enucleation (removal of the eye) may be performed to reduce the risk of immune activation and address severe eye pain.

#### Precautions: ####

Minimize Manipulation of the Injured Eye: Avoid performing intraocular pressure measurements to prevent further damage. During the examination, minimize manipulation of the injured eye and ensure a thorough examination of the uninjured eye, with dilation if necessary.

Long-Term Monocular Protection: Patients are advised to wear protective eyewear postoperatively and for life to protect the uninjured eye from further injury.

Prevent Sympathetic Ophthalmia: For eyes with no potential for visual recovery, consider enucleation to prevent the occurrence of sympathetic ophthalmia and alleviate severe eye pain.

In most cases, orbital CT scans are needed to identify any foreign bodies, check for scleral shape abnormalities (e.g., posterior ruptures may be missed on clinical examination), and examine orbital bone fractures. For cases where the posterior pole cannot be examined, a B-scan ultrasound may indicate intraocular foreign bodies, retinal detachment, or vitreous hemorrhage, but care should be taken not to apply unnecessary pressure to the globe.

**#### Disease Name: ####**

**Traumatic Hyphema**

#### Disease Description: ####

Hyphema refers to the presence of blood within the anterior chamber of the eye. When a sufficient amount of blood accumulates, it may deposit in the angle of the anterior chamber, covering the anterior segment structures. This patient presented with redness and pain in the left eye, along with blurred vision, following a blow to the eye with an elbow during a law enforcement action, consistent with the clinical presentation of traumatic hyphema. Hyphema is graded as follows: Grade I hyphema - blood covers less than one-third of the lower anterior chamber; Grade II - blood covers one-third to one-half; Grade III - blood covers one-half to nearly the entire anterior chamber; Grade IV - the anterior chamber is completely filled with blood (if black, referred to as "eight-ball hyphema"). The source of bleeding is typically ruptured vessels in the ciliary body.

#### Patient Information: ####

##### Patient Age: #####

42 years old

##### Medical History: #####

Sickle cell trait, type 2 diabetes mellitus, hypertension

No history of ocular surgery or trauma

##### Current Medications: #####

Metformin

Glipizide

Aspirin 81mg

Hydrochlorothiazide

#### Condition of the Disease: ####

##### Lesion Location and Size: #####

Left eye (OS): Hyphema obscuring 1/3 of the anterior chamber, mild conjunctival hyperemia 360 degrees, moderate upper lid edema, slightly hazy view of fundus

Visual acuity: OD 20/20, OS 20/40

CDR 0.3 in both eyes, no evidence of retinal tear or detachment

#### Indications: ####

Patients presenting with significant eye pain, vision loss, ocular redness, and hyphema following blunt ocular trauma.

Patients with a history of sickle cell disease or family history, especially in African American or Mediterranean patients, should be screened for the risk of sickle cell disease.

#### Treatment: ####

##### Method 1: Comprehensive Management #####

Treatment Focus: When treating hyphema, it is important to emphasize complete bed rest. Additionally, the patient's head should be elevated to allow the blood to settle in the lower anterior chamber, preventing obstruction of the visual axis. The injured eye should be shielded to prevent further trauma. Non-NSAID analgesics should be used for pain relief. Cycloplegic agents (such as atropine or scopolamine) and topical steroids are recommended to treat associated iritis and prevent posterior synechiae. If intraocular pressure is elevated, topical β-blockers are the first-line therapy, but additional medications may be needed to maintain normal IOP. Surgical indications include corneal blood staining, non-resolving total hyphema, and elevated intraocular pressure. The surgical removal threshold is lower in patients with sickle cell disease due to their higher risk of permanent vision loss.

#### Precautions: ####

Screening for Sickle Cell Disease: Special attention should be given to African American and Mediterranean patients due to their risk of sickle cell disease. Careful monitoring and control of intraocular pressure are essential to prevent vision loss from trabecular meshwork obstruction.

Avoid Rebleeding: Strict adherence to bed rest and eye protection is necessary to prevent rebleeding and worsening of the condition. Avoid strenuous activities and increased ocular pressure.

Postoperative Management: Patients should be followed frequently within the first two weeks to monitor intraocular pressure and manage any other complications. The risk of rebleeding is highest between 3 to 7 days post-injury and is associated with a worse prognosis. Complications of hyphema include posterior synechiae, peripheral anterior synechiae, corneal blood staining, glaucoma, and optic nerve atrophy due to elevated IOP. Prognosis ranges from poor to excellent, depending on the severity of the initial injury. Patients should have annual follow-ups to monitor for glaucoma and other complications.

**#### Disease Name: ####**

**Rhegmatogenous Retinal Detachment (RRD)**

#### Disease Description: ####

Rhegmatogenous retinal detachment (RRD) refers to the condition where liquefied vitreous passes through a retinal break into the space between the retinal pigment epithelium and the neurosensory retina, leading to retinal detachment. Patients typically report a sudden decrease in vision, accompanied by a "curtain-like" shadow and early symptoms such as flashes of light and floaters. In this case, the patient developed retinal detachment three weeks after cataract surgery in the left eye, likely due to retinal tears caused by vitreous traction. Post-cataract surgery, the increased vitreous cavity space and vitreous displacement may lead to retinal traction and tear formation.

#### Patient Information: ####

##### Patient Age: #####

61 years old

##### Medical History: #####

Hypertension (well controlled with medication)

Osteoarthritis

History of cataract surgery in the left eye 3 weeks ago

##### Current Medications: #####

Hydrochlorothiazide

Lisinopril

#### Condition of the Disease: ####

##### Lesion Location and Size: #####

Left eye (OS): Large area of subretinal fluid extending from 10 o'clock to 4 o'clock with fluid under the macula, indicating mac off retinal detachment. Horseshoe tear identified at 2 o'clock. Small amount of vitreous hemorrhage noted at the site of the tear.

Visual acuity: OD 20/25, OS counting fingers at 3 feet

CDR 0.3 in both eyes with sharp disc margins

#### Indications: ####

Patients with a recent history of cataract surgery, presenting with flashes of light, increased floaters, and sudden vision loss, particularly with shadowing in the central or peripheral vision.

Ophthalmic examination revealing pigment cells in the vitreous cavity (Shafer's sign) or peripheral retinal tears.

#### Treatment: ####

##### Method 1: Surgical Treatment #####

Rhegmatogenous retinal detachment typically requires surgical intervention. Common surgical methods include scleral buckle surgery, pneumatic retinopexy, and vitrectomy. The goal of the surgery is to relieve vitreous traction and reattach the retina to the underlying choroid. Current surgical techniques have a high success rate, with anatomical reattachment achieved in 80-90% of cases.

#### Precautions: ####

Comprehensive Ophthalmic Examination: Any patient reporting flashes or increased floaters should undergo a thorough ophthalmic examination. Besides measuring vision and intraocular pressure, a dilated examination of both eyes is crucial. The presence of pigment cells in the vitreous or other signs indicating retinal tears, especially in the peripheral retina, should be checked.

Follow-Up and Monitoring: Postoperative follow-up is necessary to monitor retinal reattachment and vision recovery. Patients with retinal tears or detachment should be educated to recognize any sudden changes in vision and seek immediate medical attention.

Prognosis Management: The visual prognosis after surgery depends on the severity of the detachment and whether the macula is involved. If the macula is not involved, 87% of patients may recover vision to 20/50 or better. When the macula is involved, only one-third to half of the patients may recover vision to 20/50 or better. If macular detachment is repaired within one week, 75% of patients may recover vision to 20/70 or better; if repaired within 1 to 8 weeks, this proportion drops to 50%.

**#### Disease Name: ####**

**Infantile Esotropia and Amblyopia of the Left Eye**

#### Disease Description: ####

Esotropia refers to the misalignment of the eyes, which can manifest in various forms. The most common forms are esotropia (inward deviation of the eyes) and exotropia (outward deviation of the eyes). Infantile esotropia is defined as a nasal misalignment of the eyes that occurs within the first 6 months of life, with no other ocular abnormalities. Although the exact cause is unclear, it is related to abnormal development of stereopsis, motor processing, and ocular motor function. While vision in both eyes may be normal, up to 40% of infants with esotropia may develop amblyopia. Amblyopia is characterized by poor vision in one eye despite the absence of detectable abnormalities during clinical examination, usually due to disrupted visual input to the brain during infancy and early childhood, leading to underdevelopment of the visual system. In this case, the patient developed amblyopia in the left eye due to eye misalignment.

#### Patient Information: ####

##### Patient Age: #####

6 months old

##### Medical History: #####

Born at term without complications

No history of ocular surgery, trauma, amblyopia, or strabismus

##### Current Medications: #####

None

#### Condition of the Disease: ####

##### Lesion Location and Size: #####

Left eye (OS): Esotropic deviation, as noted by corneal reflection test and cover-uncover test; 25 prism diopters base out on alternate-cover test. Mild hyperopia (+1.00) OU, no astigmatism.

Visual acuity: Fixes and follows OU, responds to light in all quadrants.

No optic nerve hypoplasia, normal macula and retinal vessels OU.

#### Indications: ####

Manifest esotropia within the first 6 months of life with no other ocular abnormalities.

Infants with a family history of refractive errors or amblyopia should be closely monitored for early signs of infantile esotropia.

Signs of amblyopia in the left eye associated with infantile esotropia, such as abnormal gaze reflex or eye deviation.

#### Treatment: ####

##### Method 1: Early Intervention #####

If detected early and treated aggressively, amblyopia can be partially reversed. Treatment is most effective during infancy but may still be beneficial up to 9 or 10 years of age while the visual system is still developing. Treatments include occlusion therapy or atropine penalization, which restricts the non-amblyopic eye, forcing the amblyopic eye to accept visual stimuli and promote vision recovery.

##### Method 2: Surgical Treatment #####

If amblyopia is caused by esotropia, surgical correction of eye misalignment is often necessary. The surgery aims to restore proper eye alignment, thereby improving the development of binocular vision and coordination.

#### Precautions: ####

Comprehensive Ophthalmic Examination: The potential causes of amblyopia include anisometropia (unequal refractive error between the eyes), strabismus (misalignment of the eyes), visual deprivation (secondary to cataract, ptosis, etc.), and organic diseases (such as optic nerve hypoplasia, retinoblastoma). Therefore, the diagnosis of amblyopia should include refraction, cover/uncover testing, and dilated fundus examination.

Early Detection and Treatment: Early detection and treatment of infantile esotropia are crucial. Parents should monitor any abnormal eye deviation in their infant and seek evaluation by an ophthalmologist promptly.

**#### Disease Name: ####**

**Leukocoria - Suspected Congenital Cataracts**

#### Disease Description: ####

Leukocoria refers to an abnormal white reflection from the pupil, typically caused by intraocular pathology. Congenital cataracts are a common cause of leukocoria and may be associated with intrauterine infections, metabolic disorders, malignancies, or genetic defects. In this case, a newborn was found to have absent red reflexes in both eyes within 2 hours of birth, replaced by a grayish-white reflection. The mother reported flu-like symptoms early in pregnancy, which may be related to an intrauterine infection causing the congenital cataracts.

#### Patient Information: ####

##### Patient Age: #####

2 hours old

##### Medical History: #####

Birth history: 38-week gestation, normal spontaneous vaginal delivery without complications

Mother recalls flu-like symptoms in early pregnancy

##### Current Medications: #####

None

#### Condition of the Disease: ####

##### Lesion Location and Size: #####

Both eyes (OU): Dense central opacities in the lens, with no view of the anterior vitreous or fundus

No other abnormalities in the cornea, conjunctiva, anterior chamber, or iris

Visual acuity: Reacts to light in both eyes (OD and OS)

Pupils: Equal, round, and reactive to light

#### Indications: ####

Newborns or infants presenting with leukocoria during an eye examination, especially if a normal red reflex cannot be obtained using a direct ophthalmoscope.

Infants with a history of intrauterine infection or whose mothers had flu-like symptoms during pregnancy.

Children with a family history of congenital cataracts or other congenital eye diseases.

#### Treatment: ####

##### Method 1: Surgical Treatment #####

The optimal time for surgical treatment of congenital cataracts is within 4 to 8 weeks of birth to minimize the risk of amblyopia and sensory nystagmus. Surgery typically includes cataract removal, primary posterior capsulotomy, and anterior vitrectomy due to the high rate of capsular opacification. Most patients remain aphakic after surgery and are fitted with contact lenses shortly afterward. Secondary intraocular lens implantation may be performed later as the eye matures.

#### Precautions: ####

Comprehensive History Taking: Obtaining a thorough maternal pregnancy history, medication history, and family history of eye diseases is crucial. The differential diagnosis of leukocoria includes retinoblastoma (the most common intraocular malignancy in children), congenital cataracts, retinopathy of prematurity, persistent hyperplastic primary vitreous, Coats disease, familial exudative vitreoretinopathy, retinal detachment, and corneal opacity.

Visual Function Assessment: Early assessment of visual acuity in each eye individually is necessary to determine the impact of the cataracts on vision. Additionally, B-scan ultrasonography can help assess the posterior segment of the eye to rule out posterior abnormalities.

Postoperative Management and Follow-Up: Postoperative evaluation and treatment of amblyopia are essential, and patients require lifelong follow-up to maximize their visual potential.

**#### Disease Name: ####**

**Wet Age-Related Macular Degeneration (Wet AMD)**

#### Disease Description: ####

Age-related macular degeneration (AMD) is a degenerative retinal disease that primarily affects central vision. Wet AMD is caused by the development of pathological choroidal neovascular membranes (CNV) beneath the retina or retinal pigment epithelium, which disrupts the retinal structure, leading to fibrovascular scarring and vision loss. In this case, the patient presented with soft drusen in both eyes and a subretinal hemorrhage in the macular area of the left eye, near the fovea, consistent with the features of wet AMD. The patient reported decreased central vision and the presence of a dark spot in the visual field, suggesting possible CNV formation.

#### Patient Information: ####

##### Patient Age: #####

72 years old

##### Medical History: #####

Hypertension, hypercholesterolemia, coronary artery disease (s/p myocardial infarction and stenting 5 years ago)

No history of eye surgeries, trauma, amblyopia, or strabismus

##### Current Medications: #####

Aspirin, Plavix, Lipitor, Lisinopril, Metoprolol

#### Condition of the Disease: ####

##### Lesion Location and Size: #####

Left eye (OS): Subretinal hemorrhage near the fovea, 1 disc area in size, associated with scattered soft drusen within the arcades. CDR 0.6. Blurry spot near the center of vision with wavy lines on Amsler grid.

Right eye (OD): Scattered large soft drusen within the arcades, CDR 0.5, no signs of hemorrhage

Visual acuity: OD 20/30, OS 20/100

#### Indications: ####

Fundus examination revealing soft drusen or subretinal hemorrhage, particularly near the macula.

Reports of decreased central vision or detection of wavy lines or central scotomas on an Amsler grid test.

Elderly patients with a family history of AMD or other risk factors (e.g., smoking).

#### Treatment: ####

##### Method 1: Anti-VEGF Therapy #####

This is currently the most effective treatment for wet AMD. Anti-VEGF drugs inhibit the growth of new blood vessels, slowing or halting the progression of CNV. Timely treatment can stabilize or improve vision in 2/3 of patients.

##### Method 2: AREDS Vitamin Formula #####

Patients with advanced dry AMD or early wet AMD are recommended to use this vitamin formula, which includes specific combinations of vitamins A, C, E, zinc, and copper to reduce the risk of disease progression.

##### Method 3: Other Treatment Options #####

Photodynamic therapy and laser photocoagulation are alternatives, though less effective than anti-VEGF therapy, and are typically used when anti-VEGF therapy is ineffective or unsuitable.

#### Precautions: ####

Continuous Monitoring: Patients are advised to regularly monitor their vision using an Amsler grid. Any new onset of scotomas or metamorphopsia should prompt immediate medical evaluation to detect and treat CNV at the earliest stage.

Timely Treatment: Wet AMD can lead to severe central vision loss, and early detection and treatment are key to preventing irreversible vision loss.

Regular Follow-Up: Patients receiving anti-VEGF therapy should have regular follow-ups to monitor the treatment response and adjust the treatment plan as needed.

Lifestyle Changes: Smoking cessation and blood pressure control are recommended to reduce the risk of wet AMD and slow its progression.

**#### Disease Name: ####**

**Chemical Burn**

#### Disease Description: ####

A chemical burn is an ocular injury caused by contact with acidic, alkaline, or neutral substances. Alkaline substances are typically more dangerous than acids as they penetrate ocular tissues more easily, causing deeper injuries. This 31-year-old male patient sustained an injury to his left eye and surrounding skin from an automobile battery explosion, resulting in immediate severe burning pain and vision loss. The patient was exposed to sulfuric acid, a common acidic substance found in car batteries, leading to significant corneal edema and epithelial defects, requiring emergency treatment to prevent further damage and irreversible vision loss.

#### Patient Information: ####

##### Patient Age: #####

31 years old

##### Medical History: #####

No significant medical history

No history of ocular surgery, trauma, amblyopia, or strabismus

##### Current Medications: #####

None

#### Condition of the Disease: ####

##### Lesion Location and Size: #####

Left eye (OS): Diffuse corneal edema with central epithelial defect covering 75% of the corneal area, 3+ conjunctival injection with 1+ chemosis, area of epithelial loss on fluorescein exam, erythematous upper and lower lids, hazy anterior chamber view.

Visual acuity: OD 20/20, OS 20/200

pH of tears: 6 upon arrival, neutralized to 7 after 3L saline irrigation

#### Indications: ####

Ocular burns following exposure to acidic or alkaline substances, presenting with severe pain, blurred vision, and photophobia.

Longer exposure time or exposure to high-concentration chemicals may result in more severe injuries, requiring urgent care.

Abnormal tear film pH (lower or higher than the normal range) indicates the need for further irrigation and treatment.

#### Treatment: ####

##### Method 1: Irrigation and Initial Management #####

Immediate ocular irrigation should begin as soon as a chemical burn is suspected, with isotonic saline as the preferred solution. If saline is unavailable, clean water can be used. Irrigation should continue for at least 10-15 minutes or until the tear film pH returns to approximately 7. During irrigation, local anesthetics may be used to enhance patient tolerance. A sterile cotton swab may be used to sweep the fornices to remove residual chemical particles.

##### Method 2: Medication Therapy #####

After irrigation, treatment should be tailored based on the severity of the burn and may include artificial tears, topical antibiotics, topical steroids, mydriatic agents, and analgesics. For more severe injuries, glaucoma medications may be necessary to maintain normal intraocular pressure, or surgical intervention may be required.

#### Precautions: ####

Immediate Treatment: Chemical burns are an ocular emergency, and immediate irrigation is critical to reducing the severity of the injury. The pH of the tear film should be reassessed after each examination to ensure it remains within the normal range.

Preventing Complications: Even after thorough irrigation, permanent damage may occur. Timely further treatment and follow-up are necessary based on the severity of the injury.

Special Considerations: Alkaline burns require particular attention as these substances penetrate ocular tissues more rapidly, causing more severe injuries.

Severe chemical burns may result in corneal scarring, severe dry eye, cataracts, and elevated intraocular pressure (glaucoma). These patients may require lifelong ophthalmic care, and in cases of significant corneal and conjunctival scarring, corneal transplantation may not be feasible, leading to long-term vision impairment.

**#### Disease Name: ####**

**Optic Nerve Compression**

#### Disease Description: ####

Optic nerve compression refers to the condition where the optic nerve is compressed by adjacent structures, leading to impaired optic nerve function, typically manifesting as decreased vision, visual field defects, and optic disc swelling. The patient in this case is a 31-year-old female social worker who experienced fluctuating vision and gradually worsening blurred vision in both eyes, particularly in the right eye. The symptoms worsened when lying down. She also reported persistent headaches and tinnitus. Further imaging revealed a large suprasellar mass compressing the right optic nerve and displacing the left optic nerve.

#### Patient Information: ####

##### Patient Age: #####

31 years old

##### Medical History: #####

Obesity

No history of eye surgeries, trauma, amblyopia, or strabismus

##### Current Medications: #####

Multivitamin

#### Condition of the Disease: ####

##### Lesion Location and Size: #####

Right eye (OD): CDR 0.1 with blurry margins, obscuration of small vessels, visual field defects superionasally and superiotemporally.

Left eye (OS): CDR 0 with significant elevation of the optic disc, obscuration of small and large vessels, disc hemorrhages, and visual field defects in all quadrants.

Visual acuity: OD 20/40, OS 20/200

Positive APD in the left eye, bilateral optic nerve head swelling

MRI: Large suprasellar mass abutting the right optic nerve and displacing the left optic nerve.

#### Indications: ####

Gradually worsening vision loss and visual field defects, particularly when accompanied by headaches, blurred vision, and tinnitus.

Imaging studies showing optic nerve compression by adjacent structures, such as tumors or aneurysms.

Clinical examination revealing optic disc swelling and visual field defects.

#### Treatment: ####

##### Method 1: Surgical Treatment #####

For optic nerve compression caused by a tumor or other space-occupying lesion, surgical resection or decompression is typically required to alleviate the pressure on the optic nerve and prevent further vision loss. The choice of surgery depends on the size and location of the tumor and its effect on surrounding structures.

##### Method 2: Radiation Therapy #####

If the tumor is malignant or cannot be completely resected, radiation therapy may be used as an adjunct to control tumor growth and reduce pressure on the optic nerve.

##### Method 3: Medication Therapy #####

In certain cases, particularly tumor-related lesions, corticosteroids and other medications may be used to reduce inflammation and edema. Steroids may help temporarily alleviate tumor pressure on the optic nerve and improve symptoms.

#### Precautions: ####

Timely Diagnosis and Intervention: Optic nerve compression can lead to permanent vision loss, so early diagnosis and treatment are essential to maximize visual function preservation.

Postoperative Monitoring: Postoperative patients need regular ophthalmic and neurological follow-ups to closely monitor optic nerve function recovery.

Comprehensive Treatment Plan: Depending on the nature of the tumor and the specific condition of the patient, a combination of surgery, radiation therapy, and medication may be required to achieve the best treatment outcomes.

Follow-Up and Monitoring: Regular follow-up is necessary post-treatment to monitor vision and visual field recovery and to detect any signs of complications or tumor recurrence.

**#### Disease Name: ####**

**Central Retinal Vein Occlusion (CRVO)**

#### Disease Description: ####

Central Retinal Vein Occlusion (CRVO) occurs due to the blockage of the central retinal vein at the level of the lamina cribrosa. This condition typically presents with diffuse retinal hemorrhages, retinal edema, and dilated, tortuous retinal veins. Other characteristic retinal signs include cotton wool spots, optic disc edema, and hemorrhages, along with potential development of neovascularization in the iris, optic nerve, or retina. CRVO can be classified into two types: perfused (most common) and ischemic/non-perfused. Perfused CRVO is usually milder, with better vision and a more favorable prognosis, and has a lower risk of neovascularization in the iris or retina. However, about one-third of perfused CRVO cases may progress to ischemic CRVO. Vision loss in CRVO may be caused by macular edema, macular ischemia, or complications from neovascularization (e.g., neovascular glaucoma or vitreous hemorrhage).

#### Patient Information: ####

##### Patient Age: #####

58 years old

##### Medical History: #####

Hypertension, history of pulmonary embolism following left foot surgery, menopause at 51

No history of ocular surgeries, trauma, amblyopia, or strabismus

##### Current Medications: #####

Calcium supplement, Multivitamin, Hydrochlorothiazide, Lisinopril

#### Condition of the Disease: ####

##### Lesion Location and Size: #####

Left eye (OS): Edematous optic nerve with blurred margins, scattered intraretinal hemorrhages in all four quadrants, few cotton wool spots, macular thickening with loss of foveal light reflex, tortuous vessels.

OCT findings: Intra and subretinal fluid in the retina, distortion of normal foveal architecture.

Visual acuity: OD 20/25, OS 20/200

Trace APD in the left eye, no neovascularization observed.

#### Indications: ####

Patients with sudden, painless vision loss, especially those with a "blood and thunder" appearance of the retina.

Patients with hypertension, diabetes, vascular disease, or primary open-angle glaucoma, typically aged 50 years or older.

Exclusion of other retinal conditions such as diabetic retinopathy, central retinal artery occlusion, anemia or leukemia-related retinopathy, and traumatic retinal diseases.

#### Treatment: ####

##### Method 1: Laser Photocoagulation #####

For patients with neovascularization, panretinal photocoagulation (PRP) should be performed to prevent further complications. Macular edema associated with CRVO usually responds poorly to focal laser therapy, with visual prognosis similar to untreated patients.

##### Method 2: Anti-VEGF Therapy #####

In recent years, intravitreal injections of anti-VEGF agents (such as Avastin) have shown promising results in improving macular edema related to CRVO.

##### Method 3: Steroid Treatment #####

In certain cases, intravitreal steroid injections have been used to treat macular edema.

#### Precautions: ####

Regular follow-up: Monthly vision, pupillary response, and anterior segment examinations are required during the first 3-6 months after diagnosis to monitor for ischemic CRVO or neovascularization and to provide timely intervention.

Prognosis: The prognosis of CRVO depends on whether it is ischemic or non-ischemic. Approximately 50% of non-ischemic CRVO patients retain vision of 20/200 or better, while only 10% of ischemic CRVO patients achieve this level of vision. About 60% of non-perfused CRVO patients develop neovascularization, posing a higher risk of complications.

**#### Disease Name: ####**

**Proliferative Diabetic Retinopathy (PDR)**

#### Disease Description: ####

Diabetic retinopathy is a retinal vascular complication caused by diabetes and is mainly classified into non-proliferative diabetic retinopathy (NPDR) and proliferative diabetic retinopathy (PDR). NPDR typically presents with microaneurysms, retinal hemorrhages, and hard exudates. As the disease progresses, increasing retinal ischemia may lead to the overexpression of vascular endothelial growth factor (VEGF), resulting in the formation of neovascularization, known as PDR. These abnormal and fragile neovascular vessels can easily lead to vitreous hemorrhage and tractional retinal detachment, ultimately causing severe vision loss and even blindness.

#### Patient Information: ####

##### Patient Age: #####

35 years old

##### Medical History: #####

Type 2 Diabetes Mellitus (5 years), Hypercholesterolemia, Obesity, Hypertension

No history of eye surgeries, trauma, amblyopia, or strabismus

##### Current Medications: #####

Lisinopril

Hydrochlorothiazide

Metformin

Simvastatin

#### Condition of the Disease: ####

##### Lesion Location and Size: #####

Right eye (OD): Neovascularization of the disc involving ~50% of the disc, flat macula with multiple microaneurysms and hard exudates > 500 microns away from the fovea, no clinically significant macular edema, multiple dot-blot hemorrhages in the periphery in all four quadrants.

Left eye (OS): CDR 0.40 with sharp disc margins, flat macula with multiple microaneurysms and hard exudates > 500 microns away from the fovea, no clinically significant macular edema, multiple dot-blot hemorrhages in all four quadrants.

Fluorescein angiography shows abnormal vasculature and capillary dropout, with hyperfluorescence at the neovascularization of the disc.

Visual acuity: OD 20/40, OS 20/40

#### Indications: ####

Diabetic patients, especially those with long-standing diabetes and poor glycemic control, should be regularly screened for diabetic retinopathy.

Patients presenting with symptoms such as blurred vision, floaters, and photophobia, particularly those with a history of diabetes.

Clinical examination revealing retinal neovascularization or vitreous hemorrhage should prompt consideration of PDR.

#### Treatment: ####

##### Method 1: Panretinal Photocoagulation (PRP) #####

According to the Diabetic Retinopathy Study, panretinal laser photocoagulation is effective in reducing vision loss in PDR patients with "high-risk characteristics." These characteristics include neovascularization on more than one-third of the optic disc, neovascularization on the optic disc accompanied by preretinal or vitreous hemorrhage, and extramacular neovascularization with preretinal or vitreous hemorrhage. The laser reduces the metabolic oxygen demand of the retina, helping neovascularization to regress.

##### Method 2: Vitrectomy #####

For patients with non-clearing vitreous hemorrhage or tractional retinal detachment, laser treatment may no longer be effective, and vitrectomy may be required. This surgery aims to remove vitreous hemorrhage or fibrous tissue, restoring the normal structure of the retina and preventing further vision loss.

##### Method 3: Glycemic Control #####

Controlling blood glucose levels is the most important factor in preventing and slowing the progression of diabetic retinopathy. Diabetic patients are advised to maintain an HbA1c level below 7% to reduce the risk of developing retinopathy. Good communication between primary care physicians and ophthalmologists is essential for maintaining ocular health and preventing retinopathy.

#### Precautions: ####

Regular eye examinations: Diabetic patients should undergo a dilated eye exam at least once a year to monitor for signs of diabetic retinopathy.

Good glycemic control: Strict control of blood glucose levels is critical for preventing and slowing the progression of diabetic retinopathy. Patients should work closely with their doctors to ensure blood glucose levels remain within a reasonable range.

Early intervention: Once signs of retinopathy, such as neovascularization or vitreous hemorrhage, are detected, timely treatment, such as laser photocoagulation, should be administered to prevent further vision loss.

**#### Disease Name: ####**

**Grave’s Ophthalmopathy**

#### Disease Description: ####

Grave’s Ophthalmopathy, also known as thyroid-associated ophthalmopathy, is a condition characterized by changes in the orbital contents, primarily the extraocular muscles and orbital fat, due to an autoimmune process. This process is believed to be triggered by a reaction mediated by antibodies against the thyroid-stimulating hormone (TSH) receptor, although only 50% of patients test positive for specific immunoglobulins in blood tests. T lymphocytes also migrate to the orbit, triggering an immune response that leads to hypertrophy of the extraocular muscles (without affecting the tendons) and orbital congestion, which account for most of the clinical manifestations of Grave’s Ophthalmopathy. It is important to note that patients with thyroid eye disease may not necessarily have active thyroid disease.

#### Patient Information: ####

##### Patient Age: #####

43 years old

##### Medical History: #####

No significant medical history

No history of eye surgeries, trauma, amblyopia, or strabismus

##### Current Medications: #####

None

#### Condition of the Disease: ####

##### Lesion Location and Size: #####

Both eyes (OU): Mild lacrimal gland enlargement, mild chemosis and 1+ conjunctival injection, slight punctate epithelial erosions in the inferior cornea, mild upper eyelid fullness, and limited downward gaze with eyelid elevation.

Hertel exophthalmometry: 23mm OD, 22mm OS.

Visual acuity: OD 20/20, OS 20/20

#### Indications: ####

Patients presenting with symptoms such as eyelid retraction, dry eyes, diplopia, restricted extraocular muscle movement, excessive tearing, and eye irritation.

Physical examination findings may include proptosis (exophthalmos), incomplete eyelid closure (lagophthalmos), ocular misalignment (strabismus), eyelid swelling, corneal dryness, conjunctival edema, elevated intraocular pressure, and, in severe cases, optic nerve compression leading to decreased vision due to orbital swelling.

#### Treatment: ####

##### Method 1: Basic Treatment #####

Artificial tears can be used to relieve discomfort caused by corneal exposure. Eyelid surgery may be considered for severe eyelid retraction.

##### Method 2: Immunotherapy #####

For severe orbital congestion and optic neuropathy, high-dose corticosteroid therapy is the first choice. Immunomodulators such as cyclosporine or azathioprine can also be used as an alternative or adjunct to steroids.

##### Method 3: Surgical Treatment #####

In cases of severe proptosis and optic nerve compression, orbital decompression surgery may be required. If diplopia is caused by muscle hypertrophy and fibrosis, prism correction or strabismus surgery may be offered after the inflammatory response has been controlled. Eyelid surgery is recommended for patients with exposure keratopathy caused by levator muscle fibrosis.

#### Precautions: ####

Diagnosis and Imaging: Non-contrast orbital CT scans typically show bilateral extraocular muscle hypertrophy without tendon involvement. Other tests include TSH, free T3, and T4 levels to assess thyroid function.

Postoperative Risk Management: Patients undergoing surgery require close monitoring to assess the resolution of ocular inflammatory responses, especially after orbital decompression surgery, to ensure there is no further optic nerve compression or other complications.

Systemic Disease Management: The treatment of Grave’s Ophthalmopathy is independent of thyroid disease management, although controlling systemic thyroid function can have some impact on the overall prognosis of the disease.

**#### Disease Name: ####**

**Giant Cell Arteritis, GCA (or Temporal Arteritis)**

#### Disease Description: ####

Giant Cell Arteritis (GCA) is an inflammatory vasculitis primarily affecting medium to large arteries, particularly the vertebral artery, superficial temporal artery, posterior ciliary arteries, and ophthalmic artery. This condition is commonly seen in elderly individuals and can cause a range of systemic, neurological, and ophthalmic complications. Vision loss is one of the major morbidities associated with GCA, requiring early diagnosis and urgent treatment to prevent blindness. Patients typically present with sudden, painless vision loss, such as transient vision loss (amaurosis fugax) as seen in this case. Additionally, patients may exhibit prodromal or concurrent symptoms such as headache, jaw claudication, tenderness of the superficial temporal artery, proximal muscle and joint pain, anorexia, and weight loss. The diagnosis of GCA is confirmed by temporal artery biopsy, revealing giant cell infiltration in the arterial wall indicative of inflammation.

#### Patient Information: ####

##### Patient Age: #####

65 years old

##### Medical History: #####

Hypertension (well controlled), hyperlipidemia, smoking (1 pack/day for 40 years)

No history of eye surgeries, trauma, amblyopia, or strabismus

##### Current Medications: #####

Hydrochlorothiazide

Lisinopril

Simvastatin

#### Condition of the Disease: ####

##### Lesion Location and Size: #####

Right eye (OD): CDR 0.2 with one hemorrhage superiorly and cotton-wool spots around the optic nerve, mild to moderate leakage of the right optic nerve on fluorescein angiogram.

Left eye (OS): CDR 0.2 with sharp optic disc margins, no abnormalities.

Temporal artery biopsy: Presence of giant cells in the wall of the right temporal artery.

Visual acuity: OD 20/20, OS 20/25+1

Elevated ESR: 116

#### Indications: ####

Elderly patients aged 65 and above presenting with intermittent or sudden vision loss, headache, and other systemic symptoms such as weight loss and fatigue.

Patients with elevated ESR (erythrocyte sedimentation rate) and CRP (C-reactive protein) levels, indicative of an inflammatory state.

Patients with tenderness or pulselessness of the temporal artery should be highly suspected of GCA, and a temporal artery biopsy should be performed for confirmation.

#### Treatment: ####

##### Method 1: Systemic Corticosteroid Therapy #####

Once GCA is suspected, systemic corticosteroids should be administered immediately to reduce arterial wall inflammation and prevent irreversible blindness due to occlusion of the ophthalmic artery. Steroid treatment should begin before the temporal artery biopsy is conducted. If the patient presents with acute vision loss, intravenous corticosteroids may be administered. Even if vision has been lost for some time, steroid treatment should continue to minimize vision loss in the affected eye and prevent involvement of the other eye.

##### Method 2: Long-term Management #####

If the temporal artery biopsy is positive, or if there is a high clinical suspicion of GCA, the patient should continue oral corticosteroid therapy until symptoms resolve and ESR returns to normal. Treatment may last from 6 to 12 months. If significant side effects occur from high-dose oral corticosteroids, a steroid-sparing agent may be considered.

#### Precautions: ####

Urgent Treatment: GCA is an ophthalmic emergency, and any delay may result in worse visual outcomes. Corticosteroid therapy should be initiated as soon as possible to prevent further vision loss.

Follow-up Monitoring: Patients should be regularly monitored during treatment to assess the resolution of the inflammatory response and adjust the treatment plan as needed.

Prevention of Complications: GCA can lead to severe vision impairment, temporal artery occlusion, and worsening headaches, making early intervention and appropriate treatment crucial.

**#### Disease Name: ####**

**Primary Open-Angle Glaucoma, POAG**

#### Disease Description: ####

Primary Open-Angle Glaucoma (POAG) is an optic neuropathy associated with elevated intraocular pressure (IOP) and characterized by specific visual field defects. The main issue in POAG is a defect in the trabecular meshwork, leading to impaired aqueous humor outflow and subsequent elevation of IOP. Prolonged high IOP can cause thinning and damage to the optic nerve head, initially presenting as peripheral vision loss. Patients may not notice any vision loss for years, as their central vision remains intact. In POAG, elevated IOP, optic nerve head changes, and visual field defects occur without identifiable causes. Risk factors include central corneal thickness (the thinner the cornea, the greater the risk), race, age, and family history (siblings of POAG patients have a 10-fold increased risk).

#### Patient Information: ####

##### Patient Age: #####

61 years old

##### Medical History: #####

Hypertension

No history of eye surgeries, trauma, amblyopia, or strabismus

##### Current Medications: #####

Hydrochlorothiazide

#### Condition of the Disease: ####

##### Lesion Location and Size: #####

Right eye (OD): CDR 0.7 with sharp optic disc margins, no obvious rim thinning or disc hemorrhage. Visual acuity 20/20, IOP 21 mmHg.

Left eye (OS): CDR 0.8 with sharp optic disc margins, no obvious rim thinning or disc hemorrhage. Visual acuity 20/20, IOP 23 mmHg.

Automated visual field test shows superior arcuate defect in both eyes.

Gonioscopy: Open angles with minimal pigmentation in the trabecular meshwork.

Pachymetry: OD 560 µm, OS 551 µm (within normal range).

#### Indications: ####

Patients identified with elevated IOP (normal range is 8 to 21 mmHg) during routine eye exams, accompanied by optic nerve head abnormalities (such as increased cup-to-disc ratio) and visual field defects.

Patients with open-angle anterior chamber angles, optic nerve head changes, and visual field defects, without a history of secondary glaucoma.

Special attention should be given to patients with a family history of glaucoma or those with thin corneas.

#### Treatment: ####

##### Method 1: Medication Therapy #####

Topical eye drops are the first-line treatment for lowering IOP, which is the only modifiable risk factor. Classes of medications include prostaglandin analogs, adrenergic agonists, beta-blockers, and carbonic anhydrase inhibitors. The treatment goal is to reduce baseline IOP by 30% (target IOP below 21 mmHg).

##### Method 2: Surgical Treatment #####

Laser Trabeculoplasty: Recommended for patients who do not respond well to medication or need additional IOP reduction. The procedure increases aqueous humor outflow by stimulating the trabecular meshwork with laser.

Trabeculectomy: Indicated for patients who fail to respond to both medication and laser treatment. The surgery involves creating an opening in the sclera to allow fluid to drain and lower IOP.

Drainage Tube Surgery: A small drainage tube is implanted in the eye to help drain excess fluid, suitable for refractory glaucoma.

#### Precautions: ####

Early Diagnosis and Treatment: Regular eye exams are crucial for early detection and intervention since glaucoma is often asymptomatic in the early stages.

Long-Term Management: Glaucoma requires lifelong management. Patients should understand its chronic nature, adhere to their treatment plan, and attend regular follow-ups to prevent further vision deterioration.

Lifestyle Adjustments: Although no specific lifestyle changes have been proven to prevent glaucoma, patients should avoid eye trauma and manage other health conditions, such as hypertension, to reduce additional risks.

Follow-up and Monitoring: Patients need regular follow-up to monitor IOP, visual fields, and optic nerve status to ensure that glaucoma is not progressing or to adjust the treatment plan. Regular visual field tests and optic nerve fiber layer thickness measurements are crucial for monitoring disease progression.

**#### Disease Name: ####**

**X-linked Ocular Albinism**

#### Disease Description: ####

Ocular albinism (OA) is a condition caused by X-linked mutations in the GPR143 gene. Due to its X-linked inheritance pattern, OA is more common in males. OA results in abnormal melanogenesis and melanosome transport in the retina and choroid, leading to hypopigmented changes in these structures. However, patients typically have normal skin and hair color. The abnormal melanin biosynthesis is associated with incomplete macular development, potentially causing related conditions such as strabismus, amblyopia, nystagmus, low vision, and photophobia.

#### Patient Information: ####

##### Patient Age: #####

8 years old

##### Medical History: #####

No significant medical history

##### Current Medications: #####

Allegra

#### Condition of the Disease: ####

##### Lesion Location and Size: #####

Right eye (OD): Monocular elevation deficiency with a -3 limitation in supraduction, otherwise full extraocular movements. Normal C/D ratio of 0.2, normal macula, normal vessels, and normal periphery in both eyes.

Visual acuity: OD 20/50 +1, OS 20/50 +3

Stereo test: Animals 3/3, Circles 8/9

Abnormal head posture (AHP): 5-10 degree chin up.

No significant misalignment in primary gaze.

#### Indications: ####

Children, especially males, presenting with signs of decreased vision, nystagmus, photophobia, and macular underdevelopment.

Families with male members showing similar symptoms, while female members may have no symptoms but could exhibit retinal abnormalities (e.g., NIR or short-wavelength autofluorescence imaging revealing X-inactivation mosaicism).

Decreased macular reflex and blurred vision, especially when having difficulty seeing distant objects like a school whiteboard.

#### Treatment: ####

##### Method 1: Visual Correction #####

Although ocular albinism cannot be cured, vision can be improved with corrective lenses to address refractive errors. Magnifying glasses can also be used to assist with reading and other near tasks.

##### Method 2: Strabismus Surgery #####

If strabismus is present, surgical correction of the eye muscles can improve eye alignment.

##### Method 3: Genetic Counseling and Low Vision Resources #####

Patients should undergo regular ophthalmologic examinations to maximize vision protection. It is recommended that patients connect with genetic counselors and low vision clinics, or local organizations that provide tools such as magnifying glasses, computer-aided speech recognition programs, and large-print books. These organizations may also offer workshops and educational programs on living with low vision to maximize patients' quality of life.

#### Precautions: ####

Regular Follow-up: Since ocular albinism cannot be cured, regular ophthalmologic follow-up is essential to adjust vision correction methods and help patients manage vision loss.

Psychological Support: Because ocular albinism is a chronic condition, psychological support may be needed for patients and their families, especially for school-aged children facing learning and social challenges.

Avoiding Bright Light Exposure: Due to photophobia, patients should avoid bright light environments and wear appropriate sunglasses during outdoor activities to reduce light sensitivity.

**#### Disease Name: ####**

**Ocular Ischemic Syndrome (OIS) with secondary Neovascular Glaucoma**

#### Disease Description: ####

Ocular Ischemic Syndrome (OIS) is a condition characterized by ocular hypoxia resulting from occlusion or stenosis of the common or internal carotid arteries or their branches ipsilateral to the affected eye. The stenosis is typically severe, with approximately 90% or more occlusion. The reduced blood flow and oxygen delivery to the eye lead to the development of OIS. This condition most frequently occurs in elderly patients, with an average age of 65 years. The incidence is higher in males compared to females, possibly due to higher rates of cardiovascular disease in males. Risk factors include cardiovascular disease, hypertension, and diabetes mellitus. OIS is associated with a high mortality rate, with 40% of patients dying within five years of onset, primarily due to cardiovascular disease.

#### Patient Information: ####

##### Patient Age: #####

70 years old

##### Medical History: #####

Type II Diabetes Mellitus (complicated by peripheral neuropathy)

Ischemic heart failure

Atrial fibrillation, Recurrent iritis (OD)

Steroid-induced ocular hypertension (OD)

Keratoconjunctivitis sicca (OU)

Dry eye syndrome (OU)

Pseudophakia (OU)

##### Current Medications: #####

Apixaban

Brimonidine tartrate/timolol maleate ophthalmic solution (0.2%/0.5%, 1 drop OU twice daily)

Glimepiride

Hydrocodone/acetaminophen

Vitamin D3

#### Condition of the Disease: ####

##### Lesion Location and Size: #####

Right eye (OD): Visual acuity of 20/80 +2, corrected to 20/50 -2 with pinhole. Intraocular pressure: 20 mmHg. Superior peri-pupillary neovascularization of the iris. Trace pigmented anterior vitreous cell, vitreous syneresis. Dilated fundus exam shows temporally tilted disc with a CDR of 0.8, inferior and superior thinning, moderate epiretinal membrane temporally, scattered hard drusen, many microaneurysms, and numerous dot-blot hemorrhages in the mid-periphery (at least ¾ quadrants).

Left eye (OS): Visual acuity of 20/30 -1, corrected to 20/25 +2 with pinhole. Intraocular pressure: 10 mmHg. Dilated fundus exam shows temporally tilted disc with a CDR of 0.6, moderate epiretinal membrane temporally, scattered hard drusen, many microaneurysms, and dot-blot hemorrhages in 2/4 quadrants.

#### Indications: ####

Blurred vision, particularly in one eye, with a gradual onset over weeks to months.

Neovascularization of the iris (NVI) and iridocorneal angle.

Neovascular glaucoma with elevated intraocular pressure (IOP).

Mid-peripheral dot-blot hemorrhages in the retina.

Anterior segment findings such as flare, iris atrophy, corneal edema, and asymmetric cataracts.

History of cardiovascular conditions such as ischemic heart failure or atrial fibrillation.

#### Treatment: ####

##### Method 1: Primary Treatment of the Stenosed Vessel #####

Carotid Artery Imaging: Duplex carotid ultrasonography is crucial for diagnosing OIS, with the ability to non-invasively diagnose 75% of cases of carotid stenosis.

Carotid Endarterectomy (CEA): Used to treat carotid artery stenosis that is less than 100% occluded, with an efficacy of 70-90%. The procedure aims to restore blood flow and potentially reverse some effects of OIS.

Carotid Artery Stenting: Employed in cases where endarterectomy is not suitable to restore blood flow.

##### Method 2: Management of Ischemic Complications #####

Laser Panretinal Photocoagulation (PRP): Used to ablate non-functional peripheral retina tissue, reducing ischemic drive and neovascularization.

Intravitreal Anti-VEGF Injections: Anti-VEGF medications, such as Avastin (bevacizumab), decrease intraocular VEGF levels, reducing neovascularization.

Management of Neovascular Glaucoma: Surgical management may involve placing a tube shunt for IOP control, particularly for high IOP. Valved tube shunts may be necessary, and trabeculectomies are often less effective due to bleeding risks.

Medical management with IOP-lowering drops can serve as a temporary measure, but often is insufficient if the angle is closed.

#### Precautions: ####

Surgical Considerations: Avoidance of Arterial Bypass Surgery: Surgery is avoided when the artery is completely occluded, as collaterals may compensate, and ischemic damage may be irreversible.

Monitor for neovascularization and glaucoma development to prevent further visual loss and complications.

Regular follow-up with carotid artery imaging to assess stenosis progression.

Management of underlying cardiovascular conditions to reduce mortality risk.

**#### Disease Name: ####**

**Pigmentary Glaucoma**

#### Disease Description: ####

Pigmentary glaucoma (PG) is a form of glaucoma that occurs in the context of pigmentary dispersion syndrome (PDS). PDS is characterized by the dispersion of iris pigment into the anterior segment of the eye, leading to a constellation of findings including corneal endothelial pigment deposition (Krukenberg spindle), darkly pigmented trabecular meshwork, prominent mid-peripheral iris transillumination defects, iris backbowing, and the presence of a Scheie stripe (pigment deposition on the junction of the posterior lens capsule and vitreous face). The condition is often found in young, myopic individuals and can be exacerbated by physical activities that increase intraocular pressure (IOP), such as playing sports.

The dispersion of pigment occurs due to the rubbing of the iris pigment epithelium against the lens zonules, which is typically caused by a back-bowed iris. This pigment is then deposited in the posterior chamber or travels to the anterior chamber, where it can accumulate on the corneal endothelium and in the trabecular meshwork. Over time, this can lead to increased IOP and optic nerve damage, characteristic of glaucoma.

#### Patient Information: ####

##### Patient Age: #####

24 years old

##### Medical History: #####

No significant medical history

##### Current Medications: #####

latanoprost OU

#### Condition of the Disease: ####

##### Lesion Location and Size: #####

Both eyes (OU): Episodic haloes around lights, blurry vision, and transient pressure sensation in both eyes. Intraocular pressure: 32 mmHg OD, 25 mmHg OS. Notable findings include Krukenberg spindle on corneal endothelium, diffuse iris pigment specks, midperipheral radial iris transillumination defects, optic nerve cupping with a C/D ratio of 0.35 OD and 0.55 OS, heavily-pigmented trabecular meshwork, and iris backbowing on gonioscopy (E60c 4+ OU).

#### Indications: ####

Episodic blurry vision and rainbow-colored haloes around lights, especially after physical activities.

Elevated intraocular pressure (IOP) (32 mmHg OD, 25 mmHg OS in this case).

Presence of Krukenberg spindle on the corneal endothelium.

Diffuse iris pigment specks and mid-peripheral radial iris transillumination defects.

Optic nerve head cupping with asymmetric cup-to-disc ratio (0.35 OD, 0.55 OS).

Heavily pigmented trabecular meshwork and back-bowed iris observed during gonioscopy.

#### Treatment: ####

##### Method 1: Medical Management #####

Topical Anti-Glaucoma Medications: The patient was started on latanoprost OU to lower intraocular pressures. These medications are typically first-line treatment for managing elevated IOP in PG.

Pilocarpine: Pilocarpine may be used to prevent pigment liberation by causing the iris to move forward, reducing contact between the iris and zonules. Caution is required due to the risk of retinal detachment, particularly in young, myopic patients.

##### Method 2: Laser and Surgical Interventions #####

Laser Peripheral Iridotomy (LPI): The patient underwent laser peripheral iridotomy (LPI) OU to relieve reverse pupillary block caused by the backbowing of the iris. Post-procedure, the gonioscopic findings improved.

Laser Trabeculoplasty: Laser trabeculoplasty is effective in PG due to the heavily pigmented trabecular meshwork. Argon laser trabeculoplasty (ALT) is preferred by some clinicians over selective laser trabeculoplasty (SLT) due to the risk of IOP spikes following SLT.

Surgical Management: If medical and laser treatments fail to control IOP, surgical options such as trabeculectomy or seton placement may be considered. In cases of uncontrolled glaucoma, cyclodestruction of the ciliary body might be used as a last resort.

##### Method 3: Lifestyle Modifications #####

Avoidance of Jarring Exercise: The patient was advised to avoid activities that could exacerbate pigment dispersion, such as soccer or other sports involving sudden movements.

#### Precautions: ####

Regular monitoring of IOP and optic nerve status is essential to prevent progression of glaucoma.

Careful consideration of the risks and benefits of cholinergic agonists like pilocarpine, especially in young, myopic patients.

Post-laser and surgical follow-up to ensure stable IOP and to monitor for potential complications such as IOP spikes or retinal detachment.

**#### Disease Name: ####**

**Plateau Iris Syndrome**

#### Disease Description: ####

Plateau iris syndrome is a relatively uncommon form of primary angle closure glaucoma, which occurs more frequently in younger adults compared to pupillary block angle-closure glaucoma. The condition is caused by large or anteriorly positioned ciliary processes that push the peripheral iris forward, leading to a crowded or occluded iridocorneal angle and obstructed aqueous outflow. Despite a normal or shallow central anterior chamber depth, gonioscopy typically reveals a crowded angle with the characteristic "double-hump sign" due to the iris draping over the ciliary body.

#### Patient Information: ####

##### Patient Age: #####

47 years old

##### Medical History: #####

Iron deficiency anemia secondary to uterine fibroid

##### Current Medications: #####

None

#### Condition of the Disease: ####

##### Lesion Location and Size: #####

Both eyes (OU): Narrow angles by Van Herrick, gonioscopy findings of crowded anterior chamber angles with “double-hump” sign, indicating plateau iris configuration. Pre-laser peripheral iridotomy (LPI), anterior segment optical coherence tomography (OCT) showed narrow angles in both eyes.

#### Indications: ####

Narrow or occludable anterior chamber angle observed during gonioscopy.

Presence of the "double-hump sign" on indentation gonioscopy, indicating iris draping over the ciliary body.

Angle closure may occur spontaneously or after pupillary dilation.

Asymptomatic presentation with the diagnosis made during routine examination.

#### Treatment: ####

##### Method 1: Initial Treatment #####

Ancillary imaging modalities like ultrasound biomicroscopy and anterior segment optical coherence tomography (OCT) can aid in the diagnosis, although gonioscopy remains the primary tool for evaluating the iridocorneal angle and detecting the double-hump sign.

Laser Peripheral Iridotomy (LPI):

Peripheral laser iridotomy is typically the first-line intervention in patients suspected of having plateau iris syndrome. The procedure aims to relieve any element of pupillary block by creating a small hole in the iris to allow fluid to flow between the anterior and posterior chambers, thus reducing pressure in the eye.

Laser Settings Recommendations: For Laser Iridotomy (Argon and Nd): Argon Laser Peripheral Iridotomy: Spot size: 50 micrometers; Duration: 0.02-0.2 seconds; Power: 1 Watt; Lens: Abraham or Wise. For Nd Laser Peripheral Iridotomy, spot size and duration are fixed; Energy: 1-12 mJoules; Lens: Abraham or Wise.

In this case, LPI was performed in both eyes, improving the angle depth, but the angle in the left eye remained occludable.

##### Method 2: Advanced Treatment #####

Laser Iridoplasty:

For patients with persistent occludable angles or elevated intraocular pressure despite a patent iridotomy, laser iridoplasty may be necessary. This procedure involves placing laser burns at the peripheral iris, which causes the iris tissue to contract and pull away from the trabecular meshwork, thereby widening the angle.

Laser Settings Recommendations: Spot size: 200-500 micrometers; Duration: 0.2-0.5 seconds; Power: 150-300 mWatt; Lens: None or Goldmann 3 mirror.

In this case, the patient underwent successful laser iridoplasty in the left eye without complications, leading to improved angle depth as confirmed by repeat gonioscopy.

##### Method 3: Conventional Surgical Procedures #####

If laser treatments fail to control intraocular pressure, conventional surgical options such as trabeculectomy or tube-shunt surgery may be considered. These procedures aim to create a new pathway for fluid drainage from the eye, reducing intraocular pressure.

#### Precautions: ####

Regular monitoring of the angle status with repeat gonioscopy exams every 6 months.

Close observation for any signs of angle closure or elevated intraocular pressure, which may necessitate additional interventions.

Consideration of the risks and benefits of further surgical interventions if laser treatments are insufficient to maintain open angles and control intraocular pressure.

**#### Disease Name: ####**

**Primary Congenital Glaucoma (Infantile Glaucoma) with Buphthalmos, OS**

#### Disease Description: ####

Primary congenital glaucoma is a rare, developmental form of glaucoma that occurs within the first three years of life. It is characterized by an abnormality in the trabecular meshwork, which impairs aqueous outflow and leads to elevated intraocular pressure (IOP). The condition often presents with signs of ocular enlargement due to the increased pressure, which can cause buphthalmos ("ox eye"). Other characteristic features include corneal edema, corneal haze, and increased axial eye length. The disease typically manifests between 3-6 months of age, although cases presenting at birth tend to be more severe. While the precise cause of primary congenital glaucoma is not fully understood, it is believed that the trabecular meshwork remains immature and compressed due to abnormal migration of neural crest cells during development. Most cases are sporadic, but some cases have been associated with autosomal recessive inheritance, particularly involving mutations in the CYP1B1 gene.

#### Patient Information: ####

##### Patient Age: #####

3 years old

##### Medical History: #####

Unremarkable medical history, no previous ocular surgeries or trauma

##### Current Medications: #####

None

#### Condition of the Disease: ####

##### Lesion Location and Size: #####

Left eye (OS): Corneal stromal haze and edema, increased intraocular pressure (34 mmHg OS, 12 mmHg OD), increased horizontal corneal diameter (OS: 13 mm, OD: 11 mm), increased axial eye length (OS: 24.5 mm, OD: 20.7 mm), and increased cupping noted on fundus exam. Right eye is normal.

Visual acuity: Fix and follow for both eyes (OD and OS).

#### Indications: ####

Increased size of the left eye (OS) noted by parents at approximately 1 year of age.

Tearing (epiphora) and corneal haze in the affected eye.

Elevated intraocular pressure: 34 mmHg in the left eye (OS) compared to 12 mmHg in the right eye (OD).

Increased horizontal corneal diameter: 13 mm in the left eye (OS) compared to 11 mm in the right eye (OD).

Increased axial eye length: 24.5 mm in the left eye (OS) compared to 20.7 mm in the right eye (OD).

Gonioscopy findings: high iris insertion in the left eye (OS).

Dilated fundus examination (DFE): increased cupping in the left eye (OS).

#### Treatment: ####

##### Method 1: Diagnostic and Surgical Intervention #####

Examination Under Anesthesia (EUA): The patient was examined under anesthesia to obtain accurate IOP measurements, perform a detailed ocular exam, and confirm the diagnosis.

Surgical Treatment: The patient underwent a trabeculotomy in the left eye (OS) to surgically open the immature trabecular meshwork and restore normal aqueous outflow. The procedure was successful, and the IOP in the affected eye returned to normal (16 mmHg). No further surgery or pressure-lowering medications were required.

##### Method 2: Long-term Management #####

Potential Supplemental Therapy: If needed, supplemental medical therapy with topical medications may be indicated to maintain IOP control, particularly if surgical intervention does not fully normalize IOP.

#### Precautions: ####

Regular Monitoring: Postoperative follow-up to monitor IOP, corneal clarity, and axial eye growth is essential to ensure disease control and detect any signs of recurrence.

Early diagnosis and intervention are crucial to prevent permanent damage to the optic nerve and loss of vision.

**#### Disease Name: ####**

**Pseudoexfoliation Glaucoma**

#### Disease Description: ####

Pseudoexfoliation glaucoma is a form of open-angle glaucoma associated with the presence of pseudoexfoliation material, which is a fibrillar extracellular material that accumulates in the eye and other organs. This material is deposited on various ocular structures, including the lens capsule, iris, and trabecular meshwork, leading to increased intraocular pressure (IOP) and optic nerve damage. Pseudoexfoliation glaucoma can present unilaterally or bilaterally, often with asymmetry, and is more common in elderly individuals. It is the leading cause of unilateral glaucoma in the elderly.

In this case, the patient presented with unilateral advanced pseudoexfoliation glaucoma in the left eye (OS), characterized by a gradual, painless loss of vision and an IOP of 71 mmHg OS. The presence of white, fluffy material on the pupil margin, lens capsule, and a pigmented Sampaolesi's line on gonioscopy are indicative of pseudoexfoliation syndrome. The optic nerve in the affected eye showed almost complete cupping, while the visual field was severely compromised.

#### Patient Information: ####

##### Patient Age: #####

65 years old

##### Medical History: #####

No significant medical history, no medications, no family history of glaucoma

##### Current Medications: #####

None

#### Condition of the Disease: ####

##### Lesion Location and Size: #####

Left eye (OS): Best corrected visual acuity of barely hand motion vision, IOP of 71 mmHg, large optic nerve cup with almost complete cupping, Sampaolesi's line noted on gonioscopy, corneal edema, white fluffy material on the lens capsule, ground-glass appearance of anterior lens capsule, and bullseye lesion seen on retro-illumination.

Right eye (OD): Best corrected visual acuity of 20/20, IOP of 19 mmHg, normal optic nerve with 0.4 cup-to-disc ratio.

#### Indications: ####

Unilateral elevated intraocular pressure (71 mmHg OS).

Gradual, painless loss of vision in the affected eye.

Presence of pseudoexfoliation material on the pupil margin, lens capsule, and angle structures.

Optic nerve cupping with a cup-to-disc ratio of almost complete cup OS.

Severe visual field loss in the affected eye, with only temporal field remnants.

Gonioscopy revealing a pigmented Sampaolesi's line and moderately open angles.

#### Treatment: ####

##### Method 1: Medical Management #####

Topical Anti-Glaucoma Medications: The patient was treated with topical anti-glaucoma medications, which successfully reduced the IOP to the 30s OS. These medications are the first-line treatment to lower IOP and prevent further optic nerve damage.

##### Method 2: Laser and Surgical Interventions #####

Laser Trabeculoplasty: Laser trabeculoplasty is effective in pseudoexfoliation glaucoma due to the increased pigmentation of the trabecular meshwork, which enhances the laser’s effect. This procedure is often considered when topical medications are insufficient to control IOP.

#### Precautions: ####

Surgical Considerations: In advanced cases where visual potential is low, as in this patient, filtration surgery (trabeculectomy) may not be ideal due to the risk of complications such as sympathetic ophthalmia in the contralateral eye. Cyclodestruction of the ciliary body may be considered as a last resort for IOP control.

Considerations During Cataract Surgery: Although cataract surgery does not reduce the risk of glaucoma, it may be necessary in patients with significant lens opacity. Cataract surgery can be challenging in pseudoexfoliation glaucoma due to weakened lens zonules and a higher risk of lens subluxation into the posterior pole and vitreous loss during phacoemulsification.

Regular monitoring of IOP and optic nerve status is crucial to prevent further progression of glaucoma.

Consideration of the risks associated with surgical interventions, particularly in patients with low visual potential and advanced optic nerve damage.

Detailed preoperative planning for cataract surgery, with attention to the stability of the lens zonules and potential complications.

**#### Disease Name: ####**

**Acanthamoeba Keratitis**

#### Disease Description: ####

Acanthamoeba keratitis is a severe and vision-threatening infection of the cornea caused by Acanthamoeba, a free-living pathogenic protozoan. The organism exists either as active, motile trophozoites or dormant, double-walled cysts. Under duress, the organism converts to the cyst form, which is highly resistant to desiccation, freezing, chlorination, and other harsh environmental conditions. This infection was first recognized in contact lens wearers in the mid-1970s and has since been most commonly associated with contact lens use, accounting for approximately 80% of documented cases.

Acanthamoeba keratitis typically presents with severe eye pain, photophobia, decreased vision, redness, and a protracted clinical course. The pain experienced is often disproportionate to the clinical appearance. If untreated or misdiagnosed, the condition can lead to corneal perforation and may require penetrating keratoplasty (PKP).

#### Patient Information: ####

##### Patient Age: #####

39 years old

##### Medical History: #####

Mild hypertension and hypercholesterolemia

No history of ocular surgeries or trauma

##### Current Medications: #####

Valsartan

Atorvastatin

Topical Gatifloxacin and Tobramycin (every hour, OD)

Cyclopentolate BID (OD)

#### Condition of the Disease: ####

##### Lesion Location and Size: #####

Right eye (OD): 4x4 mm stromal ring infiltrate with surrounding WBC infiltration, keratic precipitates lining the endothelium, 0.5 mm hypopyon, small epithelial defects over the infiltrate, enlarged corneal nerves (radial perineuritis), 4+ conjunctival injection, 3+ anterior chamber cells, and 2+ flare reaction. Decreased corneal sensation. Confocal microscopy confirmed double-walled cysts of Acanthamoeba.

Left eye (OS): Normal

Visual acuity: OD – Count fingers at 6 inches, OS – 20/20

#### Indications: ####

Severe eye pain, photophobia, and redness in a contact lens wearer.

Visual acuity significantly reduced, as seen in the right eye with count fingers at 6 inches.

Presence of a stromal ring infiltrate with surrounding white blood cell infiltration.

Decreased corneal sensation in the affected eye.

Confocal microscopy showing inflamed corneal nerves, double-walled cyst structures, and Acanthamoeba trophozoites.

Histopathology confirming the presence of double-walled cysts in corneal scrapings.

#### Treatment: ####

##### Method 1: Initial and Acute Management #####

Epithelial Debridement: Early in the disease course, epithelial debridement is recommended to improve the penetration of anti-amoebic therapy, reduce the load of amoebic trophozoites, and obtain appropriate specimens for histopathology.

Topical Anti-Amoebic Therapy: The patient was started on chlorhexidine (CHX) 0.02% drops every hour while awake and oral itraconazole 200 mg BID. Topical polyhexamethylene biguanide (PHMB) 0.04% was added four times a day (QID), as it is effective against both trophozoite and cyst forms of Acanthamoeba.

Cycloplegics for Comfort: Cyclopentolate 1% BID was used to relieve pain by dilating the pupil and paralyzing the ciliary muscle.

Oral Antifungal Therapy: Oral azole antifungals such as itraconazole or ketoconazole were administered to help control the infection.

##### Method 2: Advanced Treatment #####

Intravenous Therapy: In cases where topical and oral treatments are insufficient, intravenous (IV) therapy with pentamidine may be employed. In this case, the patient received daily IV therapy with pentamidine due to persistent corneal thinning and worsening infection.

Topical Antibiotic Therapy: Tobramycin was added every 2 hours as secondary coverage when a secondary bacterial infection with Gram-negative rods was identified.

Steroid Therapy: Oral Prednisone 80 mg daily was started after some improvement was noted and was continued at a maintenance dose of 40 mg daily post-discharge.

##### Method 3: Surgical Intervention #####

Penetrating Keratoplasty (PKP): PKP was required due to persistent dense stromal infiltrates and corneal thinning. Post-surgery, the patient achieved significant visual recovery with best-corrected visual acuity improving to 20/25 three months after the procedure.

Post-Surgical Management: Post-PKP, the patient was maintained on CHX 0.04% and PHMB 0.04% 5x/day, Prednisolone 1% 4x/day, Atropine 1% twice daily, and continued oral antifungal therapy. Regular follow-up and selective suture removal were performed as necessary.

#### Precautions: ####

Acanthamoeba keratitis is challenging to treat due to the organism's resilience and ability to persist as dormant cysts. Therefore, long-term follow-up and careful monitoring for recurrence are essential.

The use of corticosteroids should be approached with caution and typically delayed until after the infection is under control to avoid exacerbating the condition.

The patient should be counseled on the importance of contact lens hygiene and the risks associated with exposure to contaminated water sources.

#### Disease Name: ####

Acute Corneal Hydrops

#### Disease Description: ####

Acute corneal hydrops is a condition characterized by the sudden onset of corneal edema, typically occurring in the setting of corneal ectatic disorders such as keratoconus. This condition arises due to the acute disruption of Descemet's membrane, leading to fluid accumulation in the corneal stroma. The focal corneal edema results from the compromised barrier function of Descemet's membrane, allowing fluid to enter and swell the corneal tissue. Hydrops can significantly impair vision and is often associated with symptoms such as pain, redness, and photophobia. Although most cases of acute corneal hydrops resolve spontaneously over several months, they may result in scarring and contour irregularity that could necessitate corneal transplantation.

#### Patient Information: ####

##### Patient Age: #####

51 years old

##### Medical History: #####

Keratoconus, rigid gas permeable (RGP) contact lens use in left eye, mild dry eye syndrome in both eyes

##### Current Medications: #####

Ciprofloxacin drops three times daily (OS)

Sodium chloride 5% (Muro 128) drops four times daily (OS)

Artificial tears as needed (OU)

#### Condition of the Disease: ####

##### Lesion Location and Size: #####

Left eye (OS): Inferior conical protrusion, severe inferior corneal edema with overlying microcystic edema and bullae, intact epithelium, deep anterior chamber, trace nuclear sclerosis in the lens. Pachymetry could not be measured due to corneal edema.

Right eye (OD): Normal cornea, pachymetry 541 µm

Visual acuity: OD 20/20 with soft contact lens, OS 20/100 without correction (pinhole to 20/60)

Intraocular pressure: OD 17 mmHg, OS 24 mmHg

#### Indications: ####

History of keratoconus with the use of rigid gas permeable (RGP) contact lenses.

Presentation with symptoms of left eye redness, pain, light sensitivity, and tearing for one week.

Visual acuity significantly reduced to 20/100 without correction, with pinhole improvement to 20/60 in the affected eye.

Presence of severe inferior corneal edema, microcystic edema, bullae, and conical corneal protrusion on slit-lamp examination.

Anterior segment optical coherence tomography (AS-OCT) showing massive inferior corneal edema, overlying epithelial bullae, and a break in Descemet's membrane.

#### Treatment: ####

##### Method 1: Conservative Management #####

Topical Medications: Prednisolone applied four times daily to reduce inflammation.

Topical Medications: Sodium Chloride 5% (Muro 128) Drops applied four times daily to reduce epithelial edema.

Topical Medications: Cyclopentolate applied twice daily for cycloplegia to reduce pain.

Bandage Contact Lens: A Kontur bandage contact lens was placed to provide comfort and protect the cornea.

Intraocular Pressure Management: Timolol administered once daily to manage ocular hypertension secondary to reactive inflammation.

##### Method 2: Surgical Consideration #####

Pneumatic Descemetopexy: Placement of an anterior chamber sulfur hexafluoride (SF6) gas bubble was offered to accelerate recovery by tamponading the Descemet's membrane break, but the patient declined due to upcoming air travel and work constraints.

Penetrating Keratoplasty (PK): PK was performed three weeks after the initial presentation due to persistent corneal scarring and contour irregularity, which impaired vision. PK is often the treatment of choice in cases where corneal scarring is significant and visual rehabilitation is needed.

#### Precautions: ####

Patients with acute corneal hydrops should be monitored closely for the resolution of edema and the potential development of visually significant scarring.

The choice of using a gas bubble for descemetopexy should consider patient factors such as the ability to position and travel restrictions.

In cases where conservative management is insufficient, surgical intervention with PK may be necessary, with close postoperative follow-up to monitor graft success and prevent complications.

**#### Disease Name: ####**

**Atopic Keratoconjunctivitis (AKC)**

#### Disease Description: ####

Atopic keratoconjunctivitis (AKC) is a chronic inflammatory disease of the eye that primarily affects individuals between their late teenage years and the fifth decade of life. It is characterized by chronic, bilateral conjunctivitis that often relapses and remits. The disease is closely associated with atopic dermatitis, present in 95% of cases, and asthma, present in 87% of cases. AKC patients commonly exhibit ocular itching, mucoid discharge, tearing, and periocular eczema. The condition can lead to severe complications, such as corneal neovascularization, ulcers, erosions, and posterior subcapsular cataracts. Without proper management, AKC can cause significant visual impairment due to corneal opacification.

#### Patient Information: ####

##### Patient Age: #####

52 years old

##### Medical History: #####

Uncontrolled eczema for the past decade

##### Current Medications: #####

Artificial tears as needed

#### Condition of the Disease: ####

##### Lesion Location and Size: #####

Both eyes (OU): Hypertrophy, hyperpigmentation, erythema of the lid skin, cicatricial ectropion of the lower lid with near total madarosis, 2+ nuclear sclerosis and posterior subcapsular cataracts. Fornix foreshortening and symblepharon with diffuse injection more prominent in OD. Near complete conjunctivalization of the cornea with haze entering the visual axis inferonasally.

Visual acuity: OD 20/60-1, OS 20/70-1

Intraocular pressure: OD 13 mmHg, OS 12 mmHg

#### Indications: ####

Chronic ocular itching, mucoid discharge, and decreased visual acuity.

History of atopic dermatitis and eczema, particularly uncontrolled for over a decade.

Significant periocular eczema, conjunctivalization of the cornea, and presence of symblepharon, pannus, and posterior subcapsular cataracts.

Presentation with chronic, bilateral conjunctivitis and associated periocular skin changes, such as hypertrophy, cicatricial ectropion, and madarosis.

#### Treatment: ####

##### Method 1: Early Disease Management #####

Cold Compresses: Applied to reduce inflammation and discomfort.

Preservative-Free Artificial Tears: Used to maintain ocular surface lubrication.

Mast Cell Stabilizers/Antihistamine Drops: Such as olopatadine 0.1% or lodoxamide 0.1% to control the allergic response and reduce itching.

Antihistamine Drops: Azelastine 0.05% can be used specifically for alleviating ocular itching.

##### Method 2: Advanced Disease Management #####

Topical Cyclosporine A 0.05% Eye Drops: Used to control ocular inflammation.

Topical Tacrolimus 0.03% Ointment: Applied to the eyelid skin to manage severe inflammation.

Topical Corticosteroids: Employed in more severe cases to reduce inflammation, with careful monitoring to avoid side effects.

Systemic Treatments: Oral Cyclosporine, Tacrolimus, or Corticosteroids: These may be used when topical treatments are insufficient, particularly for systemic control of atopic manifestations.

Consultation: Allergist/Dermatologist Consultation: Co-management with specialists to provide systemic therapy and follow-up.

Surgical Intervention: Boston Keratoprosthesis: Considered for visual rehabilitation in cases where corneal opacification has led to significant vision loss.

#### Precautions: ####

Long-term use of corticosteroids should be monitored carefully to avoid potential side effects such as cataract formation and increased intraocular pressure.

Patients should be educated about the importance of regular follow-up and adherence to treatment to prevent disease progression and complications.

Collaboration with dermatologists and allergists is essential for comprehensive management of the systemic manifestations of atopy.

**#### Disease Name: ####**

**Calcific Band Keratopathy**

#### Disease Description: ####

Calcific band keratopathy is a corneal degeneration characterized by the deposition of calcium, primarily in the form of hydroxyapatite, in the epithelial basement membrane, Bowman's layer, and the anterior stroma of the cornea. The condition manifests as a horizontal, gray-white opacity in the cornea that typically begins near the limbus and progresses centrally, often affecting the visual axis. As the disease advances, the deposits may become chalky-white and can extend deeper into the stroma, causing symptoms such as irritation, foreign body sensation, and recurrent epithelial erosions. Calcific band keratopathy is often associated with chronic ocular diseases, systemic conditions like hypercalcemia, or exposure to certain chemicals.

#### Patient Information: ####

##### Patient Age: #####

71 years old

##### Medical History: #####

Hypertension

Non-exudative age-related macular degeneration

##### Current Medications: #####

Lisinopril

AREDS 2 vitamin

#### Condition of the Disease: ####

##### Lesion Location and Size: #####

OD: Band-shaped, horizontal, gray-white subepithelial corneal opacity in the interpalpebral fissure with involvement of the visual axis. Pachymetry: 619 microns. IOP: 16 mmHg.

OS: Normal cornea. Pachymetry: 582 microns. IOP: 16 mmHg. Visual acuity: OD counts fingers at 1 foot, OS 20/20-2.

#### Indications: ####

Chronic ocular conditions such as uveitis, herpetic keratouveitis, and exposure keratopathy.

Systemic conditions like hyperparathyroidism, vitamin D toxicity, and sarcoidosis.

Ocular symptoms such as painless, progressive vision loss, irritation, and foreign body sensation.

Presence of horizontal, gray-white corneal opacity in the interpalpebral fissure, especially if it affects the visual axis.

#### Treatment: ####

##### Method 1: Manual Superficial Keratectomy with EDTA Chelation #####

This technique involves the manual dissection and removal of calcific deposits from the corneal surface. It is performed on an outpatient basis under topical anesthesia using an operating microscope.

The initial step is the manual debridement of the corneal epithelium and any loose calcific deposits using an Amoils scrubber and/or a 57 or 69 Beaver blade.

After debridement, a filter paper disk soaked in 3% EDTA solution is applied to the subepithelial calcium deposits for intervals of approximately 3 minutes, followed by thorough rinsing with balanced salt solution.

The EDTA application is repeated until satisfactory removal of calcium is achieved. Care is taken to avoid aggressive removal that might damage the underlying Bowman's membrane, ensuring a smooth optical surface for better visual outcomes.

Postoperatively, a bandage soft contact lens is applied, and prophylactic topical antibiotics are used until the epithelial defect heals.

##### Method 2: Excimer Laser Phototherapeutic Keratectomy (PTK) #####

PTK is used as an alternative or adjunct to manual keratectomy, particularly in cases with dense, uniform involvement of the calcific deposits across the visual axis.

The PTK technique begins with the programming of an ablation diameter (6.0 to 9.0 mm) and a sufficient ablation depth to achieve the desired removal of calcific deposits.

The corneal epithelium is manually removed over the calcific deposits, and the surface is smoothed with a Beaver blade.

The laser ablation is initiated and carried out to a depth of 30 µm or until the uniform calcium pattern begins to break up. Additional ablation cycles are performed with meticulous drying and smoothing between each cycle until most of the calcium deposits are removed.

For residual calcium and normal exposed stroma, a non-viscous lubricating drop is applied to mask the unaffected stroma while continuing the ablation.

After the procedure, mitomycin-C 0.2 mg/ml is applied to the central cornea for 12 seconds to minimize scarring risk, followed by thorough rinsing.

Postoperatively, a bandage soft contact lens is used along with topical antibiotics and steroids (e.g., prednisolone acetate 1.0%) which are tapered over 4 to 6 weeks.

#### Precautions: ####

Patients undergoing surgical treatment should be monitored for postoperative complications such as infection, scarring, and recurrence of calcific deposits.

The choice between manual keratectomy and PTK should be based on the extent and location of the calcific deposits, as well as the patient's visual needs.

In cases with underlying systemic conditions like hyperparathyroidism or sarcoidosis, appropriate systemic management is crucial to prevent recurrence of calcific band keratopathy.

**#### Disease Name: ####**

**Fuchs’ Endothelial Dystrophy**

#### Disease Description: ####

Fuchs' endothelial dystrophy is a progressive eye disease characterized by the deterioration of the endothelial cells in the cornea. This leads to corneal edema, stromal haze, and eventually, a significant reduction in visual acuity. The disease often presents with symptoms such as decreased vision, especially in the morning, glare, and difficulty driving. It is commonly associated with cataract development in the affected eyes.

#### Patient Information: ####

##### Patient Age: #####

56 years old

##### Medical History: #####

Fuchs’ endothelial dystrophy

Hyperlipidemia

Seasonal allergies

##### Current Medications: #####

None listed

#### Condition of the Disease: ####

##### Lesion Location and Size: #####

OD: 4+ confluent guttae, trace anterior stromal haze, 1+ stromal edema; Pachymetry: 645 microns; IOP: 16 mmHg.

OS: 4+ confluent guttae, trace anterior stromal haze, 1+ stromal edema; Pachymetry: 636 microns; IOP: 15 mmHg.

Best-corrected visual acuity: OD: 20/25-2, OS: 20/25-2

#### Indications: ####

The patient, a 56-year-old woman, had progressively worsening vision over the last year, primarily in the morning, along with increasing glare and difficulty driving.

Best-corrected visual acuity was 20/25-2 in both eyes, with no improvement with pinhole.

Corneal pachymetry showed thickening (645 microns OD and 636 microns OS), and slit-lamp examination revealed 4+ confluent guttae, trace anterior stromal haze, and 1+ stromal edema in both eyes.

#### Treatment: ####

##### Method 1: Cataract Removal and Descemet’s Membrane Endothelial Keratoplasty (DMEK) #####

The patient underwent staged surgery involving cataract removal followed by Descemet’s membrane endothelial keratoplasty (DMEK). The detailed surgical steps for DMEK are as follows:

Paracentesis Incisions: Make paracentesis incisions at the superior and inferior limbus.

Inject Viscoelastic: Inject a cohesive ophthalmic viscoelastic device (OVD) into the anterior chamber.

Peripheral Iridotomy: Create an inferior peripheral iridotomy.

Corneal Marking: Mark the cornea with a circular template, typically 8.5 mm in diameter, for tissue resection.

Score Descemet’s Membrane: Use a reverse Terry-Sinskey hook to score Descemet’s membrane at or just inside the circular mark.

Strip Descemet’s Membrane: Strip the membrane using the reverse Terry-Sinskey hook.

Clear Corneal Wound: Create a temporal clear corneal wound.

Remove Stripped Tissue: Remove the stripped tissue and send it to pathology.

Enlarge Wound: Enlarge the temporal wound to 3.2 mm with a crescent blade.

Remove OVD: Use an irrigation/aspiration handpiece to remove the OVD.

Inject Acetylcholine: Inject acetylcholine into the anterior chamber to constrict the pupil.

Prepare Donor Lenticule: Prepare the donor tissue, typically an 8.0 mm diameter pre-stripped graft, stained with Trypan blue and loaded into an injector.

Inject Tissue: Load the tissue into the glass injector and inject it into a shallow anterior chamber.

Suture Wound: Suture the wound with 10-0 nylon.

Position Graft: Position and unscroll the graft using gentle tapping and ensure correct orientation and centration.

Inject SF6 Gas: Inject SF6 20% beneath the graft to secure its position.

Instill Cyclopentolate: Instill cyclopentolate 1% drops to dilate the pupil and reduce the risk of postoperative complications.

Postoperative Care: Keep the anterior chamber filled with SF6 20% at elevated IOP for 10 minutes, followed by an exchange with balanced salt solution (BSS) while ensuring graft attachment. Reinject SF6 20% to leave a large bubble covering the graft (80-90% fill).

Patient Positioning: Position the patient supine as much as possible for 5 to 7 days post-operatively.

#### Precautions: ####

Post-operative positioning is crucial, requiring the patient to remain supine for 5 to 7 days to ensure graft adherence.

Careful monitoring of intraocular pressure (IOP) and graft attachment is essential, with follow-up anterior segment optical coherence tomography (OCT) recommended to assess graft adherence and detect any edge lifts.

**#### Disease Name: ####**

**Cataracts**

#### Disease Description: ####

A cataract is any opacity or discoloration of the usually clear crystalline lens, preventing light from properly passing through the lens to focus on the retina. Symptoms appear slowly and painlessly and include cloudy/blurry vision, glare, halos, perception of faded colors, yellowing/browning of vision, decreased night vision, and frequent eyeglass prescription changes.

#### Patient Information: ####

##### Patient Age: #####

Unknown

##### Medical History: #####

Unknown

##### Current Medications: #####

Unknown

#### Condition of the Disease: ####

##### Lesion Location and Size: #####

Nuclear: Innermost layer of the lens, causing progressive loss of vision and decreased perception of colors, particularly blues. Commonly age-related.

Cortical: Outermost layer, linked to diabetes, trauma, smoking, and excessive sunlight exposure. Patients experience vision loss, halos, and glare, especially noticeable at night.

Posterior Subcapsular: Just under the capsule, associated with diabetes, radiation, smoking, and long-term corticosteroid use. Rapidly progressive compared to other types, causing glare, halos, and image distortion.

#### Indications: ####

Cataract surgery is indicated when cataracts interfere with daily activities such as driving or work, or when it obscures the ophthalmologist’s view of the retina, making it difficult to monitor conditions like diabetic retinopathy, glaucoma, and macular degeneration.

#### Treatment: ####

##### Method 1: Phacoemulsification Cataract Surgery ####

Creation of a paracentesis using a blade, stabilizing the eye with a fixation ring

Instillation of topical lidocaine into the anterior chamber of the eye

Placement of an ophthalmic viscoelastic device (OVD) in the anterior chamber to maintain eye structure and protect the cornea

Creation of the main wound in the cornea to allow access to the lens

Capsulorhexis: A hole is created in the capsule to remove the lens nucleus and cortex while preserving the rest of the capsule for the IOL

Hydrodissection: A balanced salt solution is injected to separate the lens cortex from the capsule

Phacoemulsification: Ultrasound energy is used to break down the lens nucleus, and vacuum removes the fragments

Cortical Aspiration: The cortex is peeled away from the capsule

Filling the capsular bag with OVD to maintain structure

Placement of an intraocular lens (IOL) in the capsular bag

Removal of OVD from the capsular bag and anterior chamber

Hydration of the corneal incision to seal the wound

Application of antibiotic drops to prevent infection

##### Method 2: Manual Small Incision Cataract Surgery (MSICS) ####

An alternative to phacoemulsification, particularly in developing countries, where the cataract is removed whole through a small incision in the sclera, followed by IOL placement

#### Precautions: ####

Surgery should not be performed if glasses or contact lenses can meet the patient's visual needs

Complicating factors for surgery include small pupils, large brow ridge, zonular laxity, small anterior chamber, and patients on anticoagulants or alpha-1 blockers

Preoperative tests include visual acuity measurement, pupil examination, external eye examination, retinal examination, corneal curvature, and axial eye length measurement for IOL power calculation

**#### Disease Name: ####**

**Optic Nerve Sheath Meningioma**

#### Disease Description: ####

Optic nerve sheath meningioma (ONSM) is a rare, benign tumor originating from meningothelial cells surrounding the optic nerve. It presents as painless, progressive optic neuropathy that can lead to permanent vision loss due to compression of the optic nerve and its blood supply. Although typically slow-growing, this case showed unusually rapid progression over eight months, with vision going “in and out” followed by complete loss of peripheral vision.

#### Patient Information: ####

##### Patient Age: #####

34

##### Medical History: #####

The patient has a history of asthma, obstructive sleep apnea, anxiety, depression, obesity, and recent upper respiratory infections. Her family history includes Huntington’s disease in her maternal grandfather, aunt, and uncle. She is a former smoker with nicotine vape use and a history of substance abuse in remission for nine months.

##### Current Medications: #####

Albuterol inhale

Trazadone

Venlafaxine

Medroxyprogesterone injection (depot)

#### Condition of the Disease: ####

##### Lesion Location and Size: #####

Left optic nerve with enhancement of the intraorbital and intracanalicular segments. Persistent thickening of the optic nerve sheath, suggestive of optic nerve sheath meningioma, but rapid progression and the presence of oligoclonal bands in the CSF made the diagnosis atypical.

#### Indications: ####

Progressive visual symptoms, optic nerve enhancement on MRI, and findings consistent with optic nerve sheath meningioma.

#### Treatment: ####

##### Method 1: Intravenous Methylprednisolone and Oral Prednisone Taper ####

The patient was initially treated with five days of intravenous methylprednisolone, followed by an oral prednisone taper. Upon recurrent visual symptoms, further intravenous methylprednisolone treatment was given, but with minimal improvement.

##### Method 2: Optic Nerve Sheath Biopsy ####

After extensive counseling, the patient underwent an optic nerve sheath biopsy, revealing a low-grade meningothelial meningioma. The biopsy showed neoplastic cells with progesterone receptor (PR) positivity and somatostatin receptor (SSTR) immunoreactivity.

##### Method 3: Radiation Therapy ####

Due to the confirmed diagnosis of optic nerve sheath meningioma, radiation therapy was recommended as the standard treatment to preserve the remaining vision and prevent further tumor growth.

#### Precautions: ####

Careful counseling is required for patients considering optic nerve sheath biopsy due to the risk of permanent vision loss, diplopia, and other complications. Optic nerve sheath meningiomas are typically treated with radiation therapy, which has been shown to provide better outcomes for vision preservation compared to surgery or observation. The patient should be monitored for tumor progression and further visual decline.

**#### Disease Name: ####**

**Bilateral Acute Iris Transillumination (BAIT)**

#### Disease Description: ####

Bilateral Acute Iris Transillumination (BAIT) is a rare condition characterized by iris pigment release leading to iris transillumination defects and pigmentary glaucoma. It often presents with photophobia, decreased vision, and elevated intraocular pressure (IOP). BAIT has been linked to systemic fluoroquinolone therapy, particularly moxifloxacin, following an upper respiratory infection.

#### Patient Information: ####

##### Patient Age: #####

81

##### Medical History: #####

Herpetic keratitis of the right eye

Pseudoexfoliation glaucoma in both eyes

Central retinal vein occlusion in the left eye

Hypertension

Diabetes

##### Current Medications: #####

Dorzolamide-timolol three times daily in the right eye

Dorzolamide-timolol two times daily in the left eye

Brimonidine twice daily in the right eye

Latanoprost at bedtime in the right eye

Prednisolone acetate once daily in the right eye

Daily multivitamin

#### Condition of the Disease: ####

##### Lesion Location and Size: #####

The right eye showed mild blepharitis, limbal stem-cell deficiency, and advanced glaucomatous cupping with a cup-to-disc ratio greater than 0.9. The left eye exhibited patchy mid-peripheral transillumination defects and a cup-to-disc ratio of 0.3.

#### Indications: ####

The patient presented with photophobia and decreasing vision. Automated visual field testing revealed dense glaucomatous defects in the right eye. Fluorescein angiography showed normal arteriovenous transit time without macular or vascular leakage. Given her history of oral moxifloxacin use, BAIT was diagnosed as the cause of her pigmentary glaucoma.

#### Treatment: ####

##### Method 1: Medication Continuation and Surgical Options ####

The patient was advised to avoid oral fluoroquinolones and continue prednisolone acetate in the right eye. She was offered surgical options for better IOP control, including gonioscopy-assisted transluminal trabeculotomy, Omni, or a tube shunt, to be discussed with her outside glaucoma specialist.

##### Method 2: GATT Surgery ####

Ten months later, the patient underwent gonioscopy-assisted transluminal trabeculotomy (GATT) in the right eye, which was complicated by visually significant vitreous hemorrhage. She was taken off latanoprost at this time.

#### Precautions: ####

The patient should avoid fluoroquinolones, particularly moxifloxacin, due to the risk of BAIT recurrence and further pigmentary glaucoma. Long-term monitoring of IOP and management of glaucoma symptoms are essential to prevent further vision loss. The patient’s BAIT and glaucoma may require ongoing surgical intervention and follow-up with the Uveitis clinic.

**#### Disease Name: ####**

**Uveitis-Glaucoma-Hyphema (UGH) Syndrome Secondary to Schlemm’s Canal Microstent Malposition**

#### Disease Description: ####

Uveitis-Glaucoma-Hyphema (UGH) syndrome is a condition characterized by intraocular inflammation, elevated intraocular pressure (IOP), and hyphema, often caused by malpositioned intraocular devices such as Schlemm’s canal microstents. In this case, the patient experienced recurrent right eye pain and photophobia due to iris-stent contact after a combined cataract and minimally invasive glaucoma surgery (MIGS).

#### Patient Information: ####

##### Patient Age: #####

66

##### Medical History: #####

Primary open-angle glaucoma (POAG) treated with selective laser trabeculoplasty in both eyes

Osteoarthritis

Hypertension

##### Current Medications: #####

Brimonidine/timolol twice daily in the right eye

Dorzolamide twice daily in the right eye

Bimatoprost every evening in both eyes

Loteprednol four times daily in the right eye

Amlodipine

Atorvastatin

Alendronate

#### Condition of the Disease: ####

##### Lesion Location and Size: #####

Inferior iridodialysis and a malpositioned Schlemm’s canal microstent incarcerated in peripheral anterior synechiae in the right eye.

#### Indications: ####

The patient presented with persistent right eye pain and photophobia following combined cataract surgery and MIGS. Examination revealed a partially incarcerated microstent in the iris, causing recurrent iritis and elevated IOP.

#### Treatment: ####

##### Method 1: Microstent Removal ####

The patient underwent removal of the malpositioned Schlemm’s canal microstent under gonioscopic visualization. Viscodissection was used to free the peripheral anterior synechiae from the microstent inlet, and the microstent was removed using microsurgical forceps. No additional iridodialysis was created during the procedure.

#### Precautions: ####

Postoperative Management: After the microstent removal, the patient’s vision improved to 20/20 in the right eye. Topical steroids were tapered to one drop every 48 hours, but the IOP remained uncontrolled with intermittent elevations up to 25 mmHg. The patient was referred to a uveitis specialist for further evaluation, and filtering surgery was recommended due to concerns of glaucomatous progression.

Removal of a malpositioned microstent may alleviate symptomatic iritis, but inflammation may persist, requiring prolonged steroid use and possible filtering surgery for IOP control. Continuous monitoring is essential to manage inflammation and prevent glaucomatous damage.

**#### Disease Name: ####**

**Ciliochoroidal Melanoma**

#### Disease Description: ####

Ciliochoroidal melanoma is a malignant tumor arising from the ciliary body and choroid. It can lead to severe complications such as retinal detachment, neovascularization of the iris (NVI), and secondary neovascular glaucoma (NVG). The tumor is often detected through imaging and presents with features such as intraocular mass, retinal detachment, and potential metastasis.

#### Patient Information: ####

##### Patient Age: #####

71

##### Medical History: #####

Posterior vitreous detachment in the right eye

Stroke two years prior (wheelchair-bound)

Type 2 diabetes mellitus

Hypertension

Obesity

##### Current Medications: #####

Aspirin

Atenolol

Prednisone

Famotidine

#### Condition of the Disease: ####

##### Lesion Location and Size: #####

An elevated, echodense ciliochoroidal mass measuring 15 mm in thickness and 20 mm in diameter was discovered in the right eye, accompanied by a retinal detachment. MRI confirmed the presence of the intraocular mass with enhancement on gadolinium contrast, suggestive of a solid tumor.

#### Indications: ####

The patient presented with six months of painless, gradual worsening vision in the right eye, associated with redness. Examination revealed no light perception, iris neovascularization, and a dense cataract in the right eye.

#### Treatment: ####

##### Method 1: Enucleation ####

Given the presence of a large ciliochoroidal melanoma with retinal detachment and iris neovascularization, enucleation of the right eye was recommended. Pathology confirmed the diagnosis of ciliochoroidal melanoma with extraocular extension.

#### Precautions: ####

Postoperative Care and Follow-up: The patient recovered well from the enucleation and was fitted for a prosthesis. She was referred to a medical oncologist for systemic workup to evaluate for metastatic disease, which at the time of evaluation was not detected. Routine imaging follow-up was recommended to monitor for metastasis.

Patients with ciliochoroidal melanoma have a high risk of metastasis, especially to the liver, lungs, and bones. Regular imaging surveillance is essential, and genetic testing may provide additional prognostic information. Tumor size, extraocular extension, and genetic features should guide the long-term treatment plan to reduce the risk of metastatic spread.

**#### Disease Name: ####**

**Granulomatosis with Polyangiitis (GPA)**

#### Disease Description: ####

Granulomatosis with Polyangiitis (GPA) is a rare small-vessel necrotizing vasculitis that often presents with systemic involvement, including respiratory tract inflammation, pulmonary infiltrates, and glomerulonephritis. Ocular involvement is common, manifesting as orbital inflammation, exophthalmos, diplopia, and ophthalmoplegia. In this case, GPA was confirmed through biopsy after the patient presented with bilateral orbital masses, pachymeningitis, and anti-proteinase 3 (pr3)-ANCA positivity.

#### Patient Information: ####

##### Patient Age: #####

79

##### Medical History: #####

Diverticulitis

Polymyalgia rheumatica

Hypertension

Subdural hematoma

Stage 1 breast cancer (HER-2 negative, ER positive), treated with lumpectomy and radiation 10 years prior

Mature cystic teratoma, removed by hysterectomy and oophorectomy two years prior

##### Current Medications: #####

Alprazolam as needed

Bultabital-caffeine-acetaminophen as needed

Aspirin

Dexamethasone

Levetiracetam

Metoprolol

Rosuvastatin

#### Condition of the Disease: ####

##### Lesion Location and Size: #####

Bilateral orbital apical masses were discovered on MRI, with pachymeningeal enhancement and an additional mass encasing the left jugular foramen. There was also an acute infarct in the right postcentral gyrus.

#### Indications: ####

The patient presented with progressive double vision, left upper eyelid droop, and a history of intermittent confusion, speech problems, and memory deficits. Examination revealed bilateral external ophthalmoplegia, left ptosis, and reduced levator function. MRI findings raised suspicion of inflammatory or neoplastic etiologies, prompting further investigation.

#### Treatment: ####

##### Method 1: Biopsy and Corticosteroid Therapy ####

The patient underwent a left orbitotomy for biopsy of the orbital mass, which revealed small-vessel vasculitis and basophilic necrosis. She was treated with pulsed intravenous methylprednisolone for three days, followed by a prednisone taper and biweekly rituximab infusions for one month.

#### Precautions: ####

Patients with GPA should be monitored for relapse and systemic involvement, especially in the lungs and kidneys. Long-term management may require ongoing immunomodulatory therapy. Regular follow-up is essential to monitor disease progression and response to treatment.

**#### Disease Name: ####**

**Primary Eyelid Angiosarcoma**

#### Disease Description: ####

Primary eyelid angiosarcoma is a rare and aggressive vascular tumor that exhibits endothelial differentiation, with a propensity to infiltrate surrounding tissues. It is often misdiagnosed due to its variable presentation, which can include plaque-like masses, edema, or erythematous lesions. The condition is associated with a high risk of recurrence and metastasis.

#### Patient Information: ####

##### Patient Age: #####

72

##### Medical History: #####

Chronic obstructive pulmonary disease

Benign prostatic hyperplasia

Bilateral hip replacements

Lumbar spinal fusion

##### Current Medications: #####

Fluticasone-salmeterol

Finasteride

#### Condition of the Disease: ####

##### Lesion Location and Size: #####

The patient presented with a 40 x 30 mm violaceous thickening of the right upper eyelid and a 10 x 10 mm palpable nodule superior to the right lateral canthus.

#### Indications: ####

The patient presented with thickening and a hard nodule in the right upper eyelid, which was initially biopsied and revealed an atypical vascular neoplasm. Given the concern for angiosarcoma, further imaging and pathology were pursued.

#### Treatment: ####

##### Method 1: Initial Biopsy and Observation ####

An incisional biopsy revealed vascular channels lined by mildly pleomorphic cells with a low Ki-67 proliferative index, indicating well-differentiated angiosarcoma. Given the low-grade morphology, the patient was initially observed closely.

##### Method 2: Chemotherapy and Tumor Resection ####

Eleven months after the initial presentation, the patient underwent repeat biopsy due to tumor progression, followed by systemic neoadjuvant chemotherapy with taxol. Definitive tumor resection was performed, and histopathology confirmed well-differentiated angiosarcoma.

#### Precautions: ####

Angiosarcoma of the eyelid requires careful long-term monitoring due to its aggressive nature and high risk of recurrence. Close follow-up with MRI and regular clinical evaluations are essential to detect tumor regrowth or metastasis. The prognosis is generally better with early surgical resection, but multidisciplinary management involving chemotherapy and radiotherapy may be necessary in more advanced cases.

**#### Disease Name: ####**

**Post-LASIK Epithelial Ingrowth (PLEI)**

#### Disease Description: ####

Post-LASIK epithelial ingrowth (PLEI) is a rare complication that occurs when epithelial cells grow under the LASIK flap, potentially leading to irregular astigmatism and vision loss. PLEI is often triggered by trauma or flap-lift procedures years after the original LASIK surgery. Treatment is usually required when the ingrowth affects vision or causes damage to the LASIK flap.

#### Patient Information: ####

##### Patient Age: #####

52

##### Medical History: #####

LASIK surgery in both eyes over 20 years ago

Basal cell carcinoma excision of the left lower eyelid

Hyperlipidemia

##### Current Medications: #####

Rosuvastatin

#### Condition of the Disease: ####

##### Lesion Location and Size: #####

A 4 mm x 3.7 mm creamy-white opacity in the LASIK flap interface of the right eye, extending from the superior flap edge.

#### Indications: ####

The patient experienced progressive vision loss over several months following trauma to the right eye. Slit lamp examination revealed epithelial ingrowth at the LASIK flap interface, with associated irregular astigmatism.

#### Treatment: ####

##### Method 1: Flap Lift and Mechanical Debridement ####

The patient underwent mechanical debridement of the epithelial ingrowth following flap lift. The LASIK flap was sutured to prevent further displacement. Histopathology confirmed epithelial ingrowth. Postoperatively, the patient was treated with besifloxacin and prednisolone acetate.

#### Precautions: ####

Postoperative Management: Sutures were removed at two and three months postoperatively. At six months, the patient’s uncorrected visual acuity improved to 20/30 +3, with no further epithelial ingrowth.

Patients with LASIK surgery should be cautious of potential traumatic injuries that may cause flap displacement. Regular follow-ups are recommended for early detection of epithelial ingrowth, particularly in patients with a history of LASIK enhancements or flap trauma. Mechanical debridement remains the preferred treatment for visually significant cases.

**#### Disease Name: ####**

**Traumatic Globe Dislocation**

#### Disease Description: ####

Traumatic globe dislocation occurs when the eye is displaced from its normal position in the orbit, often due to blunt trauma. In this case, the globe herniated into the maxillary sinus following a motor vehicle accident. Globe dislocation can result in severe visual impairment, and immediate surgical intervention is critical for optimal visual outcomes.

#### Patient Information: ####

##### Patient Age: #####

77

##### Medical History: #####

Cataract surgery with posterior chamber intraocular lens (PCIOL) in both eyes

Osteoporosis

Gastroesophageal reflux disease

Leukocytoclastic vasculitis

##### Current Medications: #####

Omeprazole

Alendronate

#### Condition of the Disease: ####

##### Lesion Location and Size: #####

Herniation of the left globe into the maxillary sinus with orbital floor fracture and extraocular muscle transection.

#### Indications: ####

The patient presented with loss of vision in the left eye and globe displacement into the maxillary sinus following a motor vehicle accident. Examination revealed no light perception in the left eye and multiple lid lacerations.

#### Treatment: ####

##### Method 1: Surgical Globe Repositioning and Fracture Repair ####

The patient underwent surgery to reposition the herniated globe and repair the orbital floor using a porous polyethylene-coated titanium implant. Additional procedures included canthoplasty and repair of lid lacerations.

#### Precautions: ####

Postoperative Management: On the first postoperative day, the patient regained hand motion vision in the left eye, which improved to count fingers vision by week three. However, the patient experienced progressive enophthalmos and motility deficits postoperatively, requiring further intervention with a custom orbital implant at another hospital.

Traumatic globe dislocation requires immediate surgical intervention to prevent permanent vision loss. Despite successful repositioning, complications such as motility restrictions and enophthalmos can persist. Close monitoring for late complications is essential, and further corrective procedures may be necessary.

**#### Disease Name: ####**

**Infectious Scleritis (IS)**

#### Disease Description: ####

Infectious scleritis is a severe ocular disorder characterized by deep inflammation of the sclera. It often follows trauma, ocular surgery, or an adjacent infection, such as bacterial keratitis. Infectious scleritis is commonly caused by bacterial pathogens, particularly Pseudomonas spp, and may lead to significant visual morbidity if not treated promptly.

#### Patient Information: ####

##### Patient Age: #####

63

##### Medical History: #####

Cataract extraction with intraocular lens placement in both eyes

Asthma

Hyperlipidemia

Goodpasture syndrome (status post failed kidney transplantation, requiring peritoneal dialysis)

##### Current Medications: #####

Oral prednisone 2.5 mg daily

Calcium acetate three times per day with meals

Symbicort/albuterol inhalers

Aspirin 81 mg once daily

Atorvastatin 40 mg daily

#### Condition of the Disease: ####

##### Lesion Location and Size: #####

A 3 x 3-mm focal whitening of the inferior perilimbal sclera with thinning and adjacent scleral abscess at 6 o’clock in the left eye

#### Indications: ####

The patient presented with pain, redness, and decreased vision in the left eye following a diagnosis of bacterial keratitis. The condition was complicated by nocturnal lagophthalmos and was initially treated with fortified cefazolin and tobramycin topical drops. Culture results confirmed pan-sensitive Pseudomonas aeruginosa, and the patient was started on fortified tobramycin and moxifloxacin. Despite this, symptoms worsened, leading to the diagnosis of infectious scleritis.

#### Treatment: ####

##### Method 1: Topical and Systemic Antibiotics ####

The patient was treated with fortified vancomycin and tobramycin drops hourly, ciprofloxacin drops hourly, and IV ciprofloxacin. Additionally, tobramycin was administered subconjunctivally.

##### Method 2: Surgical Debridement and Scleral Patch Graft ####

The patient underwent scleral de-roofing and sclerectomy with abscess drainage, followed by the placement of an amniotic membrane scleral patch graft. A novel method was employed by inserting an intravenous angiocatheter under the graft, allowing continuous tobramycin infusion for two days.

#### Precautions: ####

Postoperative treatment included continued use of ciprofloxacin and tobramycin ointment, atropine, and topical prednisolone acetate. A temporary tarsorrhaphy was placed, and the patient was monitored closely for further improvement.

Infectious scleritis requires prompt diagnosis and aggressive treatment to prevent serious complications, including endophthalmitis, perforation, and vision loss. Early initiation of culture-guided antimicrobial therapy and surgical intervention are crucial for managing this condition. Frequent follow-up and prolonged therapy, even after clinical improvement, are essential for ensuring optimal outcomes.

**#### Disease Name: ####**

**Retinal Hemangioblastoma (RH)**

#### Disease Description: ####

Retinal hemangioblastoma is a benign, slow-growing retinal vascular tumor that typically presents as a red-orange mass on the retina with prominent feeder vessels. It can be associated with macular edema, subretinal fluid accumulation, and exudation, leading to visual disturbances such as blurred vision and metamorphopsia. This condition may occur sporadically or be linked to von Hippel-Lindau (VHL) syndrome, an autosomal dominant disorder associated with multiple tumors.

#### Patient Information: ####

##### Patient Age: #####

29

##### Medical History: #####

Myopia in both eyes

Celiac disease

##### Current Medications: #####

Pre-natal vitamins

#### Condition of the Disease: ####

##### Lesion Location and Size: #####

4 x 4 mm retinal vascular mass located at 5:00 in the left eye with dilated feeder and draining vessels and associated exudation

#### Indications: ####

The patient presented with sudden-onset blurred vision and metamorphopsia in the left eye during her 20th week of pregnancy. Examination revealed a retinal vascular mass, macular edema, and associated exudation.

#### Treatment: ####

##### Method 1: Observation (Initial) ####

Due to the patient’s pregnancy, treatment was deferred until the post-partum period.

##### Method 2: Transpupillary Photodynamic Therapy (PDT) ####

Following the cessation of breastfeeding one year post-presentation, the patient underwent PDT for the retinal hemangioblastoma. However, this led to a transient exudative response, resulting in severe cystoid macular edema and an exudative retinal detachment.

##### Method 3: Anti-VEGF Therapy ####

The patient was treated twice with intravitreal ranibizumab, which resolved the macular edema and retinal detachment.

#### Precautions: ####

Postoperative Management: The patient’s vision improved from 20/400 to 20/30 after treatment. Examination at seven years post-presentation revealed a regressed fibrotic lesion with reduced feeder vessel dilation, no macular edema, and stable vision.

Retinal hemangioblastomas can be associated with VHL syndrome, so genetic testing and thorough systemic workup are recommended for such cases. While spontaneous resolution of macular edema may occur post-partum, treatment may still be necessary to prevent recurrence of fluid accumulation and further vision loss. PDT may cause a transient exudative response, requiring close follow-up and potential use of anti-VEGF therapy to manage complications.

**#### Disease Name: ####**

**MOG Antibody-Associated Optic Neuritis**

#### Disease Description: ####

MOG antibody-associated optic neuritis is an inflammatory demyelinating disorder that affects the optic nerves and can lead to significant visual impairment. It is caused by antibodies targeting myelin oligodendrocyte glycoprotein (MOG) in the central nervous system. Unlike multiple sclerosis (MS), optic neuritis in MOG disease is often more severe, may affect both eyes simultaneously, and is associated with optic disc swelling and longer segments of optic nerve enhancement. The condition is responsive to steroid treatment, with a generally favorable visual outcome.

#### Patient Information: ####

##### Patient Age: #####

63

##### Medical History: #####

No significant past ocular or medical history

##### Current Medications: #####

None

#### Condition of the Disease: ####

##### Lesion Location and Size: #####

Right optic nerve with long segment hyperintense signal abnormality on MRI

#### Indications: ####

The patient presented with six days of vision loss in the right eye, pain with eye movements, and a history of a headache two weeks prior. Examination revealed reduced visual acuity (20/400), central scotoma, and blurred disc margins in the right eye.

#### Treatment: ####

##### Method 1: Intravenous Steroid Therapy #####

The patient was admitted to Wills Eye Hospital and treated with pulse-dose intravenous steroids, followed by an oral prednisone taper. Her visual acuity improved to 20/30 during hospitalization.

#### Precautions: ####

Monitoring and Follow-up: At follow-up visits, the patient continued to show improvement, with visual acuity reaching 20/25, and her color vision returning to normal. OCT and visual field testing showed residual optic disc edema and a small paracentral scotoma in the right eye. MOG antibody testing was positive (1:40), confirming the diagnosis.

MOG antibody disease requires careful monitoring due to the potential for recurrence and residual vision loss. High-dose corticosteroid therapy is typically effective, but plasma exchange may be considered in refractory cases. Long-term follow-up is essential to monitor for relapse and to adjust treatment if necessary.

**#### Disease Name: ####**

**Pediatric Ciliary Body Melanoma**

#### Disease Description: ####

Pediatric ciliary body melanoma is a rare, aggressive form of intraocular tumor originating from melanocytes within the ciliary body. It is less common in children but can present with distinctive symptoms like heterochromia. While pediatric cases have a more favorable prognosis compared to adult melanomas, the tumor’s location in the ciliary body tends to be associated with a poorer prognosis compared to tumors located elsewhere in the eye. These tumors can cause complications such as cataracts, visual loss, and risk of metastasis.

#### Patient Information: ####

##### Patient Age: #####

5

##### Medical History: #####

Born at full term without complications

##### Current Medications: #####

None

#### Condition of the Disease: ####

##### Lesion Location and Size: #####

The lesion is located in the left eye, originating from the inferotemporal ciliary body. It measured 16 mm by 16 mm with a thickness of 11.8 mm and a base of 13.5 mm.

#### Indications: ####

The patient presented with incidentally discovered heterochromia, with the left eye showing a darker iris color. Upon examination, an intraocular mass was identified along with vitreous hemorrhage. There were no other symptoms or reported visual changes.

#### Treatment: ####

##### Method 1: Fine Needle Aspiration Biopsy and Enucleation #####

The mass was biopsied, and the pathologic results confirmed epithelioid cell type malignant melanoma. Given the diagnosis and the size of the tumor, enucleation of the left eye was performed to remove the malignancy and prevent further complications. Pathology of the enucleated eye confirmed the diagnosis and revealed characteristic features of malignancy, including dyscohesive epithelioid cells with loosened intercellular connections.

#### Precautions: ####

Pediatric ciliary body melanoma, though rare, requires thorough investigation for genetic predispositions like BAP1 tumor predisposition syndrome. Close follow-up is essential to monitor for potential metastasis and systemic involvement. Genetic counseling and molecular analysis are recommended for risk assessment and management.

**#### Disease Name: ####**

**Torpedo Maculopathy (Incidental) with Non-necrotizing Scleritis and Anterior Uveitis of the Left Eye**

#### Disease Description: ####

Torpedo maculopathy is a rare, benign, non-vision-threatening retinal pigment epithelium (RPE) and choriocapillaris anomaly characterized by an ovoid, hypopigmented lesion in the temporal retina. The lesion is typically asymptomatic and often discovered incidentally during eye examinations. It may result from aberrant ganglion cell development or RPE abnormalities during fetal development. Although generally benign, some cases may be associated with scotomas or complications like choroidal neovascularization. In this case, the patient's torpedo maculopathy was found incidentally during evaluation for non-necrotizing anterior scleritis and anterior uveitis of the left eye.

#### Patient Information: ####

##### Patient Age: #####

47

##### Medical History: #####

Iron deficiency anemia

Hypothyroidism

Elevated p-ANCA

##### Current Medications: #####

Prednisone 40 mg daily

Methotrexate 15 mg weekly

Folic acid 1 mg daily

#### Condition of the Disease: ####

##### Lesion Location and Size: #####

Right eye: Ovoid, hyper-autofluorescent lesion located temporally in the macula, measuring about 1.5 x 0.5 disc diameters and oriented towards the fovea.

#### Indications: ####

The patient presented with decreased vision, eye pain, and photophobia in the left eye for one year. The right eye was asymptomatic, with the torpedo maculopathy discovered incidentally. Additional findings in the left eye included scleral thickening, corneal stromal thickening, and a white cataract.

#### Treatment: ####

##### Method 1: Observation for Torpedo Maculopathy; Corticosteroid and Immunosuppressive Therapy for Scleritis #####

No treatment was required for the torpedo maculopathy, given its benign nature and lack of symptoms. The patient was managed for scleritis with continued prednisone and methotrexate, with plans to transition to infliximab. Observation and regular follow-up were recommended to monitor the condition.

#### Precautions: ####

Patients with torpedo maculopathy generally require observation to ensure no progression or complications like choroidal neovascularization. The left eye's scleritis and corneal involvement should be closely monitored, particularly during transitions in immunosuppressive therapy. Regular imaging, such as fundus photography and OCT, is recommended to track changes in both conditions.

**#### Disease Name: ####**

**Multiple Evanescent White Dot Syndrome (MEWDS)-like Reaction**

#### Disease Description: ####

MEWDS is an inflammatory condition characterized by transient, multifocal white retinal lesions, often associated with visual disturbances such as floaters, blurred vision, and photopsia. It is predominantly a unilateral condition affecting young to middle-aged individuals, often following a viral prodrome. The condition is self-limited, with most patients experiencing spontaneous resolution and return of visual acuity. In this case, the MEWDS-like reaction was suspected to be triggered by the Pfizer-BioNTech Bivalent COVID-19 vaccine. The patient had a more aggressive presentation and was treated with corticosteroids, leading to improvement and resolution of the retinal lesions.

#### Patient Information: ####

##### Patient Age: #####

63

##### Medical History: #####

Breast cancer treated with mastectomy

Hypercholesterolemia

Hypertension

Gastroesophageal reflux disease

COVID-19 vaccination (Pfizer-BioNTech Bivalent)

##### Current Medications: #####

None specified

#### Condition of the Disease: ####

##### Lesion Location and Size: #####

Left eye: Multifocal, coalescing white retinal lesions extending from the posterior pole to the periphery with associated optic nerve edema.

#### Indications: ####

The patient presented with floaters and decreased vision in the left eye. There were multifocal white retinal lesions, optic nerve edema, and segmental disruption of the ellipsoid zone on imaging. Fundus autofluorescence revealed hyper-autofluorescence corresponding to the retinal lesions. A temporal association with the Pfizer-BioNTech Bivalent COVID-19 vaccine was noted.

#### Treatment: ####

##### Method 1: Corticosteroids (Systemic and Topical) #####

The patient was started on 50 mg of oral prednisone daily with a weekly taper and topical prednisolone acetate 1.0% for anterior chamber inflammation. The corticosteroid treatment resulted in rapid improvement, with visual acuity in the left eye improving from 20/80 to 20/40 within two weeks and 20/20 after six weeks.

#### Precautions: ####

Future vaccinations, particularly COVID-19 vaccines, should be carefully considered due to the risk of potential ocular side effects. The patient was advised to refrain from additional COVID-19 vaccinations and to take precautions against COVID-19 exposure. Ongoing monitoring and discussions about the balance of risks and benefits should be maintained throughout the patient-provider relationship.

**#### Disease Name: ####**

**Posterior Scleritis**

#### Disease Description: ####

Posterior scleritis is a rare, underdiagnosed inflammatory condition involving the posterior segment of the sclera. It can present with a wide variety of symptoms, including optic disc edema, choroidal folds, retinal detachment, and exudative macular detachment. Pain is often absent, and the condition may be confused with other diseases like orbital pseudotumor, neoplasms, or Graves' disease. Diagnosis is typically confirmed with B-scan ultrasonography, revealing a "T" sign caused by sub-Tenon’s space edema near the optic nerve head.

#### Patient Information: ####

##### Patient Age: #####

69

##### Medical History: #####

Hypothyroidism (40 years)

Arthralgias and joint swelling, possibly undifferentiated autoimmune disease

History of a strange round rash on her leg three years prior

Family history: Glaucoma, age-related macular degeneration, hypertension, and rheumatoid arthritis

##### Current Medications: #####

Levothyroxine

Vitamin D3

Desloratadine

Magnesium chloride

Doxycycline

#### Condition of the Disease: ####

##### Lesion Location and Size: #####

Right eye: Optic disc edema with peripapillary retinal thickening, choroidal folds

Left eye: Choroidal folds, sclero-choroidal thickening

#### Indications: ####

The patient presented with flashing lights and blurry vision in the right eye. Examination revealed optic disc edema, choroidal folds, and peripapillary retinal thickening in the right eye. Imaging and lab work ruled out infectious, inflammatory, and neoplastic causes. Despite a negative autoimmune workup, the patient’s history of arthralgias and family history of rheumatoid arthritis suggested an undifferentiated autoimmune disease as the underlying cause.

#### Treatment: ####

##### Method 1: Systemic Corticosteroids #####

The patient was treated with intravenous methylprednisolone (250 mg every six hours), followed by a tapering dose of oral prednisone (60 mg). Her vision improved from 20/400 to 20/200 after initial treatment, and further improvement was expected with continued management. The patient was also referred to a rheumatologist for further autoimmune evaluation and management.

#### Precautions: ####

Regular monitoring and follow-up with both ophthalmology and rheumatology are essential to manage underlying autoimmune disease and prevent recurrence of posterior scleritis. Ongoing evaluation of visual acuity and retinal imaging should be maintained to ensure the resolution of inflammation and prevent potential complications such as permanent vision loss.

**#### Disease Name: ####**

**Tuberculous Retinal Vasculitis**

#### Disease Description: ####

Tuberculous retinal vasculitis is a rare ocular manifestation of tuberculosis (TB) that primarily affects the retinal veins and can lead to inflammation, capillary non-perfusion, and neovascularization. It commonly occurs in younger males, especially those of Asian descent, and may present without systemic TB infection. Clinical findings include retinal perivenous sheathing, intraretinal hemorrhages, neovascularization, and vitritis. The disease can result in complications such as recurrent vitreous hemorrhages, macular edema, and retinal detachment.

#### Patient Information: ####

##### Patient Age: #####

31

##### Medical History: #####

No significant past ocular, medical, or surgical history

Recent unprotected sexual activity nine months prior

Afghan refugee, living in a refugee camp

Negative history for intravenous drug use

No family history of note

##### Current Medications: #####

None

#### Condition of the Disease: ####

##### Lesion Location and Size: #####

Right eye: Disc and peripapillary hemorrhages, perivenous sheathing, nasal retinal non-perfusion, neovascularization elsewhere (NVE), intraretinal hemorrhages localized to the nasal retina

#### Indications: ####

The patient presented with one week of painless, progressive vision loss in the right eye. Examination revealed disc and peripapillary hemorrhages, macular star exudates, neovascularization, and retinal vasculitis with vitreous cell in the right eye. The left eye was unaffected. Laboratory tests confirmed tuberculosis via a positive QuantiFERON test, and the diagnosis of tuberculous retinal vasculitis was made.

#### Treatment: ####

##### Method 1: Anti-Tuberculous Therapy and Corticosteroids #####

The patient was started on a four-drug anti-TB regimen: rifampin, isoniazid, ethambutol, and pyrazinamide. Corticosteroids were added to manage the immune response and prevent paradoxical worsening after starting anti-TB treatment. One month later, the patient showed improvement in hemorrhages, though neovascularization persisted.

#### Precautions: ####

Ongoing monitoring with retinal imaging such as fluorescein angiography and optical coherence tomography (OCT) is necessary to manage complications, including capillary non-perfusion and neovascularization. Intravitreal anti-VEGF injections and panretinal photocoagulation may be needed for managing neovascularization. Additionally, systemic work-up and adherence to the anti-TB regimen are essential for long-term disease control. Regular follow-up is necessary to track disease progression and response to therapy.

**#### Disease Name: ####**

**Arteritic Anterior Ischemic Optic Neuropathy (AAION) secondary to Giant Cell Arteritis (GCA)**

#### Disease Description: ####

Arteritic anterior ischemic optic neuropathy (AAION) is a condition where there is ischemia to the anterior portion of the optic nerve, commonly caused by giant cell arteritis (GCA), a vasculitis affecting medium and large arteries. The inflammation results in occlusion of the posterior ciliary artery, which supplies the optic nerve, leading to vision loss. GCA can have systemic manifestations, including jaw claudication, scalp tenderness, and polymyalgia rheumatica, and it primarily affects individuals over 50 years old.

#### Patient Information: ####

##### Patient Age: #####

74

##### Medical History: #####

Type 2 diabetes mellitus

Hypertension

Hyperlipidemia

Questionable history of glaucoma

Past surgical history of cholecystectomy

##### Current Medications: #####

Carvedilol

Irbesartan

Aspirin

Metformin

#### Condition of the Disease: ####

##### Lesion Location and Size: #####

Right eye: Prominent disc edema and pallor with optic nerve involvement. Fundus examination of the left eye was unremarkable except for a cup-to-disc ratio of 0.7 with a healthy rim.

#### Indications: ####

The patient presented with severe, progressive vision loss in the right eye. Symptoms included shoulder and hip pain, which are common in GCA, along with chronic toothache. Examination findings revealed optic nerve edema and pallor in the right eye, with a visual acuity of hand motion OD and 20/40 OS. MRI imaging showed right optic nerve sheath enhancement and a mass in the right jugular foramen, most consistent with a glomus jugulare tumor. Laboratory findings were inconclusive as ESR and CRP were normal, though the clinical suspicion for GCA was high.

#### Treatment: ####

##### Method 1: Steroid Therapy #####

The patient was treated with pulse-dose intravenous methylprednisolone (250 mg every six hours) followed by oral prednisone (80 mg daily). A temporal artery biopsy was performed, confirming the diagnosis of GCA. The patient completed five days of IV steroids and was discharged on a tapering dose of oral steroids.

#### Precautions: ####

Monitoring for disease progression and potential relapse is critical, as the risk of relapse in GCA is significant. Continued monitoring of inflammatory markers and visual function is necessary, along with regular follow-ups to adjust steroid dosing. Further imaging may be warranted to assess for additional vascular complications or disease recurrence. The presence of an incidental glomus jugulare tumor should be monitored, though it was assessed as benign and unrelated to the vision loss.

**#### Disease Name: ####**

**Acute Macular Neuroretinopathy (AMN)**

#### Disease Description: ####

Acute macular neuroretinopathy (AMN) is a rare retinal disorder that affects the outer retina, particularly the deep capillary plexus, resulting in characteristic wedge-shaped lesions in the macula. It often presents with sudden-onset central or paracentral scotomas in one or both eyes. The exact etiology remains unclear, but it is believed to be multifactorial, with vascular compromise playing a central role. Common triggers include viral infections, flu-like symptoms, trauma, use of vasoconstrictors, hypotension, and more recently, COVID-19 infection or vaccination.

#### Patient Information: ####

##### Patient Age: #####

41

##### Medical History: #####

Cataract extraction with intraocular lens placement in both eyes

Family history of early-onset cataracts

Recent COVID-19 infection (positive PCR test one week prior)

No history of systemic illness

##### Current Medications: #####

None

#### Condition of the Disease: ####

##### Lesion Location and Size: #####

Bilateral macular involvement with near-infrared imaging showing petaloid-shaped darkening in the macula of both eyes. OCT revealed outer retinal irregularities with disruption of the ellipsoid zone in both eyes. A hyper-reflective plaque extending from the outer plexiform layer to the outer nuclear layer was identified in the left eye.

#### Indications: ####

The patient presented with bilateral central scotomas and decreased central vision following a COVID-19 infection. His visual acuity was 20/70 OD and 20/40-2 OS. The fundus exam showed a cotton wool spot and a flame hemorrhage along the inferior arcade of the left eye, with no other visible macular changes.

#### Treatment: ####

##### Method 1: Observation and Follow-up #####

No specific treatment exists for AMN, so the patient was advised observation and follow-up. The patient was also referred to a primary care provider to investigate an underlying hypercoagulable state, as Factor V Leiden heterozygous trait was discovered during the work-up.

#### Precautions: ####

The patient should be regularly monitored for vision changes, as scotomas can persist in some patients with AMN. Regular follow-ups with OCT and visual field testing are recommended to track the progression of retinal changes. Any worsening of the symptoms should prompt re-evaluation. The patient should also follow up with their primary care provider regarding the hypercoagulable state and management of Factor V Leiden, as this may increase the risk of further vascular events.

**#### Disease Name: ####**

**Pleomorphic Adenoma (PA) of the Lacrimal Gland**

#### Disease Description: ####

Pleomorphic adenoma, also known as benign mixed tumor, is the most common benign epithelial neoplasm of the lacrimal gland. It often presents in the fourth decade of life with no significant sex predilection, though it may have a slight male predominance. Patients typically present with longstanding painless proptosis and inferonasal displacement of the globe. Imaging demonstrates a well-circumscribed mass in the lacrimal gland fossa, sometimes associated with bone remodeling but without bony erosion or destruction. Histopathologically, pleomorphic adenoma is pseudoencapsulated and consists of bilayered epithelial-myoepithelial ductules in a background of myoepithelial cell proliferation and variably myxoid and cartilaginous stroma. Foci of squamous differentiation may be present. Incompletely excised tumors tend to recur in a multifocal, multinodular fashion, no longer bounded by a pseudocapsule. Overexpression of pleomorphic adenoma gene 1 (PLAG1) or high-mobility group AT-hook 2 (HMGA2) genes is common, with corresponding PLAG1 and HMGA2 gene rearrangements.

#### Patient Information: ####

##### Patient Age: #####

14 years old

##### Medical History: #####

Non-contributory

Family history includes leukemia in the paternal grandfather

Stroke in both maternal grandparents

Hypertension in maternal grandmother

##### Current Medications: #####

None

#### Condition of the Disease: ####

##### Lesion Location and Size: #####

Mass in the left lacrimal gland, described as a large, hyperintense lesion on MRI. The mass had a circumscribed, irregular surface with no evidence of bony erosion.

#### Indications: ####

Progressive swelling and drooping of the left upper eyelid over seven months

Painless proptosis of 5 mm and inferonasal displacement of the left globe

Palpable mass in the left superolateral orbit with a history of incisional biopsy

MRI findings of a large hyperintense mass in the left lacrimal gland

Diagnosis confirmed as pleomorphic adenoma through histopathology

#### Treatment: ####

##### Method 1: Surgical Excision #####

The patient underwent complete excision of the pleomorphic adenoma via lateral orbitotomy. The mass was found to be large and associated with orbital scarring from the prior incisional biopsy. Histopathology confirmed pleomorphic adenoma without evidence of malignancy.

Following the initial surgical excision, periodic MRI scans were performed to monitor the surgical site, especially given the previous incisional biopsy. At 29 months post-surgery, increasing ptosis was noted, and MRI revealed a recurrence of the pleomorphic adenoma in the left lacrimal gland. A second orbitotomy confirmed recurrent pleomorphic adenoma, and the patient underwent stereotactic radiotherapy (SRT) (25 Gy) to the superior and lateral orbit.

After further follow-up, 2.5 years post-SRT, MRI revealed another recurrence, this time in the superior medial orbit. Surgical resection and histopathology confirmed multifocal recurrent pleomorphic adenoma, with transformation to cellular atypia, characterized by variably cellular tumor nodules. Additional SRT (25 Gy) was performed to the medial half of the orbit. Six months later, MRI identified a recurrent mass in the inferior temporal orbit, which was surgically excised. Histopathology revealed multifocal pleomorphic adenoma with foci of transformation to carcinoma ex pleomorphic adenoma (CXPA).

Due to the repeated recurrences and malignant transformation, a multidisciplinary tumor board recommended orbital exenteration. The procedure was performed after extensive discussion with the patient.

#### Precautions: ####

Patients who undergo resection of pleomorphic adenoma require long-term follow-up with regular clinical examinations and MRI scans due to the risk of recurrence and malignant transformation. Incomplete excision increases the likelihood of multifocal recurrence, which is difficult to manage and may lead to malignant transformation. Any new onset of symptoms such as blepharoptosis, proptosis, pain, or dysmotility, or the appearance of a recurrent mass on MRI, should prompt consideration for carcinoma ex pleomorphic adenoma (CXPA).

**#### Disease Name: ####**

**Ocular Toxoplasmosis**

#### Disease Description: ####

Ocular toxoplasmosis is an infection caused by the protozoan parasite Toxoplasma gondii, which can affect the retina and cause inflammation. It typically presents as unilateral retinochoroiditis with a “headlight in the fog” appearance, characterized by a fluffy white lesion with surrounding haze due to vitritis. In immunocompromised patients, the presentation can be more severe, with increased risk of multifocal disease and recurrence. The infection is generally incurable, with the bradyzoite form residing in tissue cysts being resistant to antimicrobials.

#### Patient Information: ####

##### Patient Age: #####

78 years old

##### Medical History: #####

Active chronic lymphocytic leukemia (CLL)

Diverticulitis status post-colonic resection

Hepatitis B

Congestive heart failure

Hypertension

##### Current Medications: #####

Venetoclax (BCL-2 inhibitor)

Tenofovir

Entecavir

Carvedilol

Lisinopril

Prophylactic acyclovir

Fenofibrate

Cyclobenzaprine

Omeprazole

Lorazepam

Gabapentin

Duloxetine

#### Condition of the Disease: ####

##### Lesion Location and Size: #####

Right eye with inferonasal retinal whitening, fluffy white lesion with creamy edges, and severe vitritis. An inactive chorioretinal scar in the left eye sparing the fovea.

#### Indications: ####

New-onset floaters in the right eye with no pain or photophobia

Ocular history of toxoplasmosis in the left eye, treated with intravitreal clindamycin 10 years prior

Differential diagnosis included infectious (syphilis, Lyme, toxoplasmosis), inflammatory (sarcoidosis), neoplastic (lymphoma), or iatrogenic (biologic use) etiologies

#### Treatment: ####

##### Method 1: Sub-Tenon’s Triamcinolone Injection #####

A sub-Tenon’s triamcinolone injection (20 mg/0.5 mL) was administered superotemporally in the right eye to treat suspected ocular inflammation. A follow-up examination revealed an inferonasal retinal lesion with severe vitritis.

##### Method 2: PCR Testing and Antiparasitic Treatment #####

An anterior-chamber tap was performed, and PCR testing was positive for *Toxoplasma gondii* (3,300 copies/mL). The patient was initially treated with sulfamethoxazole-trimethoprim (TMP-SMX) twice daily and intravitreal clindamycin 1 mg. Due to intolerance of TMP-SMX, the patient was switched to azithromycin 250 mg/day.

##### Method 3: Prednisone Therapy #####

The patient continued to experience blurred vision and floaters despite stable acuity. Prednisone 30 mg daily was administered with a weekly taper. Follow-up examination showed improvement in the active lesion’s borders.

#### Precautions: ####

Patients with immunosuppression, such as chronic lymphocytic leukemia, are at increased risk for atypical presentations of ocular toxoplasmosis. PCR testing is essential for diagnosing the infection in such cases, as serological tests may be unreliable due to impaired antibody production. Long-term follow-up and monitoring are necessary to manage the disease and prevent recurrence. Steroid therapy should be administered with caution in patients with ocular toxoplasmosis, as it can exacerbate the disease if antiparasitic therapy is not given concurrently.

**#### Disease Name: ####**

**Uveal Melanoma with Ocular Melanocytosis**

#### Disease Description: ####

Uveal melanoma is a malignant tumor of the eye that arises from melanocytes located in the uvea. In this case, the uveal melanoma developed in association with ocular melanocytosis, a congenital condition involving increased pigmentation of the ocular tissues such as the sclera, episclera, and uvea. Ocular melanocytosis can increase the risk of developing uveal melanoma, and patients with this condition have a higher relative risk of metastasis. Uveal melanoma with vitreous hemorrhage is rare and usually occurs when the tumor breaks through Bruch’s membrane and perforates the retina.

#### Patient Information: ####

##### Patient Age: #####

76 years old

##### Medical History: #####

Central retinal vein occlusion in the left eye in 2020

Coronary artery disease

Hypertension

Hyperlipidemia

Actinic keratosis

##### Current Medications: #####

Atorvastatin 40 mg daily

Aspirin 81 mg daily

#### Condition of the Disease: ####

##### Lesion Location and Size: #####

Intraocular mass in the right eye with a mushroom-shaped appearance. The mass measured 11 mm in basal diameter and 8 mm in thickness, with extrascleral extension. A second melanocytic nodule was present in the iris.

#### Indications: ####

Sudden decrease in vision in the right eye two months prior, which had not improved

Vitreous hemorrhage in the right eye, obscuring the view of the fundus

History of ocular melanocytosis and scleral melanocytosis

#### Treatment: ####

##### Method 1: Vitrectomy and Cytology #####

A vitrectomy was performed to clear the vitreous hemorrhage and allow for assessment of the intraocular tumor. Cytology confirmed the presence of malignant cells in the vitreous, indicative of chronic vitreous hemorrhage due to uveal melanoma.

##### Method 2: Imaging and Enucleation #####

Magnetic resonance imaging (MRI) revealed a mushroom-shaped intraocular mass with extrascleral extension. Based on the findings, enucleation of the right eye was recommended and performed. Histopathology confirmed the diagnosis of choroidal melanoma with extraocular extension and a second atypical melanocytic nodule in the iris.

#### Precautions: ####

Patients with ocular melanocytosis are at an elevated risk of developing uveal melanoma and should undergo regular ophthalmic screening every six months. Uveal melanoma associated with ocular melanocytosis has a higher relative risk of metastasis, and early detection is critical. Vitreous hemorrhage, although rare in cases of uveal melanoma, should prompt an evaluation for intraocular tumors. Ultrasonography and a thorough examination should be part of the diagnostic process in all cases of vitreous hemorrhage to rule out tumors.

**#### Disease Name: ####**

**Granulomatosis with Polyangiitis (GPA)**

#### Disease Description: ####

Granulomatosis with polyangiitis (GPA) is an autoimmune disease characterized by granulomatous inflammation of small to medium-sized blood vessels, particularly affecting the sinuses, lungs, kidneys, and occasionally the eyes. Ophthalmologic involvement occurs in 50-60% of cases, with orbital disease being the most common ocular manifestation. The disease is associated with anti-PR3 antineutrophil cytoplasmic antibodies (c-ANCA), which are present in over 90% of patients with systemic disease. Diagnosis can be made based on clinical criteria and confirmed through biopsy showing granulomatous inflammation, necrosis, and vasculitis.

#### Patient Information: ####

##### Patient Age: #####

84 years old

##### Medical History: #####

Hypertension

Depression

Pyoderma gangrenosum

Left lower lid abscess with ectropion following repair surgery

##### Current Medications: #####

Atenolol

Amlodipine

Sertraline

#### Condition of the Disease: ####

##### Lesion Location and Size: #####

Enhancing mass in the inferolateral left orbit deforming the globe surface, with preseptal soft tissue swelling. Palisading granulomatous inflammation and granulomatous vasculitis were noted on biopsy.

#### Indications: ####

Redness, pain, and discharge in the left eye, worsening despite prolonged antibiotic therapy

Left lower lid ectropion with mucopurulent discharge and conjunctival injection

Imaging revealed an orbital mass with no clear abscess, prompting biopsy

#### Treatment: ####

##### Method 1: Intravenous Steroid Therapy #####

The patient initially received intravenous corticosteroid therapy following the diagnosis of orbital inflammation. This provided temporary improvement in symptoms, including decreased discharge and conjunctival injection.

##### Method 2: Orbital Biopsy and Rituximab Therapy #####

Following the development of worsening symptoms and inconclusive conjunctival biopsy results, a deeper orbitotomy and biopsy were performed. This revealed granulomatous inflammation and vasculitis, confirming the diagnosis of GPA. The patient was started on intravenous rituximab and trimethoprim-sulfamethoxazole (TMP-SMX) along with oral prednisone. Her symptoms improved with this treatment.

##### Method 3: Surgical Correction of Ectropion #####

Due to persistent left lower lid ectropion and lagophthalmos, the patient underwent lower lid reconstruction with a full-thickness tarsal graft and release of upper eyelid retractors. These procedures were necessary to improve lid closure and prevent further complications.

#### Precautions: ####

Patients with GPA require continuous monitoring and treatment to prevent relapses. Maintenance therapy with rituximab and TMP-SMX is crucial in reducing the risk of recurrence and infection. Even after successful treatment, inflammatory damage may lead to residual complications, as seen in this patient’s ectropion and lagophthalmos. Regular follow-up is essential to manage long-term complications and ensure disease remission. Missing maintenance treatments, such as rituximab infusions, may lead to disease recurrence, as demonstrated by the patient’s relapse following a missed dose during hospitalization for COVID-19.

**#### Disease Name: ####**

**Aphakia with High Refractive Error**

#### Disease Description: ####

Aphakia refers to the absence of the lens in the eye, often resulting in significant refractive errors requiring high correction spectacles or contact lenses. It can occur due to congenital conditions, trauma, or after cataract surgery. High refractive error, such as in this patient, can impact daily life and social interactions, particularly when spectacle correction is very strong. This case involves aphakia in both eyes due to previous surgeries for idiopathic panuveitis and congenital glaucoma, leading to the need for intraocular lens (IOL) implantation to correct the refractive error.

#### Patient Information: ####

##### Patient Age: #####

18 years old

##### Medical History: #####

Idiopathic panuveitis with eosinophilia, requiring pars plana vitrectomy and cataract extraction in both eyes over 10 years ago

Congenital glaucoma in both eyes, treated with goniotomy in the left eye eight years prior

##### Current Medications: #####

None (previously used latanoprost and brimonidine for glaucoma in the left eye, discontinued three years ago)

#### Condition of the Disease: ####

##### Lesion Location and Size: #####

The patient has aphakia in both eyes with capsular openings (6 mm in the right eye and 4.5 mm in the left eye). The right eye also has a Sommerings ring cataract, and the left eye has Elshnig pearls and capsular remnants.

#### Indications: ####

The patient reported high refractive error requiring strong spectacles for the past 10 years, resulting in social isolation. Attempts to use contact lenses were unsuccessful. He expressed concerns about his appearance as he prepared to attend college.

#### Treatment: ####

##### Method 1: Sulcus IOL Fixation Attempt #####

The initial plan was to place a sulcus IOL with posterior optic capture in the left eye. However, this was unsuccessful due to the residual capsule, prompting the need for an alternative method.

##### Method 2: Intrascleral Haptic Fixation (Yamane Technique) #####

Due to the failed attempt at sulcus fixation, the decision was made to proceed with intrascleral haptic fixation using the Yamane technique. Thin-walled 30-gauge needles were used to externalize the IOL haptics through the sclera, followed by cauterization to create terminal bulbs for fixation. This was performed first in the left eye and later in the right eye. Both eyes had quiet anterior chambers and normal intraocular pressures after surgery.

##### Method 3: Final Visual Correction #####

Following successful IOL placement in both eyes, the patient achieved best-corrected visual acuity of 20/20 in both eyes. His final spectacle correction was significantly reduced compared to his previous prescription, with minimal astigmatism. This resulted in dramatic improvements in his social interactions and quality of life.

#### Precautions: ####

Patients with aphakia, especially those with previous ocular surgeries, should be carefully evaluated for IOL implantation techniques that suit their anatomical and clinical conditions. Sulcus fixation and scleral-fixated IOLs offer viable alternatives when capsular support is inadequate, but each method has its risks and technical challenges. Postoperative follow-up is crucial to monitor for complications such as IOL decentration, glaucoma, or infection. Scleral-fixated techniques like the Yamane technique offer a stable, long-term solution but require skilled surgical execution and careful planning.

**#### Disease Name: ####**

**Primary Orbital Melanoma (POM)**

#### Disease Description: ####

Primary orbital melanoma (POM) is a rare malignancy that arises from melanocytes located in the orbit. It accounts for less than 1% of primary orbital malignancies and is associated with conditions such as orbital melanocytosis and nevus of Ota. Melanomas in the orbit may present with unilateral proptosis, pain, diplopia, and visual disturbances. Genetic profiling plays a crucial role in diagnosis and treatment planning, especially in distinguishing between metastatic and primary melanoma.

#### Patient Information: ####

##### Patient Age: #####

76 years old

##### Medical History: #####

Follicular lymphoma (treated with radiation and rituximab, no recurrence)

Prostate cancer (in remission)

Cutaneous squamous cell carcinoma (treated with Mohs-guided resection)

Hypothyroidism

Hyperlipidemia

##### Current Medications: #####

Levothyroxine

Atorvastatin

#### Condition of the Disease: ####

##### Lesion Location and Size: #####

A 3.1 x 2.2 x 2.2 cm intraconal mass in the right orbit, infiltrating the muscle cone and encasing intraconal structures, with mass effect on the globe and optic nerve.

#### Indications: ####

Right upper lid swelling, pain, and tearing for three months

Mechanical ptosis causing superior visual field defects

Intermittent diplopia on downgaze

#### Treatment: ####

##### Method 1: Orbitotomy and Biopsy #####

The patient underwent an orbitotomy for a biopsy of the orbital mass. Pathology revealed an infiltrate of neoplastic, mitotically active epithelioid cells positive for Melan-A, SOX10, and S100, confirming a diagnosis of melanoma. Immunohistochemical and genetic profiling suggested a high mutational burden with NRAS and TERT mutations, and PDL1 positivity, suggesting a cutaneous- or conjunctival-like melanoma profile.

##### Method 2: Orbital Exenteration with Adjuvant Immunotherapy #####

Given the absence of metastatic disease and no identifiable primary tumor, the patient elected to undergo orbital exenteration with a multidisciplinary team. The surgery involved complete resection of the mass, with no cranial base invasion and negative margins. The patient also started on adjuvant nivolumab therapy (PDL1 inhibitor) and will undergo PET scans every three months to monitor for recurrence or metastasis.

#### Precautions: ####

Surveillance and Follow-Up: The patient’s first three-month PET scan showed no evidence of metastatic disease. He continues to receive monthly nivolumab therapy and is being closely monitored with imaging to ensure there is no recurrence.

Primary orbital melanoma is a rare and aggressive condition that requires prompt diagnosis and treatment. Due to its high recurrence rate and poor prognosis, patients must undergo regular follow-ups with imaging to monitor for metastatic spread. Genetic profiling is crucial for determining the best course of treatment, particularly the use of adjuvant immunotherapy, which may offer improved outcomes in cases with a high mutational burden.

**#### Disease Name: ####**

**Corneal Edema Secondary to Acute Endothelial Toxicity**

#### Disease Description: ####

Acute endothelial toxicity can occur due to exposure to toxic substances, such as the sap from milkweed plants. This toxicity leads to corneal edema, a buildup of fluid in the cornea caused by endothelial cell dysfunction. The corneal endothelium relies heavily on Na+/K+-ATPase pumps to maintain corneal transparency, and toxins like cardiac glycosides from milkweed inhibit this pump, leading to rapid-onset corneal edema and potential bullous keratopathy. With prompt treatment, endothelial function can recover fully.

#### Patient Information: ####

##### Patient Age: #####

38 years old

##### Medical History: #####

Gastroesophageal reflux disease (GERD)

Mild iron-deficiency anemia

Previous right dacryocystorhinostomy (10 years ago)

##### Current Medications: #####

Omeprazole

Over-the-counter iron supplement

#### Condition of the Disease: ####

##### Lesion Location and Size: #####

The right cornea showed diffuse stromal edema and 2+ Descemet’s folds with punctate epithelial erosions.

#### Indications: ####

One-day history of decreased vision and burning sensation in the right eye

Foreign body sensation and photophobia in the right eye

No history of trauma, recent illness, or similar symptoms in the left eye

#### Treatment: ####

##### Method 1: Steroid and Hypertonic Saline Drops #####

The patient was treated with loteprednol 0.5% (a topical steroid) every two hours and Muro 128 5% saline drops four times per day. This regimen was designed to reduce inflammation and control corneal edema while allowing the endothelial cells to recover.

##### Method 2: Bandage Contact Lens Placement #####

Two days after presentation, the patient developed a ruptured central bulla, which caused foreign body sensation. A bandage contact lens was placed to protect the cornea while the epithelial defect healed.

#### Precautions: ####

Follow-Up and Tapering of Drops: The patient’s epithelial defect closed, and corneal edema improved within two days. The bandage contact lens was removed, and the drops were tapered over the following days. Ten days after the initial presentation, the patient’s vision returned to 20/20, and follow-up imaging showed full recovery of endothelial cell density.

Milkweed sap contains potent toxins that can cause corneal endothelial dysfunction, leading to rapid-onset corneal edema. Early recognition and prompt treatment with steroids and hypertonic saline drops are crucial for protecting endothelial cells and promoting recovery. Failure to treat or misdiagnosis could lead to permanent endothelial damage and long-term visual impairment. Patients should be counseled on avoiding further exposure to milkweed and other potential ocular toxins.

**#### Disease Name: ####**

**Primary Ductal Adenocarcinoma of the Lacrimal Gland**

#### Disease Description: ####

Primary ductal adenocarcinoma of the lacrimal gland is a rare malignancy originating from the epithelial lining of the lacrimal gland ducts. This tumor shares histological and genotypic similarities with ductal carcinomas of the salivary glands and breast. It typically presents with proptosis, limited eye movements, and sometimes diplopia. The tumor is often slow-growing but can eventually lead to metastatic disease if untreated.

#### Patient Information: ####

##### Patient Age: #####

47 years old

##### Medical History: #####

Testosterone supplementation for low testosterone levels

##### Current Medications: #####

Testosterone

Vitamin D

#### Condition of the Disease: ####

##### Lesion Location and Size: #####

A large left orbital mass extending intraconally and extraconally, displacing the globe medially, inferiorly, and anteriorly, and involving the lateral rectus, optic nerve, and globe.

#### Indications: ####

Fullness in the left eye

Intermittent diplopia on left gaze

Decreased abduction of the left eye

Choroidal folds noted on the left fundus exam

Proptosis of the left eye, measuring 5 mm

#### Treatment: ####

##### Method 1: Left Orbitotomy and Partial Resection #####

The patient underwent left orbitotomy under general anesthesia. Intraoperatively, the mass was multiloculated, with white, cloudy fluid and thick white toothpaste-like material present. The lesion was adherent to the lateral rectus muscle and optic nerve, making complete resection impossible. A partial mass resection and biopsy were performed.

##### Method 2: Discontinuation of Testosterone Supplementation #####

The tumor was found to express the androgen receptor. Testosterone supplementation was discontinued as part of the treatment strategy.

##### Method 3: Left Orbital Exenteration and Adjuvant Radiation Therapy #####

Following the diagnosis of high-grade adenocarcinoma, the patient underwent a left orbital exenteration and was treated with adjuvant radiation therapy to reduce the risk of recurrence.

#### Precautions: ####

Due to the high potential for metastasis, patients with primary ductal adenocarcinoma of the lacrimal gland should undergo regular follow-ups. Genetic profiling, including testing for HER2 amplification and androgen receptor expression, can guide treatment decisions. While metastasis is uncommon at the time of presentation, long-term monitoring is essential, as distant metastases can develop later. Surgical resection followed by adjuvant radiation is the mainstay of treatment.

**#### Disease Name: ####**

**Fuchs’ Endothelial Corneal Dystrophy (FECD) with Postoperative Interface Haze After DMEK**

#### Disease Description: ####

Fuchs' endothelial corneal dystrophy (FECD) is a progressive condition that affects the corneal endothelium, leading to corneal edema and vision loss. Descemet’s membrane endothelial keratoplasty (DMEK) is a surgical treatment for FECD. Postoperative complications, such as interface haze, can occur after DMEK, potentially impairing visual recovery. Interface haze can be caused by inflammatory reactions or, less commonly, infections like fungal keratitis.

#### Patient Information: ####

##### Patient Age: #####

57 years old

##### Medical History: #####

Hypertension

##### Current Medications: #####

Sodium chloride 5% drops (three times daily)

#### Condition of the Disease: ####

##### Lesion Location and Size: #####

Postoperative interface haze in the left eye after DMEK

#### Indications: ####

Blurry vision, worse in the left eye

Photophobia and tearing after DMEK surgery

Migrainous left-sided headache

#### Treatment: ####

##### Method 1: Management with Corticosteroids #####

The patient was treated with difluprednate 0.05% every two hours and cyclopentolate 1% twice daily to reduce inflammation. This treatment resulted in improvement of photophobia, though tearing persisted. Corticosteroids were tapered over several months as the graft interface haze improved.

Monitoring and Possible Repeat DMEK: At postoperative month 11, the patient showed significant improvement in interface haze, though trace haze persisted. Given the gradual improvement, repeat DMEK was not immediately pursued, and the patient was monitored for potential progression.

#### Precautions: ####

Careful postoperative monitoring is necessary to differentiate between fungal interface keratitis and non-infectious inflammatory haze. AS-OCT imaging is useful for distinguishing the two, as fungal keratitis presents with focal infiltrates, whereas non-infectious inflammation appears more diffuse. Prompt treatment with corticosteroids can resolve non-infectious haze, but fungal infections may require antifungal treatment. Close follow-up and imaging are critical to ensure proper management and to avoid complications like hyperopic astigmatism or further visual deterioration.

**#### Disease Name: ####**

**Carotid-Cavernous Fistula (CCF)**

#### Disease Description: ####

A carotid-cavernous fistula (CCF) is an abnormal communication between the carotid artery and the cavernous sinus, leading to increased venous pressure in the orbit. It can cause a range of symptoms, including proptosis, orbital pain, elevated intraocular pressure, and arterialization of the conjunctival vessels. CCFs are classified as either high-flow or low-flow, depending on the type of arterial connection involved. The condition can be life-threatening if not properly managed.

#### Patient Information: ####

##### Patient Age: #####

76 years old

##### Medical History: #####

Primary open-angle glaucoma treated with latanoprost and timolol

Hypertension treated with metoprolol succinate

Hypercholesterolemia treated with atorvastatin

Prostate cancer in remission

##### Current Medications: #####

Latanoprost

Timolol

Metoprolol succinate

Atorvastatin

Aspirin

#### Condition of the Disease: ####

##### Lesion Location and Size: #####

The patient exhibited an arterialized waveform in the left superior ophthalmic vein, with 5 mm of proptosis and enlarged extraocular muscles, consistent with a dural-cavernous arteriovenous malformation.

#### Indications: ####

Left eye redness, pain, and blurred vision lasting for two months

Corkscrew injection of conjunctival vessels in the left eye

2+ clumped intraretinal hemorrhages in the left inferotemporal macula

Mild left hypertropia and diplopia on downgaze

Proptosis of the left eye measuring 5 mm

#### Treatment: ####

##### Method 1: Conservative Management #####

The patient was placed on conservative treatment, which involved compression of the ipsilateral carotid artery and jugular vein several times per day for four to six weeks. This approach is appropriate for low-flow CCFs and is aimed at encouraging spontaneous closure of the fistula.

##### Method 2: Neurosurgical Consultation and Monitoring #####

The neurosurgical team performed cerebral angiography, confirming a Type-D carotid-cavernous fistula. Given the chronic nature and low flow of the fistula, surgical or endovascular intervention was not immediately required, and the patient was monitored for symptom progression.

#### Precautions: ####

Patients with CCFs should be closely monitored for progression of symptoms, as untreated fistulas can lead to vision loss, increased intraocular pressure, optic neuropathy, and other complications. Endovascular treatment is often successful if conservative management fails, but in cases of high-flow fistulas, prompt intervention may be necessary to prevent life-threatening complications. The use of orbital Doppler ultrasound is an essential tool in diagnosing CCFs and guiding treatment decisions.

**#### Disease Name: ####**

**Cancer-Associated Retinopathy (CAR)**

#### Disease Description: ####

Cancer-associated retinopathy (CAR) is a paraneoplastic autoimmune disorder in which antiretinal antibodies attack retinal photoreceptors, leading to rapid and progressive vision loss. CAR often affects both rods and cones, resulting in decreased visual acuity, visual field deficits, nyctalopia, and photopsias. The condition is frequently associated with malignancies, particularly small-cell lung cancer, and is characterized by subacute vision loss and abnormal findings on electroretinography (ERG) and optical coherence tomography (OCT).

#### Patient Information: ####

##### Patient Age: #####

61 years old

##### Medical History: #####

Obesity (BMI > 41)

Obstructive sleep apnea on CPAP

Hyperthyroidism treated with radioactive iodine and thyroidectomy

Depression

Migraines

History of smoking (80-pack-year history, quit 5 years ago)

##### Current Medications: #####

Levothyroxine

#### Condition of the Disease: ####

##### Lesion Location and Size: #####

Disruption of the extrafoveal ellipsoid zones in both eyes on OCT

Superotemporal branch retinal artery occlusion in the right eye

Arteriolar attenuation and mild peripheral pigmentary changes in both eyes

Hollenhorst plaques observed in the inferotemporal arcade of the right eye

#### Indications: ####

Decreased central and peripheral vision over four months

Nyctalopia

Photopsias in both eyes

Systemic symptoms including fatigue, muscle aches, joint pain, and dyspnea on exertion

Elevated erythrocyte sedimentation rate (47) and C-reactive protein (16.5)

#### Treatment: ####

##### Method 1: Initial Steroid Therapy #####

The patient was initially treated with 60 mg daily oral prednisone for suspected giant cell arteritis (GCA). While he experienced mild improvement, symptoms worsened when tapering below 20 mg of prednisone.

##### Method 2: Steroid-Sparing Immunosuppressive Therapy #####

Given the patient's inability to taper prednisone without symptom recurrence, he was started on mycophenolate and tacrolimus as steroid-sparing agents. Despite this, his condition progressed, and further imaging revealed a large left lung mass.

##### Method 3: Palliative Care #####

The patient was diagnosed with stage IVB non-small cell lung cancer with metastases to the liver, adrenal glands, spine, pelvic bones, femurs, and multiple ribs. He began chemotherapy and palliative radiation but unfortunately did not survive.

#### Precautions: ####

Patients with CAR should undergo a thorough cancer workup, as vision loss often precedes cancer diagnosis. In cases where malignancy is detected, aggressive treatment of the cancer alongside immunosuppressive therapy may improve visual outcomes, but prognosis remains poor if retinal degeneration has advanced. Antiretinal antibody testing can aid in diagnosis but may not always be definitive. Collaboration with oncologists and internists is crucial in managing CAR patients, particularly when associated with underlying malignancy.

**#### Disease Name: ####**

**Ciliary Body Melanoma**

#### Disease Description: ####

Ciliary body melanoma is a rare form of uveal melanoma, accounting for only 6 percent of all uveal melanomas. These tumors often remain hidden behind the iris, making them difficult to detect until they are large and cause complications such as secondary glaucoma or lens subluxation. Diagnosis typically occurs when the tumor becomes advanced. Common symptoms include increased intraocular pressure, inflammation, and secondary glaucoma due to tumor invasion into the iridocorneal angle or other mechanisms. Ciliary body melanoma can also present as recurrent uveitis or sclerouveitis, leading to misdiagnosis.

#### Patient Information: ####

##### Patient Age: #####

27 years old

##### Medical History: #####

Hypothyroidism

Migraine headaches without ocular involvement

Multiple sclerosis in maternal aunt

Breast cancer in maternal grandmother

Lung cancer in maternal grandfather

##### Current Medications: #####

Brimonidine

Dorzolamide-timolol

Netarsudil-latanoprost

Acetazolamide

Prednisolone acetate

Valacyclovir (initially), later switched to ganciclovir

#### Condition of the Disease: ####

##### Lesion Location and Size: #####

The tumor was located in the ciliary body stroma, extending from 12 o’clock to 4 o’clock, with a thickness of 3 mm and a total length of 20 mm. It was associated with anterior chamber seeding, forming deposits and clumps in the iris, angle, and the Ahmed tube shunt.

#### Indications: ####

Recurrent episodes of uveitis and anterior chamber inflammation OS

Elevated intraocular pressure up to 56 mmHg OS

Persistent inflammation despite steroid treatment

Granulomatous keratic precipitates and hypopyon OS

Anterior synechiae and deposits in the anterior chamber

Shadow suggestive of a mass in the far temporal periphery OS

#### Treatment: ####

##### Method 1: Diagnostic Biopsy #####

Fine needle aspiration biopsy of the anterior chamber debris and ciliary body mass revealed malignant melanoma cells with a high nuclear-cytoplasmic ratio and prominent nucleoli. Immunohistochemical analysis confirmed melanocytic markers Melan-A and HMB 45. The tumor cells showed mitotic activity and tested positive for BAP1 mutation, indicating a high risk of metastasis.

##### Method 2: Enucleation #####

Due to the extensive tumor seeding into the anterior chamber and the involvement of the Ahmed valve shunt, enucleation of the left eye was performed to prevent extraocular spread. The Ahmed valve and globe were removed as a single piece to avoid tumor cell dissemination.

##### Method 3: Adjuvant Therapy #####

The patient was referred to a melanoma oncologist for consideration of adjuvant therapy with Sunitinib, a receptor protein-tyrosine kinase inhibitor, to reduce the risk of metastasis. Regular surveillance with liver function tests, liver MRI, chest X-rays, and orbital MRI is planned.

#### Precautions: ####

Patients with ciliary body melanoma, particularly those with BAP1 mutations, are at significant risk for metastatic disease. Ongoing monitoring for metastasis, particularly to the liver and lungs, is essential. Given the young age of this patient, genetic counseling and testing for germline BAP1 mutation should be pursued to evaluate for familial cancer syndromes. Aggressive treatment and regular follow-up are critical for managing high-risk uveal melanoma cases.

**#### Disease Name: ####**

**Cystoid macular edema (CME)**

#### Disease Description: ####

Cystoid macular edema (CME) is a condition where extracellular fluid accumulates in the outer plexiform and inner nuclear retinal layers, forming cystic collections between retinal septa. CME can result from a range of pathologies including uveitis, postoperative inflammation, diabetes, retinitis pigmentosa, and retinal vein occlusions. It is particularly associated with postoperative inflammation and inflammatory conditions such as uveitis and sarcoidosis, and often complicates cataract surgery.

#### Patient Information: ####

##### Patient Age: #####

68

##### Medical History: #####

Pars plana vitrectomy for retained lens material after cataract surgery

Cataract extraction with Crystalens AO in both eyes

Primary open-angle glaucoma (medically managed)

Chronic right eyelid ptosis

Bilateral uveitis attributed to sarcoidosis

Sarcoidosis diagnosed by cervical lymph node biopsy, no systemic symptoms for six years off prednisone

Hypertension

Atrial fibrillation treated with anticoagulation

##### Current Medications: #####

Difluprednate 0.05% once daily (OD)

Brimonidine 0.2% b.i.d. (OU)

Apixaban 5 mg daily

Metoprolol 50 mg b.i.d.

Atorvastatin 20 mg daily

Diltiazem 120 mg daily

#### Condition of the Disease: ####

##### Lesion Location and Size: #####

Macular edema with asteroid-like opacities in the vitreous of the right eye, without vasculitis, snowballing, or scleritis. Retinal thickening confirmed by OCT, with foveal thickness measured at 267 µm.

#### Indications: ####

Persistent CME associated with uveitis and pseudophakia, resistant to multiple treatments including steroids, anti-VEGF agents, and acetazolamide.

#### Treatment: ####

##### Method 1: Periocular Triamcinolone Acetonide #####

Three doses of periocular sub-Tenon’s triamcinolone acetonide administered over nine months. The CME persisted despite this intervention.

##### Method 2: Intravitreal Bevacizumab and Triamcinolone #####

One dose of intravitreal bevacizumab followed by two injections of intravitreal triamcinolone acetonide. The CME remained resistant.

##### Method 3: Anti-VEGF Therapy #####

Three injections of aflibercept administered. CME showed no significant improvement.

##### Method 4: Topical NSAID and Difluprednate #####

After resistance to the above treatments, the patient was treated with ketorolac 0.5% q.i.d. alongside continued difluprednate 0.05% once daily. After two years, the CME resolved with improvement in visual acuity.

#### Precautions: ####

The patient experienced one episode of ocular hypertension (30 mmHg) 21 months after the last aflibercept injection, which was controlled with timolol. Long-term use of NSAIDs and difluprednate must be monitored for side effects, including possible IOP elevations. Regular follow-up is necessary to ensure sustained resolution of CME and to monitor for potential recurrence or complications like ocular hypertension.

**#### Disease Name: ####**

**Lens-induced uveitis (Phacoantigenic and Phacolytic Uveitis)**

#### Disease Description: ####

Lens-induced uveitis is a group of intraocular inflammatory diseases characterized by an inflammatory response to lens proteins, typically after trauma or cataract surgery. Phacoantigenic uveitis is a rare autoimmune condition caused by an altered immune response to undenatured lens protein following disruption of the lens capsule, leading to a type-3 immune-complex-mediated response. Phacolytic uveitis occurs when liquefied lens cortex leaks through an intact lens capsule, leading to macrophage infiltration and blockage of the trabecular meshwork, causing secondary glaucoma.

#### Patient Information: ####

##### Patient Age: #####

54

##### Medical History: #####

Chronic angle recession from remote ocular trauma

Asymmetric glaucoma, with ocular hypertension in the fellow eye

##### Current Medications: #####

Brimonidine b.i.d. OU

Dorzolamide-timolol OU

Ofloxacin q.i.d. OD

Atropine b.i.d. OD

Prednisolone acetate every two hours while awake OD

Intravenous vancomycin

#### Condition of the Disease: ####

##### Lesion Location and Size: #####

Swollen cataractous lens with inflammation centered around the lens. Peripheral anterior synechiae closing the anterior chamber angle. Histopathology revealed ruptured lens capsule, degeneration of lens cortex, chronic nongranulomatous iritis, vitritis, and end-stage glaucomatous retinal and optic nerve atrophy.

#### Indications: ####

Chronic pain and inflammation in a blind eye due to lens-induced uveitis, resistant to medical management. The patient opted for enucleation.

#### Treatment: ####

##### Method 1: Intravitreal Injection and Systemic Antibiotics #####

The patient underwent a vitreous tap followed by intravitreal injection of vancomycin, ceftazidime, and voriconazole for suspected infectious endophthalmitis. Systemic treatment included intravenous vancomycin.

##### Method 2: Enucleation #####

Due to persistent pain and inflammation in the blind eye, enucleation was performed. Histopathology revealed features consistent with phacoantigenic uveitis and phacolytic uveitis.

#### Precautions: ####

Monitor for signs of traumatic glaucoma in the fellow eye, as patients with traumatic glaucoma in one eye have a 50% chance of developing glaucoma in the fellow eye. Regular follow-up is necessary to manage potential progression of ocular hypertension in the non-traumatized eye.

**#### Disease Name: ####**

**Tubulointerstitial Nephritis and Uveitis (TINU) syndrome**

#### Disease Description: ####

TINU is a rare autoimmune disorder characterized by the combination of tubulointerstitial nephritis and uveitis. It predominantly affects adolescent females and is likely underdiagnosed due to the nonspecific nature of the renal symptoms. Uveitis in TINU is most often limited to the anterior chamber, bilateral, and non-granulomatous. Episodes of nephritis and uveitis may occur months apart, making the diagnosis challenging.

#### Patient Information: ####

##### Patient Age: #####

23

##### Medical History: #####

History of acute kidney injury with renal biopsy confirming tubulointerstitial nephritis

First episode of bilateral anterior uveitis

##### Current Medications: #####

Prednisolone acetate 1% drops every two hours while awake

Cyclopentolate 1% twice daily

Brimonidine tartrate 0.2%/timolol maleate 0.5% drops b.i.d. (for elevated IOP)

#### Condition of the Disease: ####

##### Lesion Location and Size: #####

Bilateral anterior uveitis with 3+ cell and flare in both eyes. Posterior synechiae at 6 to 7 o'clock in the left eye after the initial episode.

#### Indications: ####

Recurrent bilateral anterior uveitis associated with tubulointerstitial nephritis. The patient required treatment for both ocular inflammation and increased IOP.

#### Treatment: ####

##### Method 1: Topical Steroids and Cycloplegics #####

The patient was initially treated with prednisolone acetate 1% drops every two hours and cyclopentolate 1% twice daily in both eyes. After tapering the steroids over two months, a relapse occurred, requiring reinitiation of prednisolone drops four times daily. A slow taper was started after the inflammation was controlled.

##### Method 2: IOP Management #####

The patient’s IOP increased to a maximum of 22 mmHg bilaterally (baseline 12 mmHg) during treatment with topical steroids. Brimonidine tartrate 0.2%/timolol maleate 0.5% drops were started b.i.d., and IOP was controlled during follow-up.

#### Precautions: ####

Patients with TINU are prone to relapses of uveitis after discontinuing topical steroids, and long-term monitoring is necessary. Steroid-induced IOP elevation should be carefully managed with appropriate IOP-lowering medications. Recurrence of nephritis should also be monitored, and systemic treatment may be required if renal function worsens.
